# Maternal health care initiatives: Causes of morbidities and mortalities in two rural districts of Upper West Region, Ghana

**DOI:** 10.1371/journal.pone.0183644

**Published:** 2017-08-30

**Authors:** Joshua Sumankuuro, Judith Crockett, Shaoyu Wang

**Affiliations:** School of Community Health, Faculty of Science, Charles Sturt University, Orange, New South Wales, Australia; National Academy of Medical Sciences, NEPAL

## Abstract

**Background:**

Maternal and neonatal morbidities and mortalities have received much attention over the years in sub-Saharan Africa; yet addressing them remains a profound challenge, no more so than in the nation of Ghana. This study focuses on finding explanations to the conditions which lead to maternal and neonatal morbidities and mortalities in rural Ghana, particularly the Upper West Region.

**Method:**

Mixed methods approach was adopted to investigate the medical and non-medical causes of maternal and neonatal morbidities and mortalities in two rural districts of the Upper West Region of Ghana. Survey questionnaires, in-depth interviews and focus group discussions were employed to collect data from: a) 80 expectant mothers (who were in their second and third trimesters, excluding those in their ninth month), b) 240 community residents and c) 13 healthcare providers (2 district directors of health services, 8 heads of health facilities and 3 nurses).

**Result:**

Morbidity and mortality during pregnancy is attributed to direct causes such urinary tract infection (48%), hypertensive disorders (4%), mental health conditions (7%), nausea (4%) and indirect related sicknesses such as anaemia (11%), malaria, HIV/AIDS, oedema and hepatitis B (26%). Socioeconomic and cultural factors are identified as significant underlying causes of these complications and to morbidity and mortality during labour and the postnatal period. Birth asphyxia and traditional beliefs and practices were major causes of neonatal deaths.

**Conclusion:**

These findings provide focused targets and open a window of opportunity for the community-based health services run by Ghana Health Service to intensify health education and promotion programmes directed at reducing risky economic activities and other cultural beliefs and practices affecting maternal and neonatal morbidity and mortality.

## Introduction

Enhanced maternal health was one of the eight Millennium Development Goals (MDGs) adopted by the international community in 2000 [1, 2, 3] as a response to global increases in maternal deaths of about 830/100,000 live births (LBs) daily in the 1990s. Notwithstanding the considerable improvement in outcomes in sub-Saharan Africa since that date, maternal mortalities in the region continue to occur at a rate of approximately 510/100,000 LBs, compared to the global average of deaths of about 210/100,000 LBs [4, 5]. Notably, Ghana shares a significant proportion of the burden [6]. Reliable information on the causes of maternal and neonatal deaths is needed to effectively channel scarce resources to improve maternal and neonatal outcomes [1].

The World Health Organisation (WHO) estimated a maternal mortality ratio (MMR) in Ghana of 500 (in 2000), 400 (in 2005) and 350/100,000LBs in 2008. Despite these reductions in maternal mortality ratio, there were over 800 institutional maternal deaths in 2009 in the country [7, 8], at which rate Ghana remains far from achieving the Sustainable Development Goal three (targets 1 and 2) [2]. For example, MMR in the Upper West Region (UWR) of Ghana was estimated at 520/100,000 (within the period of 2005 to 2010) [9].

Globally, neonatal mortalities within 28 days of life have reduced significantly in the last fifteen years, with estimated reduction of 5.1 million to 2.7 million from 1990 to 2015 [10, 3]. The majority of the deaths occur within the first month of life and constitute nearly half of all under-five mortalities [10,]. The neonatal mortality rate in Ghana is 32/1,000 [7]. UNICEF noted that a new born dies every fifteen minutes and about 30,000 dies annually [6,7,11–14].

The causes of maternal and neonatal morbidities and mortalities can be categorised into two broad groupings: direct and indirect causes [1, 5, 9]. Sepsis, haemorrhage, eclampsia, obstructed labour, abortion, and hypertensive disorders were the most common direct causes [5,9], whilst the most frequently cited indirect causes as are anaemia, malaria, hepatitis B, and HIV [5,9,11].

Research also identifies various factors underlying these more overt causes of maternal and neonatal morbidities and mortalities in less resource-endowed communities [10, 11]. For example, poor pregnancy and delivery outcomes were attributed to low levels of maternal education, high parity, low socioeconomic status, alcohol abuse among others [5, 12, 13], alongside, cultural norms and historical mindsets about maternal and newborn healthcare [11, 15, 16]. However, exploration of regionally specific cultural factors impacting on pregnancy and birth outcomes remains lacking [15,17].

Many maternal and neonatal deaths could be averted if women gave birth under skilled attendance [5, 14, 18–24]. However, the use of such attendants is often limited; in northern Ghana, approximately 37% of births received skilled attendance in 2010 [7, 19, 23], but in UWR these figures are lower, with 22.3% of women accessing skilled delivery care in 2010 [6, 20]. Both figures are low compared to the national average of 52.2% [18, 20, 25–27]. This difference is attributed in the literature to significant economic, cultural and infrastructural barriers. For example, even in locations where there is an appreciable level of understanding of the risks and danger signs in pregnancy, labour/birth and aspects in the postnatal period, expectant mothers and families still preferred utilising traditional birth attendants and cultural beliefs/practices rather than care provided at local and regional health facilities [1, 10, 12–14], which are also viewed as costly [14], dangerous [12,10] and difficult to access [1, 13].

The fact that maternal and neonatal mortality remains high in rural Ghana provided the impetus for a systematic study seeking to better understand their specific causes in two districts of UWR.

## Methods

### Study setting

The study area (Nadowli/Kaleo and Daffiama/Bussie/Issa Districts) is centrally located in the Upper West Region of Ghana. The districts have 13 sub-district structures with 29 primary healthcare facilities (13 health centres and 16 community-based health planning and service compounds (CHPS) [21, 22] and one hospital at Nadowli.

### Study design

A pragmatic research paradigm underpinned by mixed methods (surveys of expectant mothers, n = 80; interviews of health professionals, n = 13; focus groups with other community members, n = 240) was adopted to explore the causes of maternal and neonatal morbidity and mortality in the study area involving four communities in each of the two districts.

#### Community selection

The eight study communities (four in Nadowli/Kaleo and four in Daffiama/Bussie/Issa districts) were purposively selected to obtain perspectives of rural residents with varying degrees of access to curative and preventive maternal health services, ranging from no access to some access. Four of the communities were served by health centres, and four by CHPS compounds; six of these communities had no access roads to the highest referral health facility (Nadowli Hospital).

#### Participant selection

Expectant mothers:The Women in Fertility Age (WiFA) data (reproductive age range of 15–49) and the number of deliveries and projected deliveries for the period 2012 to 2014 (obtained from the health directorates), formed the basis for determining that a sample of 10 expectant mothers (those in gestational age of second and third trimesters excluding the ninth month) for each community was appropriate to achieve data saturation. A list of mothers meeting the selection criteria was obtained at the ANC unit (with approval from the Director of Health Services). Other expectant mothers not receiving ANC were identified with help from the healthcare staff and community-based health volunteers. A mix of simple random and purposive and key informant procedures was employed to identify potential participants from this pool of expectant mothers. These mothers were subsequently contacted by the researcher who provided further information about the research and then confirmed their willingness to participate in the study. Of all the mothers chosen, 67 were receiving antenatal care (ANC) and 13 were not at the time of the study. They were also drawn from different age groups and parity to provide increased diversity within the study population [25, 26,28].

Focus group participants:The identification of focus group participants involved the nonpartisan but statutorily elected representatives for the communities at the District level to purposively select ten “key informants” from each of the following groups in the community to participate in focus group discussions: opinion leaders (n = 80), non-pregnant women who had previously given birth (n = 80), youth group leaders (aged 18–35, n = 80). The sample sizes were pre-determined to facilitate data saturation and potential transferability of the study’s findings to other contexts and settings [25, 26, 29]. To ensure high levels of contribution in each group, opinions were given in turns.

Health care providers:The healthcare providers were included in the study to provide their perspectives on healthcare outcomes. Following written support from Ghana Health Service, the staff in charge of each of the health care facilities in the study area were asked to participate in the study. Three “other nurses” working at the health care facilities but not in managerial positions were purposively chosen to provide further insight into expectant mother-ANC provider relationships and uptake of medical advice.

The principal researcher comes from the study region and this facilitated establishing contacts with research participants and research communities, many of which were inaccessible by road. This common ethnic background facilitated all aspects of data collection. However, it must be noted that the selection of the research participants was strictly based on the research procedures described and not on any favour.

### Data collection

Structured quantitative surveys, focus group discussions (FGDs) and in-depth interviews (IDIs) were employed to gather data from expectant mothers, community members and health professionals respectively. Quantitative data were collected first, followed by the semi-structured FGDs and IDIs. This arrangement ensured pertinent issues raised by expectant mothers were further explored during the FGDs and IDIs.

Interviews with expectant mothers followed a set survey schedule and lasted between 45 minutes to one hour. The IDIs and FGDs lasted between 1 and 1.5 hours and ended when no new issues seemed to arise, thus, when data saturation was reached “S1 Data set”.

The FGDs were conducted at convenient venues for participants and in the local language (*Dagaare*). Using local language is indispensable given the general low literacy rate. A senior research fellow who originates from the same ethnic background as the participants supported the principal investigator as a scribe and picture-taker [27]. This facilitated data collection and ensured cues and cultural gestures relevant to the inquiry were easily identified and could be further explored.

The IDIs were conducted at scheduled locations in the health facilities in English.

All quantitative surveys, IDIs and FGDs were completed as planned, thereby resulting in a higher than anticipated response rate Table 1.

**Table 1:** Participants groups.

Participants	Age range	Number	Data type	Sex disaggregation	Number of communities
**Opinion leaders**	18–59	80	Qualitative	22 females; 58 males	8
**Non-pregnant women**	18–59	80	Qualitative	All females	8
**Youth**	18–35	80	Qualitative	40 females; 40 males	8
**Expectant mothers**	18–49	80	Quantitative	All females	8
**Healthcare staff**	25–59	13	Quantitative & qualitative	11 females; 2 males	10 (8 communities and 2 district health administrations)

Key issues addressed in surveys, FGDs and interviews included: problems during pregnancy; healthcare seeking behaviours of mothers; activities engaged in by expectant mothers; cultural beliefs and practices related to pregnancy, delivery and care of the newborn; causes of maternal and neonatal morbidities and mortalities; birth preparedness and complication readiness interventions.

### Data processing

All interviews and focus group discussions were tape-recorded with the informed consent of the participants. As the accuracy of the transcription plays a paramount role in determining the accuracy and dependability of the data, all audio recordings (apart from those with health professionals) were first transcribed in “Dagaare” and then translated into English. Interviews with healthcare staff were transcribed in English.

### Data analysis

Analysis of interview and focus group discussions (FGDs) was an iterative process which began in the field. After each interview, notes were made containing: a) notes about body language or other aspects not captured by the recording; b) emerging opinions from the participants and how they could be noted and applied to other interviews; c) what went well or not-so-well; d) what should be done differently in future interviews; e) physical observations of health facilities, surface nature of roads, interactions among participants and nurses. This interim analysis enabled the researcher to add follow up questions to the interview schedule to clarify issues as they emerged.

To facilitate analysis, quantitative data were entered into SPSS, and qualitative data into NVivo (version 7.5) and MaxQDA (version 12). Text analytical categories and themes related to “causes of complications, home births, causes of morbidities and mortalities, risk factors of poor health outcomes” were developed from *a priori* codes in correlation with quantitative variables derived from literature and experience [13,20].

Interconnected themes subsequently emerged from the data (interview/FGDs transcripts, direct notes, field observations/reflections), which were read and re-read to identify and index themes and categories. Computerised coding (facilitated by use of Nvivo and MaxQDA) and repeated manual searches were conducted to ensure all salient texts including participant quotes were identified to support the *priori/*emerged major and sub-themes, and to identify other themes. Participant quotes were subsequently chosen to support the themes “S1 & S2 Data set”. Finally, both the predetermined and emerged themes were pooled together to address the research question: ‘What are the causes of maternal and neonatal morbidities in the study areas’?

### Ethical considerations

Charles Sturt University Human Research Ethical Committee granted approval for the conduct of the study (Protocol number: 2016/013). The study was supported both verbally and in writing by the Ghana Health Service and the District Assemblies. Written informed consent was obtained from all participants.

## Results

### Parity and rates of miscarriage

Eighty percent (n = 64) and 20 percent (n = 16) of participants were multiparous and prime mothers, respectively.

Fourteen expectant mothers (17.5%) had experienced a miscarriage(s), 6 (7.5%) in their first pregnancy, 4 (5%) in their second pregnancy, and 3 in their fourth or greater pregnancy. Miscarriages were perceived by pregnant women as ‘normal’ sickness rather than a complication because the mothers would ‘certainly conceive’ again.

We know the risks such as complications, miscarriages and loss of lives sometimes. As for miscarriages, we know we will get pregnant in few months’ time again.

[FGDs, non-pregnant women, Bussie]

### Complications in past pregnancy, childbirth and postpartum

Of all multiparous mothers (64), more than half (73.4%) of them complain of complications in all stages of the gestation. nearly two-thirds (n = 27, 33.8%) had experienced complications during a past pregnancy, about one-quarter in labour/childbirth (n = 15 18.3%), and 6.3% (5) in the early postpartum period (birth to six weeks). One woman reported complications in all three stages.

#### Complications in past pregnancy

The complications in past pregnancy were identified as urinary tract infections (45%); pre-eclampsia (21.1%); breech presentation (15.8%). Other undifferentiated problems constituted 63.2%. Of the nineteen mothers (who experienced complications), anaemia, UTIs, nausea/vomiting and Malaria, rheumatism, HIV/AIDS, oedema and hepatitis B (36.4) were also identified as the cause of the complications.

#### Complications in past labour/childbirth

Fifteen mothers (18.8%) had experienced complications during a previous labour and delivery; eight (10%) with prolonged labour (greater than 12 hours), 3 (3.8%) with profuse vaginal bleeding, 2 (2.5%) with general body weakness (2.5%). ‘Other’ experiences constituted 2.5%.

#### Postpartum complications

Severe vaginal bleeding (1.3%), foul smelling discharge (1.3%), and other undifferentiated signs (3.8%) were experienced during the early postnatal period (birth to six weeks).

#### Reasons for hospital admission during pregnancy, childbirth and postpartum

Twenty-seven (33.8%) expectant mothers reported being admitted to hospital for direct and indirect causes of complications. Of this number, urinary tract infections (n = 13, 48.1%), anaemia (n = 3, 11.1%), mental health conditions (n = 2, 7.4%), hypertensive disorders (n = 1, 4%), nausea (n = 1, 3.7%) were the direct causes. Indirect causes of hospitalisation included malaria, HIV, oedema, hepatitis B (25.9%).

#### Recognition of complications

Unfortunately, many of the complications (n = 28, 35%) were recognised at the time “when it had already begun”. Only two expectant mothers recognised complications due to early warning signs, 3.8% (3) were informed during antenatal care and two mothers were informed on admission at the hospital.

### Causes of maternal morbidity and mortality

The communities and healthcare providers have a diverse array of explanations for maternal and neonatal morbidities and mortalities in the study areas. These are presented thematically below.

#### Poor utilisation of prenatal and skilled maternity care

It was common for health professionals to attribute poor pregnancy outcomes to lack of participation in antenatal care.

It is through the early antenatal care we also refer them for laboratory investigations, for early detection of any abnormalities for early treatment or management. However, when expectant mothers report late especially in the second or third trimester and there is any abnormality, it will be difficult to treat it because the harm might have been caused already to her and the unborn baby.

[IDIs, facility head, Jimpensi CHPS]

A women’s group also noted that:

Stillbirths are caused by the refusal of expectant mothers to seek maternal health care at the clinic or hospital. Once a pregnant woman fails to receive ANC, it affects the health of the foetus.

[FGDs, non-pregnant women, Charikpong]

A midwife observed:

The clinic further gives essential medicines to all expectant mothers, which some do not take. Women who do not take those medicines do not have safe pregnancies.

**Fig 1:**
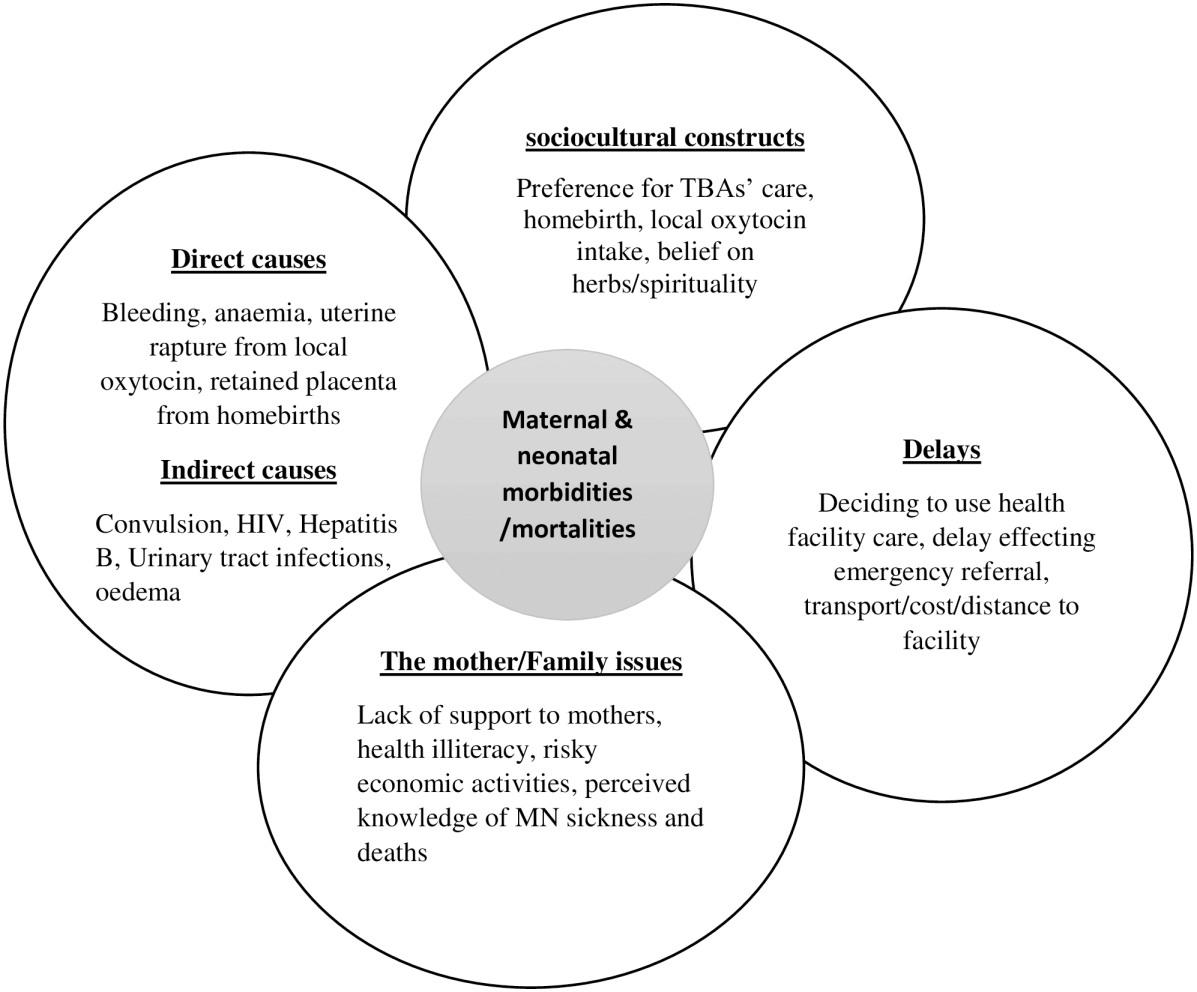
Interrelationships among the causes of poor pregnancy and birth outcomes.

There are many reasons given as to why expectant mothers do not participate in antenatal care or utilise health care at facilities and professionals during delivery, including distance, cost, cultural concerns over appropriateness of ANC, preferences for use of local oxytocin and home birth, and a lack of involvement (actively assisting to reach facility and providing for the needs of expectant mothers) of family members, especially husbands Fig 1.

#### Distance and means of transport to referral hospitals

The communities are distant from the referral hospital, which, when coupled with the poor road network, causes delays for expectant mothers in reaching the facility in times of emergency, leading to poor birth outcomes or deaths.

Jang is under Nadowli/Kaleo District but closer to Wa than Nadowli, therefore, my wife once had a childbirth at home but was sick. Considering her health situation at the time, we found Wa hospital to be closer. However, she died before we reached the facility. The major cause of maternal deaths is a result of the distance to relevant care facility during complications and emergency referrals, getting means to reach hospital becomes another challenge. Non-availability of means of transport further worsens the condition of the expectant mother, which often times lead to poor birth outcomes.

[FGDs, opinion leaders, Jang]

Two other groups said:

One main issue is the means of transport during emergency referrals to Nadowli hospital. The delay in reaching the facility leads to wayside stillbirths. Others are caused by the “shaking on the rough road” by the tricycle. It affects the baby by the time they get to the hospital. … Other expectant mothers reach the facility to discover the unborn baby is dead already.

[FGDs, opinion leaders, Woggu]

#### Income generating activities

Charcoal burning, farming activities, local wine preparation (“pito” brewing) and other economic activities are the dominant livelihood and income generating activities in these communities.

Thirty-five (43.8%) expectant mothers believed participating in these activities contributed to complications (including bleeding, prolonged labour, pre-eclampsia) they had experienced previously, identifying that they occurred after carrying head load (n = 7, 20%), upon returning from farm work (n = 7, 20%), after brewing local wine (nationally called “pito”) (n = 3, 8.6%), engaging in charcoal burning (n = 2, 5.7%), or after falling or fetching firewood (n = 3, 8.6%). ‘Other’ origins were identified as contributing to 37.1% of complications.

Focus group participants also identified these activities as risky, whilst at the same time acknowledging that participation in these activities is considered essential.

As I sit, I brew pito, so when I conceive I still brew pito although the heat from the fire could affect my health. If I don’t brew pito, I won’t get money to grind flour, buy salt, and other food ingredients. Although I feel the pains and suffer the risks of the fire, I still brew, simply because I have no other option to make income. During the rainy season, pregnant women do sowing of seeds. Unlucky ones even till and weed with hoe whilst pregnant. So, as we continue to engage in these menial jobs, usually it gets to a point where we experience severe pains and complications. However, we endure because it is the same work for every woman whether pregnant or not…. We also burn charcoal. I burnt charcoal throughout my recent pregnancy and suffered unusual pains during labour but was lucky to have a successful childbirth.

[FGDs, youth leaders, Woggu]

Regardless of the dangers involved, expectant mothers climb trees to harvest fruits and firewood.

Expectant mothers do climb trees to cut firewood or harvest “dawadawa” fruits. These could result from pressure from the husband as being lazy. So, in the depressed state of mind, expectant mothers do all kinds of unusual activities even if we could die and rest. These often lead to complications and loss of a pregnancy or both.

[FGDs, non-pregnant women, Bussie]

Head loading of farm products, charcoal, potable water and firewood by expectant mothers was causing stillbirths in some communities.

An expectant mother once went to the farm and to cut firewood. After she had cut them, made efforts to carry it up her head and fell with it. A hunter spotted her struggling to convey the wood on head load… He supported her to carry the wood home. The next day, she had a stillbirth to a set of twins, and both were boys. The expectant mother was a prime. One came out at Duang dead whilst the second was felt kicking but there were no means to rushed her to Issa health centre. When the family finally got her to Nadowli Hospital, the other baby also died. The mother suffered some complications but could conceive subsequently.

[FGDs, opinion leaders, Duang].

According to the interviews, illegal mining (termed as “galamsey” in Ghana) also accounted for the loss of some expectant mothers and or death of the baby.

We still experience stillbirths so much in our community. Young women are prepared to sacrifice their lives for money. Therefore, even when pregnant, they engage in wood logging, charcoal burning and worst of it “galamsey mining” operations to create wealth. They indulge in risky businesses with the view that, their husbands do not provide for their needs. The majority of expectant mothers who engage in charcoal burning and illegal mining activities usually have stillbirths. The heat and the dust cause preterm births.

[FGDs, non-pregnant women, Jimpensi/Kenkelley]

Others attributed participation in these activities to lack of support from families, rather than to the expectant mother:

Some will still engage in charcoal burning despite the risk of the fierce naked fires, and even pumping the borehole is not advised to do by expectant mothers, yet they still do it. But a lot of them do these jobs because families do not support them in doing household chores and exempting them from farming activities. Expectant mothers have this adage “living in hunger is death in itself” hence we will do all we can to eat.

[FGDs, non-pregnant women, Jang]

Other participants in focus groups raised concerns focused on the involvement of men in relation to pregnancy, particularly their expectation that women must continue to perform their economic responsibilities regardless of the stage of pregnancy.

For example, ANC is mostly conducted on a group basis. Therefore, fixed days were allotted for ANC, but some men were reported compelling their wives to undertake farm work on ANC days.

The issue of midwife shortage and expectant mothers’ failure to receive care are not the only issues causing poor pregnancy outcomes. Some men are ignorant about the relevance of maternal health care seeking. For instance, during the rainy season, expectant mothers are stopped from going for care to being on the farm with the husband. Other men have the notion, “ANC can be received any day, and the moisture in the farm is available for few days, hence, and let us go to farm”. For fear of the partner, some expectant mothers comply with him.

[FGDs, opinion leaders, Jang]

Women in the Bussie community made similar observations:

In Bussie community, the moment a woman conceives, the man behaves as if he is not responsible for the conception. Starting from the laboratory investigations until childbirth, we fend for ourselves in everything. They don’t even support (financially and or with means of transport) us to go to Nadowli hospital for laboratory tests and scans. During farm work, when we are in pains, we are expected and some men insist on us to continue to work. When we complain, we are tagged as being lazy or pretentious.

[FGDs, non-pregnant women, Bussie]

The discussions reported that the majority of husbands/partners do not give expectant mothers the requisite psychological and physiological support throughout the period of pregnancy. Similar issues actually led to a maternal death, two years preceding this project.

Stillbirths and maternal deaths could also come from petty quarrels at home. These could cause depression in the mother causing poor progress of pregnancy. Some mothers attempt terminating the pregnancies due to the quarrels which often times lead to stillbirths or loss of both lives. We recorded a maternal death like that two years ago, she was even beaten by the husband which led to complications and was referred to Wa and she died there. We were told she had so much internal bleeding, but because she could not reach the facility early, they were unable to treat it.

[FGDs, non-pregnant women, Charikpong]

A number of young people also identified the importance of strong relationships in ensuring a healthy pregnancy.

The healthy conversation also promotes healthy and safe pregnancies. Once the expectant mother is happy with the husband and maintains healthy communication at all times, it will give her peace of mind and ensures safety her conception.

[FGDs, youth, Charikpong]

#### Preference for use of local oxytocin and other herbs

Health professionals attributed certain complications to the use of oxytocin and other herbs:

Once the expected date of delivery elapses by just a day, expectant mothers don’t come to the facility, rather they go to the TBAs for “hot” local oxytocin. It’s taken orally and also smeared on the tummy to speed up the process of labour, thus to induce labour early. In such instance, expectant mothers get irregular contractions, severe ones and when they don’t get to the facility early for immediate care, the breadth of the foetus will reduce. So, the child can asphyxiate. In case it’s a home birth, the baby may die.

[IDIs, facility head, midwife]

Another midwife noted:

When they feel a little pain, they take the local oxytocin. Sometimes it’s not yet labour and they take it. When the labour then set in, they go through severe pains, resulting to wailing and crying and for such cases, we just refer them to Nadowli hospital, because for Woggu CHPS there is nothing “I can do to help them”.

[IDIs, midwife, health facility in-charge, Woggu CHPS]

Despite the potential for negative outcomes, many community participants expressed a number of reasons why the use of local oxytocin and other herbs was preferred over ‘medical’ interventions during pregnancy and labour.

The first was to reposition the foetus:

We have the Calabash ladle practice for breech presentation of foetus. Adult women administer the herbal medicine to the ladle and make a line across the tummy of the expectant mother. Anytime, this is done, the foetus repositions itself

Second, to trigger labour and ensure a safe childbirth:

When pregnancy is overdue, there are herbs—local oxytocin, which is applied on the expectant mothers’ tummy and orally, triggering immediate labour.

The local oxytocin is more potent for positive birth outcomes than what is given at the clinic now…Labour is also very easy when the “hot” local oxytocin is taken orally. We, however, wish the government could endorse it for expectant mothers to freely administer it.

[FGDs, opinion leaders, Naro/Korinyiri]

Local oxytocin was seeing as a means to stop pregnancy threats and complications:

We have herbs such as local oxytocin, which is administered on expectant mothers to ensure safety conception and smooth childbirth. The local oxytocin is a multi purpose herb. It has the potency to stop pregnancy threats and complications.

Finally, it was believed that its use would bring good health to the neonate and the mother:

When a neonate falls sick, we have some leaves that are boiled into “syrup” and administered orally and through the bath to restore good health. The “syrup” is usually administered when the clinic is unable to treat it.

[FGDs, opinion leaders, Nanvilli/Siruu]

#### Spiritual treatments and herbal remedies

It is not unusual for communities to believe that spiritual interventions are more effective than medical interventions in treating sickness in pregnant and postpartum women, and the neonate.

When a newborn and expectant mother fall sick, we do believe it is not an ailment modern medicine can cure. For instance, skin rashes, pimples and boils are believed to be caused by the “god of blacksmiths”, so the elders will have to pacify the gods and make a metal bangle for the patient’s wrists and feet for them to recover good health. White scalp on the skin is likened to our traditional cowries’ usage. Therefore, incantations are made on a perforated cowry and hanged on the neck of the sick person to be healed. Some families combine both modern medicines and traditional medicines. Others do not seek care from the hospital in such condition.

[FGDs, youth, Charikpong]

However, health professionals and some of the younger participants felt this often-caused delays that resulted in the death of the mother or baby:

For example, convulsion has potent medicines at the clinic, but the old women will apply herbs until the baby condition worsens before it is taken to the clinic. I do not like the beliefs on MNH care.

[FGDs, Youth leaders, Charikpong]

#### Homebirth

Homebirth as a cause of morbidity and mortality:Several older non-pregnant women noted during group discussions that home births may lead to severe bleeding and retained placenta which could cause death.

Retained placenta is also another prominent cause of some deaths in our community. The retained placenta is common among the home births. When she is delayed in reaching the facility, she bleeds excessively by the time they reach the facility.

[FGDs, non-pregnant women, Charikpong]

In Bussie, Elder women identified a similar problem:

Another cause is trapped placenta. During home births, over-pulling of the placenta leaves a thin lining in the uterus. Failure to remove it or if it is unnoticed could lead to sudden death of the mother, many days after successful childbirth. We discourage home births nowadays because of the cutting of the umbilical cord and removal of the placenta. The placenta also contains some fluid which should not excrete into the uterus. If proper care is not taken during home births, these could be released into the mother causing illness that may lead to loss of her life. What I will say is that “home birth kills”.

[FGDs, non-pregnant women, Bussie]

Concerns were also raised about the TBAs contributing to poor pregnancy and delivery outcomes in Jimpensi/Kenkelley community.

The TBAs were also a factor to some complications which led to death. Sometimes, there will be no sign of the baby coming out, but they will apply the “hot local oxytocin” and forced them (pregnant women) to “push”, regardless of whether the baby was in breech position.

[FGDs, non-pregnant women, Jimpensi/Kenkelley]

TBAs sometimes palpate the womb so hard such that, some expectant mothers do get “black-out”.

[FGDs, non-pregnant women, Bussie]

Preference for home birth: Given the risks of home birth, participants were asked why home births, many with the assistance of a traditional birth attendant (TBA) continued to be preferred over delivery at health care centres.

First, there are concerns related to the distance to the healthcare facility, and over whether the midwife will be present on arrival:

We embrace the services provided by TBAs because of the distance to Nadowli hospital and absenteeism of the midwife at our clinic.

[FGDs, non-pregnant women, Naro/Korinyiri]

Some concerns related specifically to the cost of medical care and the cost of finding transport to the facility. One mother observed:

I have requested for money to seek maternal health care from my husband and was not given. I had to struggle to acquire and fuel a bike to go to Nadowli hospital for laboratory tests.

Second, TBA’s are perceived to be more skilled than the health professionals:

*Traditional birth attendants were more skilful than the nurses we have now. The TBAs rendered antenatal care and there were usually no miscarriages during their time as it happens now*.

[FGDs, a male, opinion leaders, Naro/Korinyiri]

Third, home birth also facilitates the use local oxytocin (see above):

the local oxytocin is more potent than what is given at the clinic now.

[FGDs, a male, opinion leaders, Naro/Korinyiri]

If a mother or family member had already had a safe home birth it was assumed that subsequent home deliveries would also have positive outcomes:

our previous home birth was smooth therefore subsequent births will also be successful….

[FGDs, non-pregnant women, Charikpong]

Other men have been cited saying their mothers did not receive ANC but gave birth successfully.

[FGDs, non-pregnant woman]

#### Alcohol consumption

Alcohol consumption was identified as a high-risk factor in pregnancy and childbirth. Some expectant mothers substituted the recommended nutritious food intake with alcohol “akpeteshie” consumption, which was seen to contribute to stillbirths.

Some expectant mothers are put on a specific diet to boost their immune system and or regulate their haemoglobin level, and these must be obeyed for the safety of her pregnancy and life. Some women are usually placed on fruits and vegetable diet but we have seen pregnant women rather drinking alcohol instead of eating nutritious food.

[a male, FGDs, youth leaders, Charikpong]

#### Young maternal age

In some communities, girls under 18 years were reported suffering complications because of their young age; their falling pregnant appeared to be related to an interest in exploring sexual activity as a result of limited knowledge of sex education.

We also have some girls who are below 18 years but willing to test “everything” adults do. Instances where such girls conceive, it results to one complication to another and many are unable to deliver normally. Minors are less developed to contain pregnancy, so most are unable to have normal childbirth resulting in CS births and the associated effects including loss of lives in the process.

[FGDs, non-pregnant women, Jang]

#### Unsafe abortions

Although abortion is legal in Ghana, unsafe abortions caused maternal deaths in some communities.

There was a case of maternal death which, I learnt she tried to abort and lost her life on referral to Nadowli Hospital.

[IDIs, facility head, midwife]

Many of these were carried out by young girls and unmarried women as a way of preventing public ridicule and disgrace.

#### Poor health care for newborns resulting in poor neonatal outcomes

The in-depth interviews (IDIs) reported residents engaged in traditional care and practices such as giving the neonates cold water bath in their first few minutes at birth…

which can affect the health condition of the neonate. The baby can get cold out of that and can even suffer pneumonia. Most children die from cold in the first initial days because the uterus is usually warm at birth and have to be maintained, hence, when they go to expose the neonate to cold water, the child can die.

[IDIs, facility head, midwife]

A related issue occurs in the warm season:

When they give birth, the baby sweat, so when that happens, they think the baby must be exposed to cold….

[IDIs, facility head, midwife, Naro/Korinyiri]

Another practice is the application of shea butter to the baby’s cord and exposure to cold upon the feeling of little warmth on the baby.

They believe in using shea butter to apply on the baby’s cord. If the shea butter is not well processed, the child could get sepsis infection because it is on the umbilicus they apply it on. The baby could even suffer tetanus infection out of that.

[IDIs, facility head, midwife, Naro/Korinyiri]

## Discussion

This study identifies a diverse array of causes of maternal and neonatal morbidities and mortalities in a rural setting in Ghana from the perspectives of expectant mothers, community leaders and health professionals.

The number of expectant mothers who experienced miscarriages and complications in their first and subsequent pregnancies was significant, with many participants identifying themselves as having experienced complications such as pre-eclampsia, breech presentations, anaemia, urinary tract infections (UTIs) mental health issues, HIV infections, rheumatism, hepatitis B, low HB level in previous pregnancies. These are consistent with other studies [13,14], including those reported in an autopsy study in Wa Regional Hospital and Korle-Bu Hospital in Ghana and the Lao People’s Democratic Republic [9, 14, 27].

Whilst some of these problems can be attributed to the physiological changes of pregnancy, they may be compounded by a diversity of cultural, economic and service related factors, such as lack of ANC, delays in seeking treatment and failure of husbands or partners to receive corresponding treatment. For instance, many expectant mothers utilised the services of traditional birth attendants during complications such as breech presentation and pre-eclampsia, and during home births despite the existence of a policy on free maternity and delivery care [7, 20, 23] and the awareness of the dangers of home birth (such as prolonged labours, retained placenta) and the potential value of antenatal care. The reasons for these preferences were complex and often premised on cultural, economic and accessibility reasons related to the nature of skilled health care. These results are similar to those in another study in Ghana [24], a local district in South Africa [20]. Similar findings were identified in the Rufiji district in Tanzania [12] and in the Peoples’ Republic of Lao, where women understood the importance of ANC but did not participate in ANC due to specific cultural beliefs associated with ANC [14], which ultimately led to increased maternal and infant morbidity and mortality.

Ongoing reliance on unapproved medications and cultural practices (such as the use of local oxytocin and other herbal remedies, cold baths, application of shea butter) to solve complications and sicknesses in pregnancy and postpartum are also contributing to above average mortality rates of mothers and the neonate [29,30]. The negative impacts of regionally specific culturally based herbal and spiritual interventions are common in other locations, such as Kassena-Nankana in Upper East Region (UER) and Uganda [10,29]. The difference between the findings in this study and those of UER could be that women in the UER were compelled by families to receive concoctions before seeking care at the health facility if the symptoms persisted [10].

Expectant mothers were often expected to engage in economically and culturally significant yet potentially dangerous activities, despite potential risks to their health. This was attributed to the need to provide income and food for themselves and their families and the perceived failure of husbands and family members to provide adequate support for the expectant mother. Similar findings were reported in other studies [6, 12, 15, 20, 29], and concurs with the view that targeting. husband/men—in maternal healthcare education strategies will be needed if traditional family norms are to change significantly [18,31].

## Conclusion

The key finding of this study is that the expectant mothers are subjected to profound tensions between modern healthcare practices, traditional cultural beliefs and economic imperatives at an individual, familial and community level and compounded by national policy decisions. Reliance on cultural beliefs and practices during pregnancy, including the pervasive use of traditional herbal medicines as a substitute for modern maternal and neonatal health care in the study areas, alongside a reluctance to utilise antenatal care and ongoing engagement in risky activities suggest the need to refocus existing health education and promotion activities in the rural communities, particularly those targeted at young people and men. Further research to gain an understanding of regional values, beliefs, attitudes, norms and behaviours attached to pregnancy and the use of skilled health care, particularly to the participation of men in their partner’s pregnancy, will be critical to the success of such initiatives. Ensuring affordable and ready access to health care facilities, including the provision of improved transfer or mothers and neonates during emergencies, will also be essential, but before such strategies are implemented, further studies are required into the ability of health care services to meet the needs of expectant mothers (and their families) in a culturally appropriate and economically viable way. Given that existing policy initiatives are currently viewed with reservation within communities, attempts to change deep seated values, beliefs, attitudes and practices of mothers, their partners, family and the broader community are unlikely to succeed if health care services are unable to manage resultant increased demand.

There are two main limitations of the study. The magnitude of some cultural issues may have been underestimated during FGDs and by expectant mothers due to the need to uphold cultural values that might otherwise be perceived as negative. Such influences could have more damaging effects on the mothers than were identified in the group discussions. The extent to which the culturally specific findings made in the study can be extrapolated to other communities may also be limited.

While remedying the diversity of cultural factors and risky income generating activities posing significant threats to safe pregnancies and birth outcomes in the face of underlying poverty, the subsistence nature of the local economy, long-standing gender inequality and a struggling public health care system is likely to be challenging, it is hoped that the insights provided by this study and subsequent research will provide the foundation for better targeted maternal health promotion programs in the study areas, thereby facilitating the achievement of maternal health sustainable development goals.

## Supporting information

S1 Data set Qualitative data. (DOCX)

S2 Data set Quantitative data. (SAV)
