# Supplementary material for: Maternal health care initiatives: Causes of morbidities and mortalities in two rural districts of Upper West Region, Ghana
Source: PLoS One. 2017 Aug 30;12(8):e0183644. doi: 10.1371/journal.pone.0183644 (PMC5576685; doi:10.1371/journal.pone.0183644)
Supplement: S1 Data set — (DOCX) [file pone.0183644.s001.docx]

**Other nurses: DBI DON**

***I realised from the survey, you don’t have an ambulance for referrals***

Yes, we don’t have, but we do have a pick-up

**Aside the pick-up, do you have other arrangements for emergency referrals of pregnant women and babies?**

Yes! The District Director’s official pick-up is sometimes used during emergencies. There is also an initiative called “community emergency transport system (CETS)” whereby all communities have been educated on the need for such transport systems. In fact, it’s very perfect at Woggu community. They have contributed to purchase an ambulance van for the community.

**Is CETS a community initiative or government?**

It is a collaborative initiative; but I can also it is government because we brought about the idea.

I also discovered from the survey, some expectant mothers are unable to commence antenatal care early because of the distance and the NHIS Card renewal centre. What efforts have you made to decentralise the office to Issa?

We have a team of the NHIS registration and renewal staff in Issa now. Nadowli have been detached from Issa. They occupy part of the Health Directorate at Issa. Jimpensi, Kenkelley and all those communities are some of the people who do not subscribe to NHIS.

**Some pregnant women do not commence antenatal early; what is your view on this?**

In fact, it is multifactorial why pregnant women do not patronise ANC services. some are socio- cultural, that if people know about the pregnancy it will miscarry and the long distances. Other women feel shy to carry the card because of stigma of the public knowing she has conceived. Hence, the midwives are often compelled to keep the card with them so that people do not see them holding the card, because of stigma type of thing. The card is kept at the facility for until such a time when she can no longer hide the pregnancy, then the midwife will release the card for her to keep with her wherever she goes. It is one way of addressing that, since the card has a picture of a pregnant woman. It is one way we have adopted to overcome the myth. With education, the numbers are increasing compared to the past.

**Others complain of distance to laboratory services**

Yes, Issa in the past arranged with Bill Laboratories to collect samples to Wa and then get the results back.

**What are the challenges faced in providing maternal and newborn healthcare in your district?**

The district has five health centres and twelve CHPS compounds. However, we currently have 7 midwives at post which is inadequate to provide maternal healthcare to the large number of pregnancy issues we face each day. We ought to have midwives in Owlo, Jolinyiri, and Tabiase, but we don’t have any more midwives to post to those health facilities. The midwife situation is actually a big challenge. Even the district capital, Issa needs more than one midwife; but we forced to make do with just one. Previously, skilled delivery meant, a birth attended to by a midwife or a doctor. How about the CHNs we put at the CHPS compound and ask them. When a woman is delivering, they should catch. Therefore, I think any delivery that is supervised by a trained health worker should be considered skilled delivery. Therefore, the CHNs are forced to always refer to facilities with midwives, and considering the distance, we record poor outcomes or homebirths. Will they go? Water is a necessity of life. Women come to deliver and must be washed meanwhile, there are no water facilities in the health facilities. Some expectant mothers get discouraged from giving birth at the health facilities. Also, the distances to the health facilities without vehicles linking them, how will they go to the facility and after childbirth, how will they go back home?

For the equipment, DANIDA has supported some CHPS compounds, so it is not much of a problem now.

***I realised, the ANC women support in drawing water for the nurses (midwives, CHNs, CHOs) which is a burden on the community***

Yes, where they see it as a burden they don’t attempt to do it. We have asked the community to schedule themselves into sections, and provide water on rotational bases. I understand it is burden, on some communities, particularly those with persistent water challenges.

**Suggestions**

We need to continually provide education to the mothers. Expectant mothers will commence ANC early for assessment. This will prevent malaria in pregnancy and other risks associated with pregnancies. Pregnant women and family’s needs education on BPCR, particularly the primes who have not had childbirth experience for them to receive intensive care for safe pregnancy and childbirth. We are extending our education to get men involved in ANC and ANC classes. We need skilled deliveries at our facilities, instead of the elderly women. TBAs who want to continue also need further education so they can work in collaboration with the midwife during childbirth. This can promote domiciliary childbirths in the presence of the midwife and conducted by the TBA. The credit, if the TBA wants she can even take, but once, it is done under the supervision of the midwife, we will be sure the mother and baby are safe.

The district assembly should sponsor the training of midwives, now we realise there many midwifery training schools that are in place in the upper west region. The community health nurses now, staff attrition is reduced now to the barest minimum, nurses want to join their husbands in other regions. But when you were a community health nurse, and when they had not become a nurse, where was that little young boy. I have some transfer cases. At least, they should serve for the mandatory three years before seeking to go on transfer to join their husband. So at least if the Assembly can support us to train more midwives so risks with pregnant women will be reduced. We encourage all ANC women to use the CHPS compound as their first line of contact.

We also need means of transport for emergency referrals of pregnancy cases. The assembly has promised buying three (tricycles) for the district for emergency referrals. The Member of Parliament has replaced the engine for the pick-up car at Daffiama health centre. It’s a fairly used engine. The community health nurses are doing well, because, they use their personal motorbikes to support service delivery. It is the reason I have been saying, government could provide all facilities, with durable motorbikes, some could carry out the referral by themselves. We encourage the nurses to help pregnant women reach the referral facility, which is a challenge. Daffiama health centre has arrangements to secure laboratory services for the district. Issa as the district hospital also deserve a hospital. The late regional director of health services for Upper West was in touch with the government of Israel to get Issa a hospital. the arrangements was not complete before he died.

**Duang Opinion leaders**

**Problems facing maternal and neonatal healthcare delivery?**

One main issue facing Duang community is the status of our health facility. It is still a CHPS zone. As a result, most MN healthcare cases are referred to Issa HC and subsequently to Nadowli hospital, but we don’t have any transport system available to pick patients. Even a tricycle is not available here to pick pregnant women on referral to Issa HC or Nadowli Hospital during childbirth or when sick other than childbirth. The clinic does not also have most essential medicines. Hence, many maternal and newborn health cases are referred to Issa Health Centre.

Duang CHPS zone do not have a midwife to conduct childbirths. Aside emergency childbirths, once the expectant mother is assessed and realise she can sit on a motorbike to Issa HC, the CHNs at the facility quickly refer the mother.

In recent past, measles outbreak led to the loss of many lives of children under five years of age in the community. However, the establishment of the CHPS zone, which routinely carry out immunisations, the death toll has reduced among children.

We also recorded few cases of maternal deaths in recent pas; about two years ago, but we only had one case in 2015.

**What was the cause of the death of the expectant mother you talked about?**

She was in prolonged labour at Issa HC and was referred to Nadowli hospital. On their way, she passed on before getting to the hospital.

**What work do expectant mothers do at farms?**

When women conceive, we don’t allow them do hard farm labour again. When it rains, they go to sow seeds because, those little activities keep them fit.

**What of the fire wood, who cut and convey it home for them to use?**

Carrying headload as expectant mother when she is due to give birth is not allowed these days by many families.

Nursing mothers are not also allowed to be involved in hard work until after 6 months after her childbirth. Both expectant mothers are allowed to choose farm labour that matches her ability.

With the issue of firewood, it’s part of the little exercises which keep them active and healthy. Some men support their wives with the cutting but it’s the duty of the woman whether pregnant or not to convey all home.

Unlike the olden days, at present some expectant and nursing mothers may be exempted from family farm labour due to her condition of health, but she might indulge in her private farm work and even worse, do charcoal burning.

Other expectant mothers are willing to risk into menial jobs to achieve their personal gain.

Usually, we sympathise with them for their feminine role, but we would find them undertaking their private jobs of pito brewing, charcoal burning or even their own private farm for separate income. Other mothers log wood for money. They do these jobs when the husband is gone to farm already, she will sneak out into her own desired income generating activity.

My wife was a nursing mother of less 4 months postpartum and I did exclude from farm work but she will felt healthier enough to carry the newborn into her own farm though I thought she was at home.

W**hat is your perception about women who refuse to take up ANC?**

During ANC, we normally exempt them from farm work so they could receive the necessary maternal healthcare. Sometimes, we return from farm to realise she failed to go for ANC but instead engage herself in her personal income generating activity, to visiting the clinic.

**Do women ask you to accompany them to ANC/PNC?**

Yes, they do. However, because we are farmers, we don’t usually have time to accompany them during antenatal and postnatal. Taking them to ANC is not something we are familiar with. The clinic is only a year old in Duang community, hence expectant mothers used to foot to Issa health center for maternal healthcare. Some men do not own motorbikes and therefore, to get up and foot with our wives to Issa HC was inappropriate and time-wasting.

**Why do you say it’s time-wasting?**

It is time-wasting because, they spend a whole sometimes seeking ANC.

I’m a health volunteer, yet I don’t also accompany my wife to seek maternal healthcare. But with the construction of the CHPS zone in the community, during lean season, some youth do accompany their wives to receive maternal healthcare at the CHPS compound.

**Why do expectant mothers’ refuse to take oral medications of the given at ANC?**

It’s two years now since I had a misunderstanding with an expectant mother who refused to take in the medications. I told made to know the medicines are usually purchased by government at high cot and supplied to the clinics freely for improved pregnant and birth outcomes. However, if you know you will receive it but not take it, you ought not to have accepted it, because it’s sometimes in short supply and she was wasting it.

**I learnt from some discussions, expectant mothers throw ANC medicines away?**

Yes, it’s true. I once reported an expectant mother like that to the nurses. She took it and said was giving her nauseating smell and therefore refused to take it. My wife has done that too before. Hence, one day when she went up to the clinic, I followed-up and gave the report to the CHN although it led to some misunderstanding between her and I starting from the clinic.

**BPCR means?**

When she is due to give birth, you the husband will take her to the clinic to deliver safely. Now having a childbirth at the clinic is covered by the NHIS, and therefore it’s absolutely free of charge.

As part of BPCR, expectant mothers must also attend ANC regularly. We have formed a committee on community emergency transport system (CETS) spearheaded by the CHO for all healthcare seeking including pregnant women, to ensure timely access to relevant care at Issa HC or Nadowli hospital.

We have discovered many referrals of expectant mothers to Issa HC were almost always further referred to Nadowli hospital, but when the woman and her family return to Duang to prepare financially and to get other items, the women end up giving birth at home.

I gave birth in one instance like that because of sympathy on my husband. He had no motorbike and money. Therefore, when we were referred to go to Nadowli hospital, I realised he was going to borrow more for us to go, so I pushed harder to safe my life and the baby.

**With the home births, how do you get vaccinated?**

They would insult us before administering the vaccine on us.

**Responsibilities of husband when women are pregnant?**

It’s our responsibility to save money towards her birth and healthcare seeking. I don’t do anything because my wife has been unfaithful to me. So whenever she conceives, I expect her to go to the second partner for the support she needs.

**Cases from health volunteer at Duang**

1. An expectant mother ones went to the farm and to cut firewood. After she had cut them, made efforts to carry it up her head and fell down with it. A hunter spotted her struggling to convey the wood on headload. He moved to where she was and interrogated her on why she was due to give birth but indulged in this risky activity? He supported her carry the wood home. The next day, she had a stillbirth to twins, and both were boys. The expectant mother was a prime. One came out at duang dead whilst the second was felt kicking but there was no means to rushed her to Issa HC. When the family finally got her to Nadowli Hospital, he also died. The mother suffered some complications but was able to conceive subsequently.
2. I believe God has joined us together to support each other, but bad peers deceived some expectant mothers to terminate pregnancies illegally because I’m poor and unable to fend for her. In the process, she lost her life. It’s not only mine, other expectant mothers have done illegal abortions here in Duang which leave them in perpetual trauma for life.

**Can I have an interaction with one of such women?**

It’s not allowed. She will fight with me after you are gone. So let’s don’t attempt it.

**Suggestions for improved MNH care in Duang community**

All the truth dwell with the expectant mothers, because what we ask them not to do when pregnant or nursing such as farming, they do it secretly for their private and personal gain.

It is not allowed and recommended for expectant and nursing mothers in the community to indulge in charcoal burning. We belief it creates intense heat which might definitely lead to poor birth outcomes. Expectant mothers should ensure they register early for ANC and complete all the recommended number of attendance. In such case, nurses will detect any abnormality and risks and provide early treatment. These will ensure safe delivery to a healthy baby as well. Nursing mothers should also take full PNC courses for the healthy growth of the baby.

OTHER NURSES: ONE

I understand there is no ambulance in the entire District (Daffiama/Bussie/Issa), apart from the pick-up car at Daffiama Health Centre (HC), hence what arrangements are in place for pregnancy, newborns and childbirth emergency referrals?

We have something we call CETS – community emergency transport system. Every CHPS compound try to organise the community, then they will start putting down some amounts of money towards buying a vehicle. So the community opens an account and begin to save towards the van. But as at now, in case there is a referral case, the client looks for their means, and it’s either motorking (tricycle motorbike) or we call the pick-up car from Daffiama HC but the client and the family bear the cost of the referral. We only help them by calling.

**On the average from Woggu to Nadowli, how much do they pay for the pick-up?**

It’s 100 Ghana cedis. But this fee was for 2015, for now I don’t know. It’s a long time we called the driver to refer a case because it broke down for four months from January to April, 2016.

**In 2016, have you had some referrals of expectant mothers?**

Yes, we have had referrals, but they come with their own means- motorbike or tricycle. It’s the situation where they find it difficult to get means then we call the car for them.

**What are the outcomes of births from the referrals by the tricycle bikes?**

Sometimes, due to over-shaking on the rough road, many of them give birth on the road.

**Do they have livebirths or stillbirths?**

Both. Many of them are usually stillbirths. As the child comes out, the child becomes asphyxiated and no nurse is there to resuscitate the child. It’s now that JICA and MoH have put in place a safe motherhood protocol. For now, if you refer any expectant mother, you the midwife have to go along. It’s still the same story. Some people come with motorbike or tricycle and they go along side with two other relatives of the expectant mother. Sometimes, you the nurse find it difficult to get means so you can go along with the client and the relatives. Even when get means to the referral point, bow to return to your facility become a big problem.

**Do you also join the tricycle during referrals?**

Yes, we are supposed to be in the same means of transport to protect the child until we reach Nadowli Hospital. However, the problem is, when we join them there, we don’t get means to come back to our health facility. The relatives stay there until the client is discharged. It’s difficult for us but we are forced to do it and we are doing it too. And if there is bleeding situation or you think the situation is too critical, we don’t leave the woman alone. Mostly, expectant mothers deliver on the way. Some have live births others also have stillbirths.

**ANC no, the acceptor rate has improved. But in some places it’s still not, what do you think is the cause of the refusal by pregnant women to take-up ANC or complete the course?**

For my community, what I have seen is, those who do not report for ANC, when I spot them, I call them. Some of them are old to conceive again, so they feel shy to come out for people to see them.

**But have they reached menopause?**

No, they can give birth, but they have given birth to over 12 or more children and still get pregnant again. So it’s a shame to them, hence they find it difficult coming out. Some of them too are pregnant but not by their husbands so they try aborting and it didn’t work. So they don’t come for ANC. Unless I see them and counsel them, then they will come. A whole lot of things are happening in this community. Some them too are school children who are underage who get pregnant. They try illegal abortions when it’s not terminated, they hide it until labour set in, which time they then come to the clinic.

**Don’t the school girls receive ANC?**

Some of them do come for antenatal. But it’s a rare situation here. Since I was posted to the Woggu, for the past two years now, it’s only one case I had like that of a school child not receiving ANC until she went into labour. So, I asked the women and was told she was always in the room. Even people living around didn’t know about her pregnancy and her condition of health. It was when she began losing liquor and in excruciating pain, the family then brought her to the clinic.

**Was the outcome of her childbirth successful?**

Yes, she gave birth successfully here.

**I remember you mentioned this teenage pregnancy during the interviews with expectant mothers at the facility level. What has been done to control school girls and under-age children getting pregnant?**

Well, we are having series of durbars talking about the menace of these acts. We have also been giving them health education. Few days ago, we had another durbar in Woggu, in partnership with a non-governmental organisation who is helping to stop it. We are talking to the community people and the girls. We give them pieces of advice particularly asking the parents to encourage young girls to come for family planning devices to prevent these pregnancies and infections.

**Do you think the integrity of pregnant women is compromised as you carry out duty of care to clients?**

No, I don’t think so. They are fine with the treatment and service offered them. Since I came to the community, I have not had any problem with any client or witness any of the expectant mothers being treated badly by any nurse.

**But have you heard about issues of such anywhere by other nurses?**

Yes, the complains are there everywhere and we have been hearing about them. we hear them every day but you wouldn’t know is doing them and no is willing to expose such a nurse. But we do hear them!

**Challenges in ANC, labour and postnatal?**

The resources are not there.

**What resources?**

For instance, I’m in this village, but we don’t have oxytocin, most of the infusions are not there.

Infusions like what?

Normal saline and ringers lactate, oxytocin. We don’t have even a little bit of these to conduct deliveries. So these are some of the issues that are very discouraging. As I’m sitting down, if there is a labour case and the woman is bleeding, there is nothing I can do to resuscitate the woman. I will just watch her dying and will have to write a report on the dead case. It’s always scary. The absence of mandatory resources make some midwives leave the facility. Because, if I’m not there and there is a case, the nurse around will quickly refer it. It makes the work difficult, scary and stressful.

**What other?**

The means of transport as in referrals system. There are child birth cases that come and everything seem to be normal with her, but all of a sudden, the condition of health changes, which you have to refer to Nadowli hospital. Immediately if there is means, a doctor comes in and everything is normalised for the expectant mother. But with the referral, the family will look for means, even when you call the driver and he is somewhere, there is no fuel, the car, is not working, among other reasons these trotro drivers give and by the time the family get means it’s too late to have positive childbirth outcomes. Sometimes, they get there and the baby is either dead, very asphyxiated or the mother is even anaemic or something else.

So the community itself we are handling belief so much in tradition, such that when we give health education, they don’t really pick it. Many of them don’t really accept the education given. However, we have not given up on them, we are devising strategies and working on them. with new ways of education, the beliefs are going down a bit, leaving a small percentage who are unwilling to give up the beliefs.

We don’t also have a site for placental disposal. So indirectly, they are carrying infections to their various houses. We are here to bring good health, but in a way, we are transferring infections. We just need a placental pit.

**Do you apply anything onto the placenta before giving it out to them?**

Yes, we put in chloroquine (0.5 percent) before giving it out and we believe it kills 99.9percent of infections. However, it’s not good to give the placenta out. Because, as they are taking it home we don’t know what they do to it. They might not burry it instantly or could pass on to so many hands before it’s finally buried.

**What suggestions will you make for improved MNH care in your community?**

I will suggest especially with the resources. Government should not only pass on protocols but resources for us to work with. We need them to make work easier for us.

**Who is responsible for providing the resources? the district or ministry?**

Both. Because, anytime we talk, the district tells us it’s the ministry who should provide them. wherever, they are going to get logistics for us to work with, we plead with them to provide them, so working will be easier. When you have everything, you happy to attend to cases. When we don’t have, improvising is always difficult. If they can also be a way of getting funds for our placental site and transport. For the transport, we are working towards it.

**I learnt the community is about to buy an emergency referral transport ambulance?**

It’s true. Some of the community members down south brought some pictures of ambulance

But the community people are not fulfilling their part of the financial agreement for it to arrive. Their sons and daughters who work in the southern part of Ghana do come home every December to have a get-together. They have agreed to purchase the ambulance, but they also want those at home who are the direct beneficiaries to also contribute so they can keep that for maintenance. They contribution will also commit them to use it wisely. So now, it’s their portion which is delaying the emergency transport system.

DUANG NON-PREGNANT WOMEN

**What are the causes of maternal deaths in your community?**

We have had maternal deaths, but it’s about a year we have not recorded deaths. Pregnant women who died was a result of non-availability of maternal healthcare. Other women, deliberately refused to go to Issa for antenatal care until childbirth. So during, labour supervised by the TBAs, some die. But because it’s not always in the hospital we don’t know the cause.

**What were the causes of neonatal deaths in Duang Area?**

They are a result of the refusal of expectant mothers and nursing mothers to ANC and Postnatal care (PNC). We understand, when pregnant women receive ANC and there is a risk with the conception or breech presentation, the nurses will immediately treat for safe pregnancy outcomes. However, there are some women who do not seek ANC until child birth. They are the ones who normally have stillbirths. Other pregnant women will attend alright, but they will not take the medications.

**What work do pregnant women do in your community?**

***At home?***

Expectant mothers in this community partake in all kinds of work at home. They are the ones who cook, brew pito, draw water from borehole, give the kids a bath, washing of clothes of the entire family and still cook food and carry on headload for farm labour.

***At farm?***

It is women who sow seeds at farm, transplanting of millet seedlings, cutting and carrying of firewood, harvesting and charcoal burning. In fact, our tribe is the hub of charcoal burning in Upper West Region. So we engage in charcoal business though not as much as non-pregnant women, alongside farming activities.

**Safe pregnancy?**

For pregnancy to be safe, we must seek and patronise maternal healthcare. Eating of fruits such as dawadawa, mango, shea fruits, among others. We are unable to acquire the fruits and food items listed in the ANC card, however, we eat what is available.

**BPCR?**

it means the person is talking about buying items for the birth kit. The items usually include; soap, cloths, baby clothes, rubbers, blade, thread, and Dettol. We are normally asked to carry them everywhere we go when in our last month of gestation. But these items do scare some women from giving birth at the clinic because they are very expensive for everyone to afford. When, expectant mother does not have them and go to give birth at the facility, you are insulted and embarrassed by the nurses. therefore, when pregnant women are unable to buy them, they stay at home and have their childbirth.

**Community initiatives for emergency referral of pregnant women?**

We don’t have any community initiative. However, during emergency referrals, the client’s family can borrow from either the men group VSLA or the women group VSLA box.

**Other groupings where MNH issues are discussed?**

We have VSLA meetings among men and women groups ones every week. During those meetings, the CHN do come around to inform us of upcoming national immunisation days. But MNH care issues are not discussed at group meetings.

**Homebirths?**

Expectant mothers do give birth at home. It is very common here. Others also give birth on referral to Issa health centre or Nadowli hospital. anyway, when women deliver outside the clinic, we convey them to the clinic for immunisations, although all women make efforts to give birth at the clinic.

**Suggestions**

We don’t have a midwife to conduct deliveries. If there was a midwife, the wayside births during referrals to Issa health centre would have always been avoided. Government should post a midwife to the community. We receive routine ANC at the CHPS compound, but occasionally, we go to Issa or Nadowli when there are complications. Considering the distance to Nadowli coupled with poor road surface condition, it further worsens the complications. Therefore, if Issa HC could be upgraded to provide many of the relevant healthcare services such as comprehensive obstetric care and laboratory investigations, it will improve upon our healthcare.

We also need an ambulance for emergency referrals. It is motorbike we secure during referrals to the next level of care because of the absence of trotro linking us to Nadowli. If there was ambulance van, we would only ensure we save and hire that for emergency referrals.

BUSSIE OPINION LEADERS

**What work do expectant mothers do in your community?**

When they are pregnant, we encourage them not do strenuous activities. We also exempt them from farm labour and other jobs that would affect their health. We are aware we are very far from hospital, to receive relevant care in case of complications. For instance, carrying of water on headload over long distances and conveying firewood too. Expectant mothers and women in general have some lukewarm attitude toward seeking maternal healthcare. Some of them refuse to take up antenatal until their partners coerce them. however, when she is in complications, the husband is involved to support her to health facility. We often do exempt them from farm labour and other family work, but some sneak into charcoal burning and pito brewing leading to so many difficulties when giving birth. Other even get miscarriages from the heat of the fires. Because Bussie market is the largest commercial centre in the entire district, the women capitalise on the customer availability to brew a lot of pito and also burn charcoal to earn income. Some expectant mothers are often advised by the midwife to keep off any heavy load, however, they insist on brewing pito, cutting and carrying firewood, drawing of water on headload, among other menial activities with the corresponding effects on the outcomes of the conception.

I have a brief issue bordering me. For instance, my wife once secretly went for FP services without my consent. When I discovered it, I forced her to go and have it removed from the arm. She conceived after removing it and refused to go for ANC. Meanwhile, I picked an issue with the nurses at the clinic before they actually removed the device from her arm, so knowing they could be side effects of the FP device on the pregnancy outcomes, she ought to have been regular to antenatal. I still have the removed FP device in my room.

**I wish to clarify this, don’t your wives involve you of their decision to receive family planning?**

Yes, some hide from us. My wife did not inform before taking it in, which was the reason I propelled the nurses to have it stopped.

**Maternal deaths?**

Yes, in 2013, we had a case of maternal death here.

***Stillbirths?***

Sometimes when they get to the clinic and are referred to Nadowli hospital. We suffer in getting means to transport the expectant mother. So by the time we get motorbike, it ends up in still birth. Other times, they give birth on the road to the referral facility.

We have frequent referrals here. But many of these happen when the midwife is absent. The other nurses refuse to manage the progress of the labour, however, they often give immediate referral without even carrying out initial assessment of her progress. Hence, the shaking on the corrugated road and potholes is one major cause of the stillbirths. The women have some negative attitude. Oftentimes, when men suspect they are due and probably inquiring, they refuse to declare the progress of the pregnancy. So when the husband takes off to farm or elsewhere, expectant mother will then begin to look around for motorist to take her to clinic. Such delays lead to childbirths on the wayside. The clinic is within the community, but we have had so many wayside births.

I also observed that, some pregnant women in our area do not take in medication given at antenatal. In December 18, 2015, my wife did a similar thing. She was supposed to give birth on the 5^th^, but when I took her to the clinic, we were asked to go back home. On the 18^th^ of December, 2015, i took her to the clinic again and was asked to go back home for her NHIS card. As I searched for it in her bag, I found several sachets of the ANC medicines in her bag. She was not taking the medication. I will suggest every male here to intensify his monitoring on the intake of these essential medicines.

We need an ambulance at the clinic. When we get there and are referred, ew can easily secure that to send the expectant mother to the referral facility.

**If someone talked to you about birth preparedness and being ready for complications in pregnancy, what do you think they would be talking about?**

When they begin ANC, the assessment is done every month. However, when it’s reduced to bi-monthly or weekly, then the couple have to get soap, blade, cloths, covering clothes, thread, hence, after the childbirth, they can use them to clean and rap the baby- birth kits.

***Local oxytocin intake?***

The women still administer them.

***Traditional beliefs?***

When child is born at the clinic, the placenta is buried there. Customarily, in Bussie traditional area, it’s wrong to bury the placenta in a different community or at the clinic. It should be buried around the family surroundings.

***When it’s buried at the outlet of the bathhouse or within the house, what is the essence of such belief?***

It’s our tradition. Even today, people who are in southern part of Ghana do carry the palcenta to Bussie to burry. When a baby boy’s placenta is buried outside the community, the child cannot inherit a chieftaincy title or be a traditional head in future. Also, if it’s buried in Bussie clinic, it’s still acceptable, but when they are referred to Nadowli hospital. It should be taken to Bussie for burial. They swap the destiny of the child by burring them in the placental pit mixed with others’.

***Do women patronise ANC or not?***

There are some women who receive whilst others do not receive maternal healthcare. Some women also commence ANC very late sometimes in fifth or sixth month.

***Why late or not at all?***

Some women do not want the public to know about their pregnancy so early.

**What do you suggest are the roles of men in pregnancy?**

As for me, as soon as I know she has conceived, I take her to the clinic to register for ANC. I do this to prevent complications and poor pregnancy outcomes.

Case Bussie opl:

One day, we were farming near the road to the clinic, when a man with the wife to on their motorbike to the clinic for her to givebirth; collided with another motorist. So the bike was on top of the pregnant woman then we rushed to their aid. As soon as I picked the bike from her, the child began to come. So I called on women around to attend the birth. She gave birth at the accident spot. The husband rushed to the clinic and picked up the midwife who came and took the child and mother to the clinic for further assessment and immunisation. The absence of a van or ambulance, is a cause of the delay in reaching the facility.

Those of us who live and farm at kamahegu encourage our pregnant wives who are due to move to Bussie so they could be close to the midwife and main road.

Some women are unfaithful to their husbands, particularly those of us who are aged. Therefore, when they are pregnant, the men she their responsibility. It’s the responsibility of men to assist in fetching household water, cooking and all other jobs that could affect her health. Some of us do these jobs.

BUSSIE - NON-PREGNANT WOMEN

**What do you think are the main causes of deaths of newborns in this community/district?**

When we conceive for one or two months, we ought to receive maternal healthcare. If we fail to take up ANC early, the midwife might not detect risks early for treatment which do lead to stillbirths or death of the mother or both.

Also, if we indulge in hard work such as lifting of heavy load can affect the foetus. A lot of mothers here carry heavy sticks on headload, brew pito, sow seeds at farm and even do weeding at farm whilst pregnant.

Receiving early ANC and counselling are helpful for pregnancy outcomes but so many expectant mothers do not patronise it.

Maternal deaths and still births result from sicknesses in pregnancy. Expectant mothers who do not receive ANC early stand a high risk of sicknesses, mostly malaria and sexually transmitted infections with the associated effects on pregnancy outcomes.

In terms of miscarriages, receiving maternal healthcare early could give them appropriate counselling to prevent future ones.

***Stillbirths or maternal deaths?***

Stillbirths and maternal deaths could also come from petty quarrels at home. These could cause depression in the mother causing poor progress of conception. Some mothers attempt terminating the pregnancies due to the quarrels which oftentimes lead to stillbirths or loss of both lives. We have had an instance of maternal death like that two years ago. She was even beaten by the husband which led to complications and was referred to Wa and she died there. We were told she had so much internal bleeding, but because she could reach the facility early, they were unable to treat it.

We are usually admonished to receive antenatal care early so the Iron (III) acid, tetanus injections, tests and scans will be done early to prevent any future risks of complications

Maternal deaths are caused by the petty quarrels. Retained placenta is also another prominent cause of some deaths in our community. The retained placenta are common among the homebirths. Usually, when we are encouraged to give birth at the health facility supervised by a midwife, one obvious reason many uninformed expectant mothers give is; “our previous homebirth was smooth therefore the next will also be successful.” Instances of this sort, when she is delayed in reaching the facility, she bleeds excessively by the time the reach the health facility.

Another cause is trapped placenta. When it’s home births, over-pulling of the placenta leaves a thin lining in the uterus. Failure to remove it or if it is unnoticed could lead to sudden death of the mother many days after successful childbirth. This thin lining is usually at the tip of the placenta.

The reason we discourage homebirths nowadays, is with the cutting of the umbilical cord and removal of the placenta. The placenta also contains some fluid which should not excrete into the uterus. Therefore, if proper care is not taken during homebirths, these could be released into the mother causing illness that may lead to loss of her life. What I will say is that, “homebirth kills’,

**What activities (cultural, social, economic) should pregnant women not do in your community?**

We go to farm and the bush to cut and convey firewood on headload whilst pregnant. We do the cooking of food at home, brewing of pito is our dominant income making business and we still do it even if your due to give birth. We maintain all jobs we do whether pregnant or not pregnant. We know the risks such as complications, miscarriages and loss of lives sometimes. As for miscarriages, we know we will get pregnant in few months’ time again.

Expectant mothers do climb trees to cut firewood or harvest “dawadawa” fruits. These could result to pressure from the husband as being lazy. So in the depressed state of mind, expectant mothers do all kinds of unusual activities even if we could die and rest. These often lead to complications and loss of pregnancy or both.

**Do you experience quarrels at home when pregnant?**

Yes, a lot.

**What cause the misunderstanding or fights?**

It’s mostly because we are unable to participate in the farm labour in the scorching sun as before. We do not risk in the scorching sunshine to do farm labour because of the anticipated risks associated. Meanwhile, other expectant mothers still do all kinds of farm jobs in a bit to please their wicked husbands. Refusal to please them is what often lead to the misunderstanding ignited by the man.

Hospital births has huge benefits. We have children who are numbed because the old ladies who supervised the birth of those children couldn’t detach their tongues. However, nurses are trained who conduct complete assessment of the baby to ensure all is well with the parts before discharging them.

**Are there expectant mothers who deliberately insist on having homebirths?**

Yes. One time, my daughter-in-law who was due to give birth travelled home from southern Ghana. I noticed one morning she was not out to do cleaning of the compound. So I thought it was delay in rising up. I checked at her room to find the newborn lying face down whilst she was also lying separately somewhere. I went for cold water and poured it on the baby until it was resuscitated. Some refuse to givebirth at the clinic because they belief, when labour is announced prematurely, it prolongs. So they keep it indoors until they give birth. Some shout for assistance when the baby is almost out. It is an unnecessary pride and ignorance of some expectant mothers, particularly the prime to show their prowess and maturity. We keep telling them, water infusions and blood transfusions cannot be done during homebirths, and even if they become pale, unless at Nadowli hospital, the clinic do not conduct blood transfusion.

**What are the support family give to pregnant women and in the early postpartum period?**

Families don’t support us in anyway when pregnant. We hustle to get soap, care of the newborn and food items. We fend completely for ourselves and the child. I got conceived and went to the clinic and was referred to go for laboratory investigations. I went home and informed my husband of the referral and was told he would not be able to send me to the hospital. I had to look for me means all by myself and went for the laboratory tests, even when my NHIS card was expired at the time. I had to sleep over on the hospital veranda to get my NHIS card renewed before I could do the laboratory test the next day. I even ended up in stillbirth to that pregnancy leaving me in perpetual depression up to date. I suffered so much in vain. I was forced to engaged in all kinds of menial jobs to care for myself. My husband never even asked of my health condition or the progress of the pregnancy.

In Bussie community, the moment a woman conceives, the man behaves as if he is not responsible for the conception. Starting from the laboratory investigations until childbirth, we fend for ourselves in everything. They don’t even support us to go to Nadowli hospital for laboratory tests and scans. During farm work, when we are in pains, we are expected and some men insists on us to continue to work. When we complain, we are tagged as being lazy or pretentious. It is the reason why some women secretly go for family planning pills without our husbands’ consent. However, when they realise we are FP, they follow-up to warn the nurses of such practice, saying “it’s family planning and not women planning”.

We those who have stopped childbirth suffered worse treatment than at present. We had no proper clothes to wear as nursing mother, and were maltreated in worse forms. In that situation, but I was forced to give birth up to eleven times.

**Do you administer herbs on expectant mothers and newborns in your community?**

Yes, we do.

**Herbs for expectant mothers and neonates?**

There are elderly women in the community who treat neonates with herbal syrup. “I had my sick newborn and took round all forms of healthcare with no improvement in the baby’s condition, therefore, one day, I plucked some leaves and boiled them into syrup and administered it on the baby and the baby got healed, so each time, a neonate shows similar signs of poor health, they bring it to me for treatment with the same herbal mixture and some get well. This continued up till date with the herbal treatment. I give treatment to kids with deep-dark veins and pale face. I also burn the sticks of the same plant into coal, grind it and mix with shea oil for the mother to use that as body pomade whilst the leaves are boiled into syrup and given orally. Usually, the paracetamol and antimalarial medicines given at the clinic are unable to treat such sickness”

We also patronise TBA care here. However, we combine both clinic and TBA maternal healthcare services. “I once went for the TBA care before going to the clinic to give birth as I was due on the same day. That labour did not keep long unlike others”.

**What’s your perception about TBA care?**

To me, it’s good. I had pains like labour had set in, but the midwife could not tell me my state of health after each day’s evaluation. Some elderly women encouraged me to seek from the TBA. On arrival, she assessed me and told me I was due to give birth. I went straight home for my birth kit and dashed to the clinic. I gave birth successfully the same evening at the clinic.

**Does the TBA work with the clinic midwife?**

No, she operates independently.

**Does she wear rubber hand gloves when assessing expectant mothers?**

No, she doesn’t wear gloves. She doesn’t have a bench like the clinic too. When assessing, we are made to stand up straight. She sometimes turns the foetus very hard such that, you can collapse instantly if you don’t take care.

**BPCR means ……………**

when we are due to give birth, we wash our clothes and rags, buy some soap and washing powder, blade, thread, parazone, rubber bags and other essential items that will be needed during labour and birth and put them into a portable hand bag or basket. During ANC lessons, we are told to carry along these items so that even during miscarriage or bleeding at any point in time, they could be used to control it.

**Are there community initiatives for pregnancy emergency referrals?**

There are no initiatives in Bussie to help in referrals. Anytime an expectant mother or newborn is referred to Nadowli hospital, it’s the mother who find her means to the hospital. If your husband is around, he might send you with his motorbike or look for tricycle to send you to the hospital. When my granddaughter conceived and fell sick, the man send her off his home. I supported her financially to hire means to Nadowli hospital. When we arrived, a catheter was put on her for passage of her urine, but the partner never even turned up to support pay the bills or the transport cost. The said man even works at Bussie clinic. Other times, our husbands will send us to the referral facility, but refuse to pay the hospital bills.

**Do the men accompany you to antenatal and postnatal?**

Not all. We usually inform them of the ANC but they don’t go. Some say, we are not attractive to walk with them to the facility. We continue to give birth for them because we know the children may be of benefit to us when they grow up. We don’t really enjoy the marriages, just that, it’s same everywhere.

**Do you belief and practice “pregnancy announcement”?**

Yes, we do for first pregnancies of duly married primes.

**At what age of gestation do you conduct it?**

We do it anytime we discover she conceived. Otherwise, when it is discussed by the public to her hearing, she will miscarry. The practice commits it to the ancestors who prevent it from bad omens from strange spirits possession.

**But I notice girls have pregnancies outside marriages or before marriage. Why do they stay?**

They are different from married women. The customary marriage rite commits her to the ancestors, hence when we fail to do the pregnancy rites, they will not protect it which could affect it leading to miscarriage. However, the rites is so common these as in the past, because most first conceptions at present occur before she is customarily married. Therefore, we don’t perform the rites on such pregnancies.

**Do you carry out any adolescent sexual reproductive health practice, such as FGM in your community?**

no, we have been stopped from genital mutilation. This ban is the cause of the rampant teenage pregnancies among the young and school girls. Teenage pregnancies are huge burden on us the parents.

**Do you have other platforms where maternal and neonatal healthcare issues are discussed?**

No, we have never discussed or received any education on MNH care. We do have women “Susu” savings groups, but no one has ever spoken to us about them.

JIMPENSI/KENKELLEY CHPS (FACILITY HEAD)

**What issues are faced with in your providing maternal and neonatal healthcare to your clients?**

In this community, we know we have so many challenges with maternal and neonatal health outcomes, particularly stillbirths. We don’t have cases of maternal deaths these days, however, stillbirths are still occurring.

**Are there some beliefs and practices that you think are the cause of the stillbirths?**

Yes, it is believed, not all members but some do belief when a woman (prime) conceives, they have to perform some customary rites before everybody is aware. In such cases, prime expectant mothers don’t report early to the facility to commence antenatal care. They keep waiting for the rites until it’s late. They emphasise that, even the nurses must not know until they perform the rites on her. Meanwhile early ANC is very important for the health of the mother and the newborn. It is through early antenatal care we also refer them for laboratory investigations, for early detection of any abnormalities for early treatment or management. However, when expectant mothers report late especially in the second or third trimester and there is any abnormality, it will be difficult to treat it because the harm might have been caused already to her and the unborn baby. This can lead to still birth.

**With regards to staffing in your facility, are they adequate to provide required MNH care to the communities?**

We are not enough to provide the needed MNH care. I’m the only community health nurse for the facility. We ought to be two or three CHNs. The facility serves Jimpensi, Kenkelley and Vietor. The women give birth a lot here. As it stands, there is no midwife in the facility to supervise childbirths. The enrolled nurses are also two instead of three. We ought to be 3CHNs, 3ENs and a Midwife.

**The expectant mothers’ complaint you don’t have a place to store medicines, what is it you don’t have?**

The facility does not have vaccine fridge to store them. There is electricity here now, but we don’t have a vaccine fridge. When we even conduct a childbirth, we have to ride to Kojokpere health center for polio vaccine for the newborn, and return the remaining left to Kojokpere again for storage. When expectant mothers are around 20^th^ week of gestation, we administer tetanus toxoid injection (TTI), but the vaccine cannot be stored here. We have to go to Kojokpere each time to pick it up for use. This means, with some expectant mothers, if we are not careful they might miss those injections we they are unable to come on the same day as others. We do find some pregnant women who have not received the TT vaccines, however, we have only one motorbike for all activities, we are unable to cancel some health programs to travel for the vaccine to administer on expectant mothers who had not received. It is sometimes difficult to go to Kojokpere HC for the vaccine to inject expectant mothers and newborns.

**Do you have enough non-drug consumables for healthcare delivery?**

At the moment we have a very big challenge. Because, when we go to regional medical stores, some essential consumables we use every day are not available.

**I saw you wearing polythene bags or they are improvised hand gloves Ghana Health Services now provide?**

It is one of the challenges we are facing. For instance, hand gloves are very important and mandatory for infection prevention and control, but right now, we don’t have and it’s the reason I used that rubber to conduct HIV/AIDS and Syphilis tests. when they were bringing the consignment on the Rapid Diagnostic Tests (RDT) kits, those gloves like polythenes were included for us to use. We have even had consignments on the RDT which had no gloves included. Hence, we do use bear hands to conduct tests on patients. The improvised hand gloves you saw me wear, were old gloves I found, because we don’t have hand gloves in the entire facility and the current RDT kits were not supplied with gloves. Many of the non-drug consumables are not available. Items such as handwashing soap and hand sanitizer for the prevention of infections and cross-infection control are not currently available for us to use. The brought recent stock of items for the facility but when we looked at the non-drug category, it was NIL for infection control supplies.

**Now that you don’t have a midwife, do supervised childbirths?**

Yes, we supervise emergency childbirths. We only conduct emergency childbirths. If the expectant mother come and upon our examination, we found out, if she is referred to Kojokpere, she could give birth on the way, we monitor her and conduct it. However, if we realise whe would get to the referral facility labour commences, we refer and even accompany her when our single motorbike is available. They carry her on motorbike and we also follow on our bike. We follow-up if we are in doubt of the progress of her labour.

**With the rough surface nature of your roads, does it affect childbirth outcomes?**

Yes, it does affect birth outcomes. We have two trotro buses in the community but due to the high cost of hiring to transport expectant mothers on referral, many of them go by tricycle or motorbikes. We now have CETS in the communities. They have save money one can borrow to transport expectant mothers on referral. However, Kojokpere is not included in the accepted lists of referral centres for one to access the fund. If it is Nadowli hospital or Wa hospital, it will be released to you because those facilities are farther.

**Can’t you negotiate the cost of hiring the buses so it is convenient and affordable to everyone?**

We have had a negotiation session involving the opinion leaders, the chiefs and the community health volunteers and they were able to negotiate it to the current level. It was formally very high. Many of the houses have tricycle motorbikes, so they easily access those during emergencies.

WOGGU – HEALTH FACILITY HEAD

Yes, they belief in early marriages. They just come and catch the girls they feel they want to marry. Forced marriages are rampant here. We have been educating them on it, but elopement still persists. Because of this, teenage pregnancy is very high in Woggu community.

**Are the teenage girls usually at risks of poor pregnancy and childbirth outcomes?**

Yes, it’s school children. Some of them, we hear them wailing and shouting in excruciating pains of complications and there is nothing we can do about it.

**Have you recorded any maternal death resulting from teenage pregnancy?**

Not death as such, but they have birth difficulties. They have difficulties when they are in labour. Many these teenage girls have stillbirths.

**Have you had any maternal deaths not necessarily at your facility which you know about resulting from teenage pregnancy?**

No.

**How about adult women?**

There was a case of maternal death which, I learnt she tried to abort and lost her life on referral to Nadowli Hospital.

**What traditional beliefs and practices on maternal and newborn healthcare are in Woggu community?**

There are practices like herb intake and local oxytocin intake.

*What can you say about them?*

It’s common, especially among women who are due to give birth. When they feel little pain, they take the local oxytocin. Sometimes it’s not yet labour and they take it. When the labour then set in, they go through severe pains, resulting to wailing and crying and for such cases, we just refer them to Nadowli hospital, because for Woggu CHPS there is nothing “I can do to help them”.

**What are the reasons behind late turn up for ANC?**

Sometimes it’s their farm work. They sleep most at times in their farms. When it happens in the farm, until it’s time for them to come back home, we don’t see them at ANC. Also, when one has a child and we inform them of Child Welfare Clinic (CWC), then they combine that with the ANC, else they will delay until they develop some ailments or at their convenient time before they attend antenatal care. Some expectant mothers also feel they are too old to be pregnant again, so shyness keeps them away from ANC. Some also have unplanned pregnancies on very little babies because they do not accept Family Planning (FP), so when the pregnancy progresses, they feel shy to mingle with with other expectant mothers. Some expectant mothers also do not usually know they are pregnant until after the third month.

**Don’t you carry out education on family planning?**

We have been talking to them about FP uptake, but they condemn it. Hence, when they get unplanned pregnancies, they shy away from us and other women as well.

**What challenges do you face in providing maternal and newborn health care in your community?**

1. We don’t have a placental pit where we can dispose-off the placenta and other dirty fluid. We always have to tie them and give to the relatives of the of the women, which poses a lot of health threads because infection could be widely spread.
2. We have water problem. If the communities members do not draw water for them, it means we to leave patients and clients in search of water for our ourselves and to clean the facility.

*How about non-drug consumables?*

No, we don’t’ have any problem with those ones.

1. Just the NHIS has removed some essential medicines such as the antibiotics. They have been taken out of the approved drug list because CHPS compound is a small facility. if we prescribe it, they will not pay. Therefore, we sell it to them which they always complain they do not have money to pay for medicines. I have seen that we going back to the old system “cash and carry”. WHO and others’ protocol recommends antibiotics for expectant mothers who give bith newly, therefore, if a midwife conducts childbirth and cannot provide such essential medicines to treat the mother, how will the wounds heal?

**What general comments do you have to make for improved maternal and newborn health outcomes?**

1. If we can get some help at least for our placental pit. It’s really bad, because some expectant mothers come diseases such as hepatitis B, HIV, but we have to give it out to them. they don’t handle it themselves, they give it out to old people and then to the men. It goes through so many hands before it is finally buried and infection control procedures are not followed before burying them.
2. Our water tank is cracked. We do not have water at the facility also. If the community do not fetch water for us, we will not even have some for hand washing after attending to a client.

NAROKORINYIRI OPINION LEADERS

**When our women are pregnant, what work do they still do?**

***at home?***

They still do all jobs they were into, but not very hard ones. They do all the household chores – drawing water from the borehole, cooking, cleaning the compound, giving the children bath. The non-muslim brew pito for sale. They also attend ANC. It is a requirement for all expectant mothers to receive maternal health care until they give birth. She is supposed to also give birth supervised by the midwife at the clinic.

***at farm?***

Expectant mothers support in sowing seeds, and also cook for the workers at farm. They cut and convey firewood sometimes as soon as they are done with farm work for the day. However, they are unable to carry heavy firewood as when they not pregnant. Some also burn charcoal and log wood for sale.

**What traditional beliefs and practices do you on maternal and newborn healthcare in Naro community?**

We have herbs such as local oxytocin, which are administered on expectant mothers to ensure safe conception and smooth childbirth. The local oxytocin is more potent for positive labour than what is given at the clinic now. The local oxytocin is a multipurpose herb. It has the potency to stop pregnancy threats and complications. Labour is also very easy when the “hot” local oxytocin is taken orally. We however, wish government could endorse it for expectant mothers to freely administer it. The Traditional Birth Attendants (TBAs) were more skilful than the nurses we have now. The TBAs rendered antenatal care and there were usually no miscarriages during their time as it happens now. In the past we so many herbal practices which were done to ensure safety and healthy motherhood, however, now, Ghana Health Service (GHS) have stopped all TBAs from providing maternal healthcare.

**With your exclusive reliance on modern medicines, are there some health problems pertaining to expectant mothers and newborns?**

Yes, when women took only the local oxytocin, we don’t hear of stillbirths. Although, I belief when a neonate dies, it’s God who decides to take back for His personal reasons, and we have very little to do about it. I have a divergent view to the previous participants. The antenatal care particularly the medicines given to expectant mothers ensures speedy growth of the child when born. They begin to walk very early than before when compared to the era of the traditional medicines we used.

**What can we do to ensure healthy and safe pregnancies?**

There are many issues involved in this. Sometime ago, GHS asked the community to provide women who are TBAs to be trained so they routinely monitor pregnant women at the community level and render ANC services. It was very helpful, but later TBAs were stopped from providing antenatal care and conducting childbirths too. However, their services were very helpful. TBAs have now been warned of facing prosecution if they attempt to provide maternal healthcare.

**Do we have maternal deaths in your community?**

Yes, there are instances of maternal deaths.

***What were the causes of those deaths?***

This were because some of them did not take up antenatal care. The pregnancies resulted to complications which led to their death and lose of the unborn baby too. Our elders also believe there should not be sexual intercourse during the period of gestation of the woman. They said it can lead to death of the mother or complications or miscarriage. The midwife have broken that believe by asking expectant mothers to seduce their husband into having sex with them with the view to ensuring safe childbirth during labour. We believe the violation of this established belief is a factor to the death of some expectant mothers.

**When we fail to indulge in sexual intercourse with our pregnant wife, do we engage it with outside women?**

Not all men. We the elders are able to endure until her postpartum period, but the youth are unable to contain themselves. We have realised the medicines given at antenatal care have negative effects on childbirth. It causes the unborn have so much weight leading to difficulty during labour. It is a reason for the Caesarean Section (CS) operations, which keep some women in perpetual trauma.

**When women are pregnant, do they engage in full farm activities?**

Yes, they do when even asked to stop, they would not. also, assuming I have only one wife and due to pregnancy she fails to support in farm work, I will not be able to till the land and sow seed together with the weeding and fertiliser application all alone. Some pregnant women during the first trimester try to cut more firewood to store for use when they enter into the third trimester and the early postpartum period. When a woman is pregnant and engage in farming activities, it promotes easy and safe delivery. But pregnant women who not partake in farm work, usually end in CS operation during childbirth. Heat from the sun and the farm work are cheap forms of exercise to expectant mothers and ensures safe pregnancy.

**What is your perception about BPCR?**

Birth Preparedness and Complications Readiness (BPCR) involves purchasing items for the birth kits. When expectant mothers return from ANC, they tell us the midwife has asked them to buy cloths, baby clothes, razor blade for cutting of umbilical cord, parazone or dettol, a few white linen and put them together in small bag of basket pending the day of childbirth. It is not appropriate to wait until she is in labour before we start to acquire these items. The issues raised by pregnant women when they return from ANC classes includes purchase of birth kit and means of transport during emergency referrals of expectant mother. However, we usually do not prepare for the means, because we are always hopeful they will give birth at the clinic or home. When expectant mothers are referred, we then begin to look for motorbike or tricycle if she is unable to sit on motorbike.

**Are there other issues on BPCR you want to add?**

We have made little contributions towards making funds available for couples to borrow during emergency referrals to hospital, however, it is not much yet and no one has benefited from it so far.

**Are there some other groupings in the community where maternal and neonatal healthcare issues are discussed?**

The community have little contributions towards maternal and newborn healthcare called “Kaawonoma”. We make regular contributions of 0.20 pesewas during every group sitting towards this social welfare fund. Therefore, it’s discussed at every meeting. Aside the contribution, no issue of maternal and neonatal healthcare are discussed during our weekly Village Savings and Loans Associations (VSLA) meetings.

**What challenges are faced in maternal and neonatal healthcare in the community?**

1. The number of communities and population outweighs the CHPS compound.
2. Nurse absenteeism- The community members have often complaint to me as the Assembly member, of the fact that, each time a newborn or expectant mother was rushed to the clinic, the midwife is not available. Other times she refuses to attend to their healthcare needs, instead she complains they are few and need to keep off serving clients to be able to prepare food. Hence, nay minor sick case taken to thte clinic is immediately referred without any evaluation or administering pain killer in order to continue with her household chorres.
3. We need more nurses because, five communities – Korinyiri, Naro, Kanato, Sulaguo, Guori, and Kahu are served by one midwife. The midwife is supported by 1 community Health Nurse (CHN) and 1 Enrolled Nurse (EN).
4. According to Ghana Health Service regulation, the CHPS staff are supposed to work for 24 hours in 7 days, however, one day at a meeting like this, left the meeting to pick a labouring woman to the clinic and none of the three were there. So I brought her home and she had a homebirth. Meanwhile, each time we complain, she reacts wickedly to it by given more referrals. We need two or more midwives so we when one is gone to District Office, the others can serve the population. We have observed that, the midwife does not spend the weekend at the health facility, meanwhile, Naro/Korinyiri is the second most populated area in the district. Fian health centre is the nearest to our community, but it belongs to Daffiama/Bussie/Issa district. In the absence of our midwife, Fian HC does not accept expectant mothers, due to difficulty in retrieving health insurance claims from the different district. Naro/Korinyiri does not also have a straight and motorable route to Nadowli Hospital. The castigations we receive from Fian HC in addition to NHIS charges encourage labouring women to have homebirths instead. One day, I rushed my two years child to Fian HC and was refused treatment simply because Naro/Korinyiri is not part of their district. I was forced to return home and to mobilise more money and fuel and took her to Nadowli hospital for medical attention. She received needed care.
5. NHIS card registration and renewal is another factor affecting maternal and neonatal healthcare in the community. All registrations and renewal are done at Nadowli. This affects expectant mothers and newborns when the sick case demand urgency. We would be very glad if NHIS card registration and renewal could be decentralised to the sub-district level to ease us of long queues at Nadowli office. Many women are unable to afford transport fares because we don’t have “trotro” connecting us to Nadowli.
6. Supply of essential medicines. Medicines and vaccines are supplied to Jang HC before Naro CHPS to receive their stock. Meanwhile, these health facilities are in separate Area Councils. It further delays receipt of consignment for Naro CHPS. If stock could be supplied to Naro CHPS midwife, it will facilitate availability of vaccines and maternal healthcare medicines.
7. TBAs have been warned from providing maternal healthcare and supervising childbirths. Hence, with the nurse-absenteeism, it encourages prolonged labour in attempts to find means of transport to transport expectant mother to Nadowli hospital.
8. Naro CHPS have a vaccine fridge for storage of medicines and vaccines. However, there is no light in the facility, compelling the midwife to store vaccines at Jang health centre. Anytime, she conducts childbirth, she travels to Jang for the vaccine to give the newborn with Tetanus Toxoid Injection (TTI).

NARO/KORINYIRI NON-PREGNANT ADULT WOMEN

**Have you recorded maternal deaths in the last three years in your community?**

Yes, it’s been a year since we had one maternal death. An expectant mother died a few hours after having a successful livebirth.

**What are the cause(s) of the maternal deaths and still births in Naro/Korinyiri?**

Failure to seek maternal healthcare such as antenatal care and postnatal care. Some do attend ANC and PNC but are unable to complete the course. Others commence antenatal late of about seven months’ gestation.

When an expectant mother register for ANC, we are first referred to Nadowli hospital for laboratory tests and scans which some mothers refuse to go, because of the distance and cost of reaching Nadowli. Other times, if the husband has no motorbike and she happen not to have lorry fare to transit through Wa or Sombo to Nadowli, she wouldn’t receive laboratory investigations on time leading to late commencement of antenatal.

ANC medicine intake. Some expectant mothers complain of nauseating smell, dizziness, stomach upset among other reactions from the medicines and therefore refuse to take them or fail to complete the recommended dosage. These are lead to stillbirths, complications leading to loss of their lives. The woman who died went for antenatal only two times.

Distance to Nadowli hospital. Pregnant women foot for more than five kilometres to Sombo to join public transport (“trotro”) to Nadowli. Alternatively, others make two transit through Wa to Nadowli. As a result of the difficulty in accessing relevant care on time, it often leads to severe complications which could lead to stillbirths or loss of lives of both the mother and baby.

The rough surface nature of the roads is equally relevant for poor pregnancy and childbirth outcomes. When an expectant mother is referred via tricycle to Nadowli, many of those births were stillbirths or prolonged labour.

**What traditional beliefs and practices are done on maternal and neonatal healthcare in your community?**

Declaration or announcement of pregnancy- we have this rite in some gates of Naro/Korinyiri. As a result, even when a duly married woman conceives as prime, she has to wait in the first few weeks until the rite is performed. It is believed, if it’s not done, she might miscarry. She is unable to receive maternal healthcare until the rite is performed.

Early complications. Pregnant women in the past were enduring the pains and complications of pregnancy particularly in the first trimester and part of the second of the trimester. We realise so many referrals of young women at present because they are bold to complain and also unable to endure complications. We could endure pains until we even miscarry or have stillbirth at home without going to hospital.

***Do you administer herbs to pregnant women and newborns?***

We don’t dependent on herbs solely now. However, there some leaves that are very potent for treating malaria in children and expectant mothers. It’s boiled in water giving orally and bath as well. However, this is done usually done when medicines given fail or when her NHIS card expires.

**What work do pregnant women do?**

***At farm?***

we still do all jobs as when not pregnant. We burn charcoal, engage in farming activities, cut and carry firewood on headload, among others. We do them in order to earn a living.

***At home?***

We engage in cooking sometimes with big pots, pito brewing, cleaning of the compound, we draw water from the borehole, giving the kids bath, and washing of clothes of both kids and our husbands.

Some men these days do not go to farm again, leaving us to fend for ourselves and the children. We have witnessed cases of severe pregnancy complications which led to miscarriages and stillbirths among young ladies as a result of these menial jobs.

We don’t get any support from our husbands before and during pregnancy.

**What are the things women should do to stay healthy during pregnancy?**

We must not engage in farm labour when pregnant. We aware early commencement of ANC is good for healthy pregnancy and childbirth, although we are unable to begin ANC early. We are also always told during ANC to keep off naked fires by stopping pito brewing and charcoal burning so as to have safe and healthy pregnancy and child birth. We need more awareness creation among expectant mothers to know the significance of ANC, most especially among the young ladies and primes, so they can begin ANC early and continue through to childbirth. This will ensure safe childbirth unto a healthy baby.

In recent past, we combined both ANC and the care from the midwife and those of the TBAs. We have been stopped from receiving care from the TBAs, but some pregnant women still hide into TBAs homes for care and local herbs. In fact, TBAs sometimes palpate the womb so hard such that, some expectant mothers do get “black-out”. We embrace the services provided by TBAs because of the distance to Nadowli hospital and absenteeism of the midwife at our clinic. Currently, women who give birth at home are fined so as to encourage all women to have births supervised by the midwife. I have had a painful stillbirth which changed my attention from TBAs to the clinic. I have walked to Sombo twice to join means to Nadowli hospital after I endure some painful treatment from the TBA though I didn’t have a successful birth too. If others will not go to clinic, but I will continue to receive maternal healthcare from the clinic. The ANC medicines are so helpful, even there child walks faster than expected. We need more midwives who are also regular. Most often, we don’t meet the midwife when a case is send to the clinic.

*Some women still have homebirths when they get to the clinic and doesn’t meet the midwife. Expectant mothers often consider the distance to Nadowli and realised she might not reach before labour intensifies. Instead of having childbirth on the road to Nadowli hospital, they sometime stay at home to have their childbirth.*

**If someone talked to you about birth preparedness and being ready for complications in pregnancy, what do you think they would be talking about?**

Birth preparedness and complications readiness involves saving money and purchasing baby clothes, cloth, line, soap, Dettol, blade, and a thread. BPCR means getting my birth kit ready for the day of childbirth. Other forms of BPCR are secondary to us. The midwives have talked about getting blood donors but we think about it when expectant mother is asked to bring donors.

One day at a community gathering, we were asked to contribute one Ghana cedis (GHS 1.00) each so that in case of emergency referral of pregnant women who are more important, one can access that quickly and later make a refund for others to also benefit from. The midwife has also spoken to opnion leaders about CETS – community emergency transport system initiative was established to improve upon MNH care through referrals. In our community, the trotro drivers who ply Naro/Korinyiri – Wa road are very inconsiderate during emergencies. When they are approached, until they are provided full fare they don’t move an inch. This CETS initiative when implemented will alleviate the plight of pregnant women during emergency referrals. We have made contributions ones, but it’s not enough to hire a car. A lot of expectant mothers have received lessons on BPCR, but poverty makes them unable to prepare in advance.

For instance in my recent childbirth, I borrowed money from our women’s group. One day when I felt I was due to give birth, I walked up to the clinic and was referred to Fian HC. On arriving at Fian HC, I was further referred to Nadowli hospital. However, I was unprepared for birth, so I walked back home to get my birth kit and headed for Jang HC which was nearer than Nadowli hospital, because, I met no family member. I got off to the roadside and found trotro to Wa. So I instead went to Wa hospital. I gave birth as soon as I was taken into the labour room, because I felt the initial stages of labour whilst in the public transport after the second referral. It’s indeed helpful for all pregnant women to prepare to avoid such pain and disgrace.

Our husbands also need education. Sometimes, even when in labour pains, they do not care about us. They can just get up and go anywhere or to farm at the time when they are most needed.

**Do the men take or accompany you to ANC?**

Men do not accompany us to antenatal care. It’s of recent some youth have began supporting their wives to seek maternal care at the clinic. Sometimes, because of the farm work, instead the man accompanying us for ANC, we are happy to go alone whilst he continues with the farm work.

***What if you get to the clinic and complications set in, how will you get him informed of the need to support on the referral?***

In each section of the community, we have a number of expectant mothers receiving antenatal care on the same day. In case of emergencies, they report to your husband or your next door neighbour to assist in the absence of your husband.

**What can you tell me about community initiatives towards ensuring safe motherhood?**

We have no community initiative for safe motherhood. When it’s critical on an expectant mother she happens to be a member of any of the “susu” groups, they borrow that to receive care in the referral health facility. We have made some contributions as a community, but we don’t know how the opinion leaders are managing the fund, whether it’s embezzled or not. It’s been one year now since we made contributions of one Ghana cedi (GHS1.00) to the fund by each family. We have never been updated of it’s management and availability.

***So in case of severe complications and she is unable to foot as usual, how does she get to the referral facility?***

Expectant mothers who patronise ANC for all the months of the gestation are usually informed of her place of childbirth by the midwife and the kind of childbirth. If it’s the risky conception, the midwife often asks her to move to Nadowli hospital to have bed rest pending childbirth.

In one of my conceptions, I walked up to Jang HC was referred to Kaleo because their midwife was not available. When I arrived at Kaleo, I was further referred to Nadowli hospital. As soon as I got off the vehicle, the baby came out. Therefore, if I hadn’t left home early to seek care, I would have had birth on the footpath alone or in the public transport to Nadowli.

Having the NHIS card renewed regularly is very helpful particularly for expectant mothers. In a particularly day, I was referred to Nadowli but I could not sit on the bike alone, so I was put in between two people (the rider and my mother-in-law) to Nadowli. Labour did not progress as expected-prolonged but I finally gave birth successfully to a live baby.

In my last child birth, I discovered I was due to deliver, so I left off for Fian HC as I began to feel the movement of the baby. Just at before leaving the community, labour began. So I asked my husband to call on the nearest woman to assist me because the baby was about to come out. Before he returned with a woman, I had given birth by the roadside. She came to cut the cord and supported me convey the baby back home. However, prior the labour, I gave the midwife a phone call and she was at Wa. Hence, I had no alternative but to head off for Fian HC which is the nearest facility.

**Do women still have home births?**

Yes

**What do you think stops women from wanting to give birth in a health facility?**

Expectant mothers usually do not plan to have childbirth at home. It happens when we realise we might not reach the facility before giving birth or when we get to the clinic and the midwife is absent, we resort to home birth in these instances.

**What reproductive cultural practices exist in your communities?**

We don’t have any cultural practice again. We practiced female genital mutilation but we have been warned of facing prosecution should we be caught in it again.

**What do you think stops women from seeking antenatal care?**

Some expectant mothers say conception is not a sickness. Such women wait until complications set in before they seek maternal healthcare. Pregnant women who don’t receive antenatal care are mostly the adult women. Although, there has been increase in patronage now.

**Why do pregnant women not have safe pregnancies?**

Some women refuse to take the ANC medicines especially the three tablets (Fe3 acid). They often complain it has nauseating smell. Others vomit after taking it. Some expectant mothers refuse the medicines with the perception, the foetus will put on weight which might lead to CS delivery. Many fear CS childbirths so they refuse the medicines. however, it’s a perception of some women. I have always taken the full dosage but have had normal childbirth to live babies.

**What can you tell me about other settings (places) or groups where pregnancy related matters are talked about in the community?**

Discussions on MNH care is not common here. It’s once this was mentioned concerning NHIS registration and card renewal. We are asked to borrow from the susu box to register and renew NHIS cards and refund later. Aside that, there has never been any issue on MNH care during our weekly VSLA groupings.

**What will you suggest should be done to prevent pregnant women and new-borns from dying?**

1. The clinic should be upgraded to a polyclinic, so more nurses can serve us. The drug list under the NHIS is also determined by the kind of health facility. clinic can only provide pain killer and conduct routine national immunisation activities.
2. More nurses especially midwives should be posted to the community. The current midwife should also be stable and available at all times to attend to emergency cases of pregnant women and neonates.
3. The road to Nadowli is very deplorable. One time, an expectant mother had a still birth whilst on a motorbike to Nadowli hospital.
4. Expansion of childbirth and ANC room. We have only one bed each for ANC and delivery. Women sit outside the veranda during ANC. Therefore, during rains, some are send back home because there is no hall to contain all.

NARO/KORINYIRI YOUTH

**If someone talked to you about birth preparedness and being prepared for complications in pregnancy, what do you think they would be talking about?**

It entails receiving regular antenatal care and other relevant maternal healthcare such as the doing scans and laboratory tests. when one says she has prepared for birth, it means she has purchased soap, baby bathing items, and keeping your ANC record card with her always. When you are due to go to the clinic, you pick the items and go up to the clinic when you realised you about to go into labour. BPCR means that when expectant mother knows she is due to give birth, in about her ninth month of gestation, she can begin to buy all items for her birth kit as recommended by the midwife and keep them at a known place to all family members, so even if labour begins outside, anybody can pick them up for her to rush to the clinic. She has to grind flour, process shea butter and keep them for use when she gives birth. During her ninth month of gestation and her immediate postpartum period, she might not be strong enough to do all these.

**What are your perceptions of safe pregnancy?**

Safe pregnancy means expectant mother attends ANC regularly and on time. When she is regular, any risks associated with the conception will be detected early and treated. A pregnancy is said to be safe when you do little or no hard work. A pregnant woman who does not engage in strenuous activities such as charcoal burning usually has fewer health problems than those who indulge in them. In recent past, I climbed trees in search of firewood and dawada fruits whilst pregnant. As a result, I had severe complications throughout that pregnancy which even led to stillbirth. Safe pregnancy implies, she is being eating nutritious food so the foetus is healthy too. When expectant mother refuses to seek maternal healthcare, the partner ought to compel her so the pregnancy can be safe. We are very much aware some expectant mothers refuse to take the ANC medicines, though it is paramount for safe pregnancy. Some expectant mothers are also misled by their colleagues to use “cold” local oxytocin, meanwhile it’s has adverse effects on pregnancy safety.

**What are the various kinds of support pregnant women receive from family members**?

When a woman conceives, we encourage her to go for ANC, so the midwife can educate her on things to do and those not to do. She further receives education on eating habits and the recommended food ingredients for her wellbeing. We provide expectant mothers with the food items. We also allow them to do the amount of farm work within their ability. We don’t give them tedious jobs to do again, particularly leaving her on work involving fierce sun heat. Also, if she could sow two acres of tilled plot of land a day, we don’t allow her to complete two acres again in a day. We discourage pregnant women from doing jobs that involve having to bend for long period of time, such as sowing of seed and fertilizer application. We also continually ask of her health and that of the foetus, and further monitor to be sure they do not administer local oxytocin.

I was lucky, my family exempted me from farm labour during my pregnancy. But this was done after I suffered prolonged labour in my third childbirth which nearly killed me.

***What of the firewood, do you cut it for them?***

Yes, but not all men. Some men and non-pregnant women and the family do support in felling and conveying firewood home for expectant mothers’ usage. However, some pregnant women still do everything as before the conception.

Frankly speaking, in the entire community, real care for pregnant women and newborns as recommended can only be done by only few families. In the entire community, the reality remains same as when women are not pregnant. If one was into charcoal burning, pito brewing, doing farm labour, logging wood, felling and conveying firewood, are what they still for a living. We aware of the risks, however, there is very little we can than to involve pregnant women continue with these hard activities.

Expectant mothers continue to suffer in menial jobs until child birth. When men till the land, it’s women – pregnant or not pregnant, who do the sowing. When it’s harvesting, expectant mothers are compelled to do the harvesting. Sometimes, it’s only the women who complete harvesting and convey everything via headload home. I have sold logs of wood to buy birth kits and for livelihood.

**During complications, by what means do you seek relevant maternal healthcare?**

If she can sit on a motorbike, we use that, otherwise, the dominant means for emergency referrals is motorking/tricycle motorbike. Some men even use bicycle if they are unable to get a motorbike. Other times, if the woman and her family are unable to hire motorking and happen not to have motorbike, then expectant mother in question walk to the facility. expectant mothers have walked to sombo in pain when no family members had gone to farm or funeral.

**What arrangements are there for blood donors during emergency?**

We don’t have any established arrangement for blood donors. But during emergency, the husbands usually go into houses in search of volunteers to go and donate blood. When the health facility is far, for instance Wa or Nadowli, it’s usually frustrating getting volunteer donors, particularly if the husband cannot afford their lorry fare to and from the hospital. volunteers have returned from blood donation only to be provided with local gin “akpeteshie” as drink for accepting to support save lives.

**Do you administer herbs on neonates as form of treatment for sickness?**

Yes, we do boil leaves and give them orally and also use some for warm bath. This is particularly done when the NHIS card expires and there is no money to renew it at the time of the sickness. We also boil the herbs and give them the bath because, when we don’t have money and to the clinic even with NHIS card, they prescribe the medicines for us to buy with our money from the chemical sellers, which we are unable to afford. Even when the child responds slowly to the medicines, it is the herb and leave intake that mostly cure them. *some children are re-incarnated of our ancestors, therefore even if we treat them with the medicines prescribed, the sickness still persist until we gather appropriate herbs to treat them.*

***When expectant mothers experience pains and complications, do you administer herbs on them?***

No, we don’t treat pregnant women with herbs. But there is a spiritualist (elderly woman) whom we usually go for maternal healthcare. When she attends to a pregnant woman in complications, she recovers very fast.

***I learnt expectant mothers in your community do not seek maternal healthcare from anywhere apart from the clinic. Is it true?***

It’s not true. We hide and seek maternal healthcare from TBAs and the spiritualist, although we combine both clinic and local. Even with childbirth, some women still go to TBAs for delivery care. Others also deliver at home supervised or not by family members.  *Here, the men get happier when we give birth at home, because he will not incur any cost purchasing birth kits or transporting you to Nadowli or Wa hospital when referred. Anytime we give birth at home, our husbands make sacrifice with animals to his ancestors for ensuring safe childbirth of his wife at home.*

Expectant mothers who have had complications in their past pregnancies are the ones who do not seek care from TBAs. For instance, I saw how one of us suffered for two days in labour which finally led to stillbirth and obstetric fistula. Therefore, when I was due to give birth and family did not show interest in supporting me reach health facility, I looked for my own way, so I do not have homebirth. One reason I do not seek care from TBAs is, if I get dehydrated or anaemic, they are unable to diagnose that. If I require blood transfusion or a drip, TBAs do not provide those services for you to regain strength. They are unable to resuscitate the neonate if the need be. The local oxytocin also causes preterm births which leads to stillbirth. Illiterate women are the major customers of the TBAs.

Some men are very inhuman. When she had the fistula, the husband threatened marrying another woman, so she took in and gave birth to a baby girl whilst in the same condition. The midwife told her to stop giving birth until she is repaired, but she was forced to conceive again. She is being avoided by community members and she is unable to attend public gatherings and funeral. She is compelled to carry with her three additional cloths to change her dress regularly, due to the foul smell.

**What effects do you think the intake of herbs has the patient?**

The local oxytocin is very helpful to us. I have realised that each time, I’m in my second and third months of gestation, my feet get swollen. Hence, I do take the “cold” local oxytocin to cure the ailment. We resort to traditional medicines because of the cost of fuelling someone’s motorbike to send us to hospital. Also, anytime, we seek care medicines and even drips/infusions are prescribed for us to buy. Even with the NHIS, it is only the folder we usually benefit from, but all medicines are prescribed for us to purchase from chemical sellers.

The frequent vacation of the clinic by the midwife is serious factor to this practice. I went there consecutively for 3 days but could not find her to provide care to my baby. I didn’t also have money to go to Naodwli hospital. in my last conception, I went up to the clinic at about 1pm with minor bleeding and headache and was asked to wait on her to get food to eat before attending to me. The headache and bleeding began at farm. *i don’t have a child yet but my sister recently run up there with the baby who suffered convulsion and also to leave the baby and go back home and fetch water for the clinic before she would attend to the baby.* All these issues account for our dependence on traditional medicines for treatment. *The chucky replies to our healthcare requests discourages us from seeking MNHcare at the CHPS compound.* The pregnant women do draw water on headload for the nurses each time they are seeking care.

Carrying headload for the nurses during ANC is really a disincentive and a burden on us. Even when we have pains of complications, we are compelled to still draw water for them. *I was had some pains of pregnancy, and when I got there, the midwife asked me to go back and draw water for her. Meanwhile, the town is more than 2kilometers from the health facility.*

Do you draw water for the nurses when you are due?

Some expectant mothers do it when they have no family member to fetch it on behalf of them.

**What do you think can be done in the community to improve the health of pregnant women and newborns?**

We need regular education on health alerts and ways of preventing disease infestation. Ignorance of certain relevant health issues affects the outcome of pregnancies and newborn health.

More midwives should be posted to our place, so we are not referred each time she is attending to an expectant mother or when she is absent.

Supply of essential medicines to the facility. We sometimes get to the facility to realise there are no medicines to treat us, and are forced to continue to Nadowli hospital or Wa hospital.

Nurse absenteeism. The midwife is usually absent from the clinic further worsening our plight. Government should ensure the nurses are resident to provide the 24/7 healthcare services. The nurses should also provide care during the weekend. Sometimes, when we go there with a case, we are they are resting. Other times, they vacate the clinic during the weekends.

Government should further provide residential accommodation for the midwife, to motivate her to stay with her family to avoid the regular absentia we experience each time.

The clinic is further causing marital instability in the community. We have realised that, a lot of assault cases among couples originate from the family planning services provided. Women are given the devices/pills without the consent of the husbands, and when detected, they react violently. Non-pregnant women trick their husbands they are off to cut firewood or farm, but branch into the clinic for FP injections. After sometime, when is not conceiving as expected, the men explode into quarrels. *We don’t know if it’s woman planning or family planning.*

*We the women do it because our husbands do not support in pregnancy and care for the children. When we realise, it’s same pattern of behaviour with every child we have, we take up FP to control births so we care take care of the ones already born. We understand, there are agencies, which can force irresponsible husbands to be responsible, but it will eventually break-up the marriage relationship. It is the reason we endure for some births and resort to FP without their consent to be able to bring up the ones born.*

*As a health surveillance volunteer, I attended a meeting somewhere and a woman run into our midst whilst crying for help, for the couple had given birth to unnecessary number and the husband was irresponsible but further pressed on her to conceive. The nurses at the meeting asked her to come over to the clinic with the husband which she failed to do. The woman gave in to the pressure and conceived again which resulted in prolonged labour and she died. During maternal death audit, the nurses were blamed for not providing her with FP service at the instant she rushed into the facility.*

NARO/KORINYIRI FACILITY HEAD

**What comments will like to make on MNH care provision in your catchment area?**

The community has the traditional practice on newborns, they belief the first bath must be cold water, which can affect the health condition of the neonate. The baby can get cold out of that. The baby can even suffer pneumonia too out of that and most children die from cold in the first initial days because the uterus is usually warm at birth and have to be maintained, hence, when they go to expose the neonate to cold water, the child can die. Another thing is that, they belief in using shea butter to apply on the baby’s cord. Although there is education ongoing, you know it is not easy to change people’s belief systems.

***Does the shea butter application have some effects on the baby?***

It has an effect. If the shea butter is not well processed, the child could get sepsis infection because it is on the umbilicus they apply it on. The baby could even suffer tetanus infection out of that. Then another issue is, especially when we are in the warm season. When they give birth, the baby sweat, so when that happens, they think the baby must be exposed to cold. Most babies are like that. I know newborns do not know warmth, so they ought to still maintain their warmness, even when sweating. If the baby get exposed to extreme cold as they have been doing, the child can die out that.

On maternal health, usually when they are in their ninth month, they are expecting labour especially when they know their Expected Date of Delivery (EDD). Once the EDD elapses, they don’t come to the facility, rather they go to the TBAs for “hot” local oxytocin. It’s taken orally and also smeared on the tummy to speed up the process of labour, thus to induce labour early. In such instance, expectant mother gets irregular contractions, severe ones and when they don’t get to the facility early for immediate care, the breath of the foetus will reduce. So the child ca get asphyxia. In case it’s a homebirth, the baby may die. However, if childbirth is in the facility and detected early, we use the ambu bag to resuscitate the baby.

***So do you have the ambu bag?***

Yes, I have the ambu bag.

Distance and nature of the to the clinic by some communities – Kanato, Sulaguo, Zokyieriguor poses serious challenge to receiving prompt maternal healthcare. It’s only Kanato, that is accessible by car, but the others cars cannot go. Due to the distance, when I am informed of the time the labour or complication, it is impossible to reach the facility. CETS initiative is in process, but it is not yet fully implemented.

***Do you have a place for disposing off the placenta?***

Currently we do not have. I think not all CHPS facilities have placental pits in the district. Last year, I spoke to the community leaders and they organised and dug the pit, but where to get money to complete it is now the problem. Whenever I conduct childbirth, I pour parazone on it to disinfect the placenta and give it to the client to go home and burry. That is what we practicing currently.

***Are there some other issues affecting your work – community perspective, NHIS or any area?***

Not really, but for the NHIS, it is free for expectant mothers so they rush to register when they conceive.

JIMPENSI/KENKELLEY OPINION LEADERS

**What kinds of things/activities should a woman do when pregnant in the community?**

***At work?***

Expectant mothers partake in farming activities – sowing of seeds, fertilizer application, having of crops and drying/processing of farm products. They also cut firewood for family usage as well as burn charcoal for income. All the activities expectant mothers do when they were had not conceived are what they still do, but not the very difficult ones such as working from morning until sunset. Farm work is a form of exercise for expectant mothers.

***At home?***

Pregnant women continue to do cooking, cleaning of compound, fetching of water for household use, washing of clothes of the family, and giving the children a bath.

**What can you tell me about community initiatives towards ensuring safe motherhood?**

The community health officer once had a meeting with us on issues pertaining to emergency referral of pregnant women in particular. Hence, we have agreed to the CETS initiative and have established a Common Fund for families to borrow from during emergency referrals. Therefore, in case of emergency referrals, the family is able to secure a portion to hire a car or motor king and also pay other hospital bills, but make a refund of the money for others to also benefit from. We also encourage and exempt them from farm work to receive antenatal care. When they are due to give birth, we also ask them to go to the clinic. We do not encourage home births ever since we got the clinic.

**What activities (cultural, social, economic) should pregnant women not do in your community?**

Expectant mothers engage in so many activities. For instance, burning charcoal, brewing of “pito” and carrying firewood on headload are the dominant economic activities done by women. When farm produce are harvested, it is women whether pregnant or not, who convey most it home. During funerals, expectant mothers are as active in all funeral activities ranging from mourning to serving visitors.

***Do you resort to herbs for MNH care in the community?***

Yes, we practice traditional medicine. We usually boil some particular leaves and use it to bath the neonate. Others are given to babies who can drink, thus if the sickness is with the stomach. This happens mostly when we do not get anticipated care from the health facility. Whenever, medicines from the clinic fail to treat, we administer our herbs and leaves and if lucky, they become healed. However, many cases are first send to the clinic before we administer traditional medicine on it.

**What do you think are the main causes of deaths of pregnant women or after delivery in this community/district?**

We use to have frequent deaths; however, with the construction of the clinic in the community, it has reduced. We had a recent death of an expectant mother who was on referral to Jirapa Hospital.

***What caused it?***

Women who died during childbirth or whilst pregnant were those who did not patronise antenatal care. Others take “cold” local oxytocin when there are minor pains of pregnancy, instead of seeking care at the clinic or hospital.

**If someone talked to you about birth preparedness and being ready for complications in pregnancy, what do you think they would be talking about?**

BPCR means getting delivery items/birth kits the midwife ask to them to buy. BPCR means receiving antenatal care. Preparedness involves attending ANC so that if there is any risk with associated with the pregnancy, the nurse will treat it or advise her. BPCR also talks about getting her NHIS registered so she can access maternal healthcare with ease. If the husband is not financially sound, he has to save money for emergencies.

**What can you tell me about other settings (places) or groups where pregnancy related matters are talked about in the community?**

We have weekly routine village savings and loans meetings in the community, during which the CHO comes around to educate us particularly during outbreak of diseases.

**What forms of support do these groupings offer to pregnant women?**

The groups do not offer any support to expectant mothers. We discuss matters concerning to our little savings and who will collect the next micro savings, not MNH issues.

**I discovered some expectant mothers do not attend antenatal care. Why is it so?**

Some expectant mothers deliberately refuse to go for ANC. We the men have done our best to encourage them to receive ANC even by helping them renew their NHIS card, and yet they still refuse to go for ANC. It is just an attitude in them. Such expectant mothers also deliberately give birth at home. We have women in this community who have never given birth in a health facility. As a result, we have instituted a fine of ten Ghana cedis (GHS10) for any homebirth to discourage them. Others report late when in labour, thereby unable to sit on motorbike or even reach the clinic before they give birth. The fine is small, making women to still give birth at home, since they can easily pay it off.

**What sorts of things does your family do (or do families do) to help ensure safe pregnancy and childbirth?**

We do not support pregnant women that much in this community. For instance, in cutting and conveying firewood home or going to the borehole to draw water for them. Nevertheless, we help them lift load up their head if men are around. When they are sick, we help in bathing the children and cooking. We also allow them stay at home from farm work. We further stop them from all forms of farm labour when they are due to give birth. Some pregnant women still do all kinds of hard labour until childbirth because, when we exempt them from family farm activities, they sneak into their own private work of farming or charcoal burning, no matter the pain they go through. Such pregnant women do not get any form of family support throughout their gestation.

**What do you suggest are the roles of men in pregnancy?**

Men are supposed to take to expectant mothers or their babies to hospital during emergency referral. It is our responsibility to give money to pregnant women to renew their health insurance and give ride to Nadowli for laboratory investigations.

**Are men active as expected in supporting the woman during the period of pregnancy?**

No, some men only impregnate and leave them to manage the pregnancy until they give birth. Some do not even take them to Nadowli to renew their health insurance and laboratory tests. The pregnant women fuel other relative’s motorbike to take them to Nadowli for labs and NHIS card registration and renewal.

***Where do you have your NHIS card renewed?***

All community members go to Issa but pregnant women go to the main office at Nadowli for their card registration and or renewal. It’s factor for the delay in commencing ANC or some do not take up because they are unable to renew their to benefit from the free laboratory tests and free ANC.

***How about laboratory tests/scans?***

All laboratory tests and scans are conducted at Nadowli hospital. Therefore, not all families are able to afford transport fares to Nadowli to carry out laboratory tests, particularly with the expectant mothers.

**What do you think are the best ways to prevent maternal deaths and still births in the community?**

One, expectant mothers must make they have childbirth at health facility. Women who deliver at home before going to the clinic for newborn immunisations must also pay fines.

Government should provide all relevant medicines under the NHIS. We have realised that, each time we go to the clinic and hospital for care, the nurses/physician assistants give medications in prescription forms for clients to make out-of-pocket payments for medicines.

This has made NHIS subscription and renewal of no relevance, thereby encouraging everyone to purchase medicines direct from chemical sellers anytime one falls sick.

JIMPENSI/KENKELLEY NON-PREGNANT WOMEN

**I was told there were some maternal deaths in your community; what were the causes?**

The deaths resulted from the absence of clinic in the community. Hence, during complications or emergency childbirth referral to Nadowli or Wa hospital, due to distance of more than 30 kilometres, some usually do not get to the facility before their death. Nonetheless, with the availability of midwives at Issa and Kojokpere, we are able to give birth at Issa or Kojokpere health centres. Jimpensi CHPS facility is also able to conduct emergency childbirths. This has reduced maternal deaths in the community.

***Were some deaths as a result of homebirths?***

Yes, due to non-availability of midwives and CHNs, all childbirths were done at home supervised by relatives and adult women. The TBAs were also a factor to some complications which led to death. Sometimes, there will be no sign of the baby coming out, but they will apply the hot local oxytocin and forced them to “push”, regardless of whether baby was in breached position. In the past one year, we had a case of maternal death. She refused to seek ANC and also attempted a home birth and died a day after a livebirth. We cannot force women who do not seek maternal healthcare. Some husbands support their failure to seek MNH care. Other men pressed on making sure their wives patronise maternal healthcare at health facilities.

*I have requested for money to seek maternal healthcare from my husband and was not given. I had to struggle to acquire and fuelled a bike to go to Nadowli hospital for laboratory tests. My husband deliberately refused to support me nor take me to the hospital. Other men have been cited saying their mothers did not receive ANC but gave birth successfully*

**What are the causes of Neonatal deaths in your community?**

We still experience stillbirths so much in our community. Young women are prepared to sacrifice their lives for money. Therefore, even when pregnant, they engage in wood logging, charcoal burning and worst of it “galamsey mining” operations to create wealth. They indulge in risky businesses with the view that, their husbands do not provide for their needs. Many expectant mothers who engage in charcoal burning and illegal mining activities usually have stillbirths. The heat and the dust causes preterm births. *During antenatal care, the nurses ask us to purchase birth kits and cloths for ourselves, so if I do not do menial jobs, I cannot acquire those items.*

***Do your husbands support in purchasing birth kits?***

No, they do not support us. They do not even give us money to mill flour. We are thereby compelled these circumstances to log wood, burn charcoal and brew “pito” to earn a living. All the men in the community are the same. No man provides for the needs of expectant mothers. We fend for ourselves, however, they provide the raw foodstuff from the farm which we all for.

**What things/activities should pregnant women not do in your community?**

The main activities here for all women are: farming activities – harvesting farm produce, sowing of seeds, clearing of farm for ploughing, charcoal burning, pito brewing, cutting and conveying of fire wood, gathering and collection of shea nuts, and harvesting of dawada fruits. Whether expectant or not, we all partake in these activities for livelihood. Being pregnant is even better doing these jobs than women with babies are. We carry out all these, and still come to draw water from borehole to cook for the men and the children.

**What activities (cultural, social, economic) and general daily lifestyle should pregnant women not do in your community?**

We understand the dangers/risk involved in climbing trees, burning charcoal and pito brewing. Expectant mothers ought not do these jobs or carry headload. However, we have no alternative then do them.

**What are the things women should do to stay healthy during pregnancy?**

Women with precipitous labour need to be prepared always. Those with epidural labours might be able to get to the relevant referral facility before giving birth. As a result, expectant mothers ought to always take money with them so they can easily access emergency care at any time complications set in. Seeking care from TBAs is unsafe to do, however, we are unable to go to Nadowli with ease for NHIS subscription and laboratory tests. Homebirths are not also safe, once we have a clinic and a midwife at Kojokpere to provide skilled care.

**If someone talked to you about birth preparedness and being ready for complications in pregnancy, what do you think they would be talking about?**

It means saving money for means of transport when pregnant woman is referred to Nadowli or Wa hospital. BPCR also entails getting birth kits and ensuring you keep it with you at all times when due to give birth.

**What can you tell me about community initiatives towards ensuring safe motherhood?**

*I was chosen in the past from my community as a TBA for refresher training and skill upgrading at Issa and was later moved to Kojokpere because of distance. After the training, I monitored expectant mothers and gave them education on safe motherhood and danger signs of complications. I was initially using bear hands to conduct deliveries but was given disposable hand gloves and delivery bed by GHS. However, GHS later stopped me from offering maternal health services. We have been asked to refer all deliveries and complications to the clinic and hospital. I have taken several labouring women and issues of complications to the clinic.*

**What can you tell me about other settings (places) or groups where pregnancy related matters are talked about in the community?**

Issues of pregnancy and child welfare are not discussed anywhere in this community. We have weekly meetings, but no one has ever been there to discuss anything aside our microcredit matters. We don’t also talk about maternal and neonatal health at our group meetings.

**What forms of support do these groupings offer to pregnant women?**

The only support pregnant women do get is borrowing money from the savings we contribute as microcredit. But it’s refundable without interest.

**What sorts of things does your family do (or do families do) to help ensure safe pregnancy and child birth?**

Some men do give their wives a ride to Nadowli hospital or Wa hospital for relevant maternal care. Normally, the men exempt expectant mothers from farming activities, but they do not support us in any way again to receive care.

During referrals, men care about the wellbeing of their wives support them to reach facility. Nevertheless, majority of them do not care how expectant mother on referral would reach the referral centre. Some expectant mothers do get family support for NHIS subscription and renewal. Some men offer their wives lift with bikes to Nadowli office. This usually take place when expectant mother had already registered for ANC, so she would combine both laboratory investigations with the card renewal. Many men support in NHIS subscription because, they know very that, during complications and birth referrals, it will be very costly for them to bear; therefore, many give maximum support to subscribe us to NHIS. However, after, the subscription or renewal of card, whether expectant receives maternal care or not, many men do not care about that.

When it comes to buying prescribed medicines at care seeking, men do not support with money. Husbands are misconstrued with the view that, ANC and NHIS registration are free for all expectant mothers, therefore, they should not request for money. Aside, ANC, expectant mothers ought to take money with them because complications could set inn unexpectedly and usually in the absence of family members. *Some pregnant women do not attend ANC, when they have unplanned pregnancy on a non-walking baby. They stay off the public domain due to shame. Such pregnant women receive ANC when they are already in the third trimester. Others do not seek maternal healthcare until labour triggers.*

**What reproductive cultural practices exist in your communities?**

In the past, we practiced female genital mutilation in the community. We conducted FGM to preserve them from premarital sex and promiscuity when married. However, the government has threatened prosecuting us if practice it again. We know it was good, we had no teenage pregnancy, but it is very common now. Teenage pregnancy has really been a burden on us, of having to care for both the mother and the baby.

***Are there some other beliefs and practices on maternal and newborn healthcare in the community?***

Yes, we belief and practice “pregnancy announcement” for customarily married primes. It is done to commit the “seed” to the ancestors to keep it from miscarriage. When this is not done and it is mentioned to the prime’s hearing, she can miscarry.

***How long does take; into the pregnancy before the rite is performed?***

It depends on the “waist” of the woman. If it is detected early, we quickly conduct the rite to preserve it. However, some could take up to three months of gestation before the rites are performed. Many of the “announcement” are performed when she is the third trimester. Such primes, do not commence ANC early.

**What do you think stops women from seeking antenatal care?**

When we go to register at the clinic for ANC, we are referred to Nadowli for NHIS card renewal and laboratory tests. The distance to Nadowli is major factor for many mothers not receiving maternal healthcare. Many people in the community do not subscribe or renew the subscription until they are sick or pregnant. Secondly, some wait until they conceive, so they can enjoy the non-premium subscription. Once, expectant mother is unable to for laboratory, she cannot continue to receive antenatal care. Some expectant mothers deliberately refuse to seek maternal care even when they have done the laboratory tests. Heartless men rejoice when their wife refuse to attend ANC and join him at farm, particularly if it is in peak farming season. Those very men are happy when their wife give birth at home, because he would not incur any cost hiring means for emergency referral. Jimpensi clinic does not also have vaccine fridge for storing vaccines. When there is not means, we are referred to Kojokpere health centre for ANC injections. Those referrals sometimes end some mothers ANC attendance. Footing to Kojokpere discourages mothers from receiving the care and ANC from the midwife. Even some nursing mothers fail to receive immunisations due to the distance to Kojokpere.

12. b. What do you think stops women from wanting to give birth in a health facility?

It is a deliberate attitude of some women to give birth at home. *I have had five childbirths at home. I never received maternal healthcare.* Some give birth at home to show her prowess. *I planned to give birth at the facility because I made eight visits, but on my way from farm, I gave birth and had to refuse to receive immunisation at the clinic because, the nurses would insult and embarrass me.*

***Do the nurses inform you of your expected date of delivery during ANC?***

*Yes, but sometimes, we do not know remember the date. When we tell some nurses of our last menstrual cycle, but they are unable to advise us on the possible date of childbirth. So we also just wait until labour sets in.*

**What do you think are the best ways to prevent maternal deaths and still births in the community?**

The best way is to go for ANC regularly and eat nutritious food. There are recommend food items and fruits on our ANC card. Nevertheless, it is hard to obtain those fruits, so we just try to eat food, which can sustain us through the period.

**What will you suggest should be done to prevent pregnant women and new-borns from dying?**

Taking good care of yourself to have healthy baby. Having regular bath and eating regularly.

The men should be involved in ANC and other maternal healthcare services. The nurses do encourage us to attend maternal care with our husbands but it is not working.

Women should take up ANC early, so they will take all essential medications, which will ensure immunity of the newborn and the mother. Some expectant mothers refuse to go for ANC and ANC and instead to farm or engage in their businesses. Some nursing mothers attend ANC for less than a year, instead of the full 5 years.

*I don’t patronise maternal healthcare because of my husband unfavourable decisions. When there is farm work, instead allowing me go for healthcare, he will provide huge description of my daily work so that I would not be able to take up maternal healthcare. When I insist on receiving care, I am called a lazy woman – who continually dodge farm work.*

*When I conceived for twins, my husband told me I was lazy so in order to save my relationship, I persisted on doing seed sowing and other farming activities until childbirth. During birth, one came out dead whilst the other was livebirth. The stillbirth was as a result of strenuous farm work.*

ISSA HEALTH CENTER – MIDWIFE FOR DUANG CHPS AND ISSA HEALTH CENTER

**What are the challenges you face in providing maternal and neonatal healthcare in your catchment area?**

Here, our main challenge is our transport system. When we are to refer expectant mother to the next level, we don’t have means of transport so the community or the client have to look for their own means. We have been struggling with the community to put up Community Emergency Transport System, because the community is wide apart, it is very difficult to bring them together to institute CET System. Hence, during referrals we wait for several hours before they are able to get means of transport to the next level of maternal healthcare. We do not also have laboratory services in the entire district so for us to conduct the routine laboratory investigations, we refer expectant mothers to Nadowli or Wa which becomes a challenge for many expectant mothers. Even for pregnant women to get money to look for means of transport to the next level of care is always a problem.

**Is there ambulance service for emergency referrals of expectant mothers?**

No, we don’t have ambulance or car in the district for referrals. If we are to send someone to a referral facility and it is around 10 am, the client cannot get means because all the vehicles go to Wa. Unless in the evening that they will come back.

**What is the dominant means of transport during emergency referrals of expectant mothers from your facility?**

Sometimes, they use motorking for some of the referrals.

***How are the outcomes of births referred by motorking?***

Some have livebirths, the unlucky ones have stillbirths.

***Do you have a pit for placenta disposal?***

Formally, we gave it to the pregnant woman’s relatives to go and bury and with our safe motherhood protocol, we are not supposed to give it to them, for the safety of the client. So we are using our toilet to dispose the placenta.

***Does it have effect on users of the toilet facility?***

Yes, because where we are disposing the placenta we don’t use there anymore. We are using the female toilet for the placenta pit whilst both males and females now use the same toilet.

**What are the issues you face in conducting childbirth in your facility?**

We don’t have enough space for the health centre so we are using the whole maternity block for the facility. therefore, we have partitioned the labour room into two: for labour and lying-in. but we are hoping Government will provide a place for the district health administration so we can use the old health centre building as well, whilst this place will remain as maternity only.

Secondly, I am the only midwife. I am always stressed up. Whenever I have two or three labour cases at the same, it’s always stressful form me working all the time. Also, if I am conducting ANC and a labour case is brought in, I suspend the ANC and attend to that one. Sometimes, expectant mothers default when it happens that way and it becomes difficult tracing them because I am alone. When

**I saw one bench, is it the same one used for ANC and Labour?**

No, we are having a porch for ANC. The bench you saw is the delivery room.

JANG YOUTH LEADERS

**BPCR means?**

Purchase of birth kits – soap, parazone, dettole, baby clothes, cloths for the mother, rubber bags. It also includes saving little money to support during referral. Expectant mother must ensure cloths and the other required items in birth kit are readily available with her at all times, especially when she is due. This will prevent unforeseen complications during prompt referral. A lot of issues are considered throughout the gestation. The nurses do carry out assessment of expectant mother’s family background to ascertain whether or there are peculiar sickness in the family lineage. The nurses further encourage the expectant mother to get blood donors available for emergencies. *There was shortage of blood in Nadowli hospital blood bank during my childbirth, so if I had not made preparations for donors, I could have died.* The nurses do add the contact details of expectant mother’s husband or family members for emergency purposes. All these are done prior to childbirth as part of preparedness for birth and complication readiness. BPCR aside the aforementioned by other participants, involves early uptake of ANC and ensure all recommended practices are followed. This will lead to good health and positive outcomes of the pregnancy.

**Safety of pregnancy?**

Safety of pregnancy is in two folds: either the foetus is dead or pregnancy is progressing well. If the baby is dead, it does not affect you until it begins to rot in you and so expectant mother might think all is well with her. Also, if recommended medicines are taken rightly by the pregnant woman, the pregnancy will also be safe. If she is regular to ANC, signs of no movement of the foetus will immediately be detected and extracted as early as possible. Therefore, pregnant women who do not experience baby movements or pains of pregnancy and fail to seek maternal healthcare might end up losing her life and that of the baby during childbirth.

**Support given to expectant mothers by family**

When a woman is pregnant, she is not supposed to engage in strenuous activities. We help them in cutting and conveying firewood home through organised labour by non-pregnant women in the section. They also use medium-sized water basins to draw water. They also partake in sowing but not beyond her strength. Expectant mothers are considered as sick person, so we exempt them from climbing trees. *When my wife was pregnant, I did most of the menial jobs for her such as getting firewood for household use, harvesting of dawadawa fruits, farming activities. If she was unable to carry any farm produce home, helped her to do them. I had petty quarrels with my wife when she conceived, but I stopped engaging in useless squabbles with her just to ensure she was safe. I did that to prevent high blood pressure (BP)in her. Some men even support in logging wood, cutting firewood and further support in brewing pito especially during the lean season. The loving men would cut and hire a truck to convey the wood home for brewing of pito and household consumption.*

***Madam, when you were pregnant for this baby, what were the support family gave you throughout the period?***

When I also got pregnant, my family gave me foodstuff to prepare meals. *For me whenever my wife conceived, I played with her stomach and herself, provided nutritious food items for her. The support family gave me was in different kinds. They supported me with foodstuff. Other food items that I longed for were also considered. Anytime, my appetite increased for a particular kind of meal, my husband provided and I experience deep comfort and sound mind.*

**What traditional reproductive sexual practices are in your community?**

They community practiced FGM in the past, but it’s been stopped now.

***But was it good or bad?***

Government have announced and warned categorically against FGM. We are aware this could be going silently in some pockets of the community, but for fear of facing prosecution we are unable to identify them.

**Suggestions**

We need more midwives in the health centre. If the health canter could be provided with ambulance for emergency referrals of pregnant women.

Many members do not keep the surroundings of their homes clean. Draining of the stagnant waters within houses could help prevent breeding of mosquitoes, which could further reduce infestation of pregnant women and newborns with malaria. Expectant mothers have shown laxity towards medical advice. Some do not attend ANC and or refuse to take recommended ANC medications given them freely.

Others refuse to report early to receive newborn healthcare, even after seeing signs of sickness in the baby. Women must therefore be educated on early seeking of appropriate maternal healthcare. The healthcare provision is dependent on ANC attendance. Women who fail to receive ANC suffer complications and birth of sick child.

Distribution of insecticides treated bed nets (ITNs) is a good policy by GHS. Of late, the nurses don’t give it to pregnant women until they in their ninth month. Others receive it after childbirth. We have to provide as family the ITNs and encourage expectant mothers and nursing mothers to sleep under them. the drainage from bathrooms should also be looked after because they are grounds for breeding of mosquitoes. The provision of food items is also a pertinent matter in Jang. Many young men impregnate their wives and run down to galamsey mining sites leaving her to the peril of nature. Starvation leaves some of them in unhealthy situation throughout the period of gestation until she is ready to conceive again for the next the baby before the men will return to them (women). Some women get depressed with suspicions and lack of love from husband for the entire period of gestation and postpartum.

Therefore, men who accompany their pregnant wives to clinic give them peace of mind and sense of love.

When they run down to the mining sites, who accompanies them to seek maternal healthcare?

Because the clinic is not far from the community, men do not accompany expectant mothers or nursing mothers to seek care. When it’s a critical health condition, any relative of the man supports her to the referral facility.

***ANC is not a market where husbands give them lift, he is part of the ANC***

*Yes, we aware, but I don’t go because of unforeseen financial expenses*

***I am aware ANC and the medicines provided are free***

Yes, they are free. Sometimes, you might get there and it’s not ANC. In that case, they do prescribe the medicine for us to buy. Some men have the notion that, since they are unable to continue to have sexual affairs with expectant mother, they feel their relationship has ceased for the period. This refusal to support women to seek maternal healthcare has been inherited from the elders. In the past, men were not involved in maternal healthcare seeking, which have been wrongfully emulated by the youth of today.

When community members see a young man attending ANC with the wife, they are tagged as “man-woman”. To prevent the name-calling, the youth deliberately refuse to support expectant mothers to seek maternal healthcare except during emergency referral. Men found expressing absolute love for their expectant mother or nursing mother perceive it as abnormality. For instance, when a man helps the wife to cut and convey firewood from the farm for his pregnant wife, received insults and castigations from recalcitrant men who saw him. The attitude of some expectant mothers is a factor to we not getting maximum family support. When expectant mothers are exempted from some activities which might affect our health, they refuse to resume their duties as woman after childbirth, thereby discouraging our husbands and family from rendering the needed support deserved. Some expectant mothers might even instruct the man in public to do a given a job at home. For example, giving the children a bath, but some expectant mothers perceive giving the children a wash is their husband’s duty, simply because the man assumed it completely during the period of pregnancy. *We also understand that, once they married us and took us under their roof, they have more post than us (women).*

JANG OPINION LEADERS

**Work women do when pregnant?**

***At home?***

Men do not do household chores, therefore, when women are pregnant, they still draw water for household use, wash clothes, giving the children a bath, cooking for the family. They also do cleaning of the house each morning.

***At work?***

When women conceive, we ask them not to indulge in work that is beyond them not to indulge in work that is beyond her strength. We encourage them not to carry heavy headload. I agree to previous contribution, however, in Jang, expectant mothers still partake in strenuous jobs such as charcoal burning, farm labour, pito brewing, wood logging, firewood cutting and conveying, because there are no alternatives to these activities. Poverty propels expectant mothers to indulge in hard jobs which at times affect the childbirth outcomes or lead to prolonged labours. It’s the same issue across the community. Usually, we know engaging expectant mothers in hard labours will affect their health and pregnancy outcomes, but we can’t do otherwise than allow them do it. We are all farmers in this community, and because we have no other source of farm labour for: seed sowing, harvesting of produce, fertilizer application, and other minor works, they are compelled to risk into engaging in them. therefore, we leave the outcome of the lives of pregnancy women to the fortunes of nature – if successful or not we accept it as such.

We have heard of the fact that, at certain stage of the pregnancy, expectant mothers should not engage in strenuous activities only that we have no alternatives than allowing than do all they could do as non-expectant mother.

**Do you record cases of miscarriages, still births and maternal deaths in Jang community?**

Yes, there has been instances where even at the hospital where we told expectant mothers gave birth, but suffered retained placenta leading to death. Some of such newborns do survive, other died.

***Aside the retained placenta, what other causes do you know about?***

I believe everyone has a destined way to die, therefore, when expectant mothers die in labour or after childbirth, we leave it unto God as the determiner of such death. There are some conceptions which expectant mother are often advised to stay off strenuous activities. Those who frown upon such pieces of advice from the midwife culminates in poor outcomes. Some will still engage in charcoal burning despite the risk of the fierce naked fires, and even pumping the borehole is not advised to do by expectant mothers, yet they still do it. But a lot of them do these jobs because families do not support them in doing household chores and exempting them from farming activities. Expectant mothers have this adage “living in hunger is death in itself” hence we will do all we can to eat.

We also have some girls who are below 18 years but willing to test “everything” adults do. Instances where such girls conceive, it results to one complication to another and many are unable to deliver normally. Minors are less developed to contain pregnancy, so most are unable to have normal childbirth resulting to CS births and the associated effects including loss of lives in the process. Jang is under Nadowli/Kaleo District but closer to Wa than Nadowli, therefore, my wife ones had a childbirth at home but was sick. Considering her health situation at the time, we found Wa hospital to be closer. However, she died before we reached the facility.

The major cause of maternal deaths are a result of the distance to relevant care facility. hence, during complications and emergency referrals, getting means to reach hospital becomes another challenge. Non-availability of means of transport further worsens the condition of the expectant mother, which often times lead to poor birth outcomes.

We have only one midwife for Jang and the all other six (6) neighbouring communities – Tibani, Zambogu, Kataa, Kyang, Janguasi, and Kanyiguase. It is a factor to some stillbirths and maternal deaths. It contributes to our delay in reaching referral centre. Whenever she is absent, we are compelled to go back home to prepare for Nadowli or Wa, meanwhile public transport do not connect these communities. When there is too much delay in getting means, they give birth at home or end up in stillbirth by the time they get to the hospital. other times, if it’s motorbike, then they give birth on the roadside. *Two of my daughter-in-law had stillbirths caused by midwife absenteeism. We rushed each of them to the health and were told she had gone to the district health administration, hence, the CHNs referred us to Kaleo health centre. When we arrived, labour was poor and it took each about days. When the baby came out, it only breathed shortly and died. We plead on government to post midwives to Jang HC.*

**BPCR means?**

When pregnant mothers register for ANC and commence the lessons, they are asked to begin to prepare for the items in their birth kit, so during childbirth, she will only have to just pick the kit along or send for it from home. BPCR also involve regular ANC attendance until childbirth. One important aspect is to arrange for means of transport to the referral facility during complications or childbirth. Therefore, when expectant mother progresses up to period of delivery, it’s essential for the husband to look for money with which to hire means of transport and purchase nutritious food items for her consumption in the postpartum period.

***You have mentioned means of transport as part of BPCR, what do you use to transport expectant mothers in complications and emergency referrals?***

During emergency referrals, everyone is ready to release his motorbike to transport her to the next level of care provision. We support in every way to save her life and the baby and probably ask for refund of money lend from the husband.

**What community initiatives are there for emergency referral?**

We don’t have any initiative as a community for emergency referrals and safe motherhood in general. Even money for emergencies, one has to move from one friend and relative to the other in search of money to borrow to be able to honour the referral and possible pay hospital bills. There is no ambulance at the clinic too. Hence, even if we save money, there is no car to rent aside these motorbikes and tricycle motorbikes.

**Community gatherings where MNH issues are discussed?**

We have men and women groups and other mixed sex ones for village savings and loans by WIDO - women integrated development organisation. Sometimes the nurses do come around to educate us on maternal and newborn healthcare. Recently, we had a meeting and the midwife came to educate us on the need for us to have community emergency transport system (CETS), only that we have not made any effort to implementing the initiative. But MNH care are not part of the essence for these weekly meetings. There two (2) irregular urvan buses plying Jang-Wa road. The midwife has encouraged the community to save money for emergencies and also liaise with the drivers to reduce the cost of hiring these vehicles during emergency and normal referrals to Wa or Nadowli Hospital.

**Do you have some expectant mothers not receiving antenatal care in this community?**

Yes, we have some like that. Normally some of those expectant mothers are encouraged to receive ANC but they refuse to go for ANC.

Others hide the conception from us as family heads. They normally do not want others to know they are pregnant. Many of the expectant mothers who fail to attend antenatal care are the unmarried ones and under aged girls and school children. They keep it secretly whilst sometimes devising illegal means of terminating the pregnancy. Even those expectant mothers who yield to pressure to receive ANC do it at the time when they are in their second trimester. When we mount pressure on them to receive maternal healthcare, because of shame from the community members, some sneak into galamsey mining sites with the pregnancy. The issue of midwife shortage and expectant mothers’ failure to receive care are not the only issues bordering poor pregnancy outcomes. Some men are ignorant about the relevance of maternal healthcare seeking. For instance, during rainy season, expectant mothers are stopped from going for care to being in the farm with the husband. Other men have the notion, “ANC can be received any day, and the moisture in the farm is available for few days, hence, let’s go to farm”. For fear of the partner, some expectant mothers comply with him and even sometimes receive few ANC visits before childbirth.

**Now that, men do stop the women from receiving maternal healthcare, are they involved in when expectant mothers are seeking care?**

We don’t go with women for maternal healthcare seeking. Even some men who appear rugged have been prevented by their wives from accompanying them to the health facility, because they she would feel embarrassed when her colleague pregnant women get to know the retched man is her partner. They usually don’t want other women to know a particular man is responsible for her conception.

**What are the causes of homebirths?**

Homebirths are not so common now as before because they know the dangers of prolonged labour at home. However, recalcitrant pregnant women still delay in reaching the facility which sometimes lead to roadside childbirth or even at home. They take late for the health facility. other times, when we delay in getting means to go Nadowli or Wa Hospital in the absence of the midwife, some end up having their birth at home.

**Suggestions**

What I will admonish colleague leaders here is for us to continue to propagate the message of attitudinal change towards maternal healthcare seeking in Jang community so we can further reduce the menace of stillbirths and stop maternal deaths completely. The fact that, we have not recorded one recently does mean we should sleep off to its existence.

What borders us most is means of transport during emergency. the case might be worse that she might not be able to sit on motorbike.

More nurses should also be posted to aid and further reduce the burden on us. Ready availability of essential medicines. we don’t have any pharmaceutical shop in the community or nearby but the midwifes do give prescription for us to buy. *How do we acquire those medicines within the given hour for treatment of the sick pregnant woman or the sick newborn?*

The labour room now has two delivery benches but we have just one midwife. So considering the catchment population, where there are more than one labour case at the same, she refers the unlucky ones to Nadowli or Wa hospital with all the vehicle challenges, which end up in poor outcome. Anytime the midwife is absent from the health centre, deliveries are not conducted until she returns.

***Traditional practices***

We have stopped traditional medicines. we try as much as we can to get the sick expectant mother or sick newborn to the health facility for treatment.

Jang health centre has a wider catchment population that all other facilities. The HC should be upgraded to a polyclinic to qualify for a resident medical doctor; it will alleviate the plight the community on MNH care seeking.

All laboratory investigations are conducted at Nadowli or the midwife liaises with Bill Laboratories in Wa to come to the health centre to take the samples of expectant mothers and transport them to Wa with all the risks and doubts with the outcome of the results. If the laboratory services could be provided for our facility, it will be very helpful. The staff is generally inadequate. Sometimes there are only two or three staff available in the facility. instances of severe pains in the client, it further worsens by the time she is given medication.

***Aside the rains or unwillingness of expectant mothers to receive care, what factors prevents men from getting involved in maternal healthcare*?**

We are aware the community is basically into subsistence farming, hence, whilst she goes for healthcare, the man will also be working on the farm so they can produce foodstuff for the family. Rainfalls are also seasonal, so it’s incumbent on men to work within the given time period.

***How about the lean season, are you duly involved in care seeking?***

Males involvement in maternal healthcare has not being practised by our parents and so we haven’t been acclimatised with walking or been on bike together with our wives. It looks awkward to pick her to the clinic if she is able to go by herself.

CHARIKPONG YOUTH

**Perception of BPCR?**

With BPCR, it means expectant mothers should go to the clinic for expert counsel and treatment of any pain or complications they may suffer. When a pregnant woman is prepared for birth, it means she has prepared her birth kit for childbirth and food stuff for her postpartum period. It also involves continued interaction with expectant mother by the family. This will enable the family to have updated knowledge of her progress or not so you she could seek medical attention early. The husbands should also support them pregnant women always throughout the period of gestation. Getting prepared for birth and complications readiness also means both partners must seek maternal healthcare together so counselling and STIs could be avoided throughout the period. The husbands must also monitor the medicine intake of the expectant mother and encourage her to take medications as prescribed, particularly with primes.

During childbirth, the couple are usually asked to purchase items for the birth kit – soap, parazone, cloths, Dettol, linen.

**Safe pregnancy?**

Pregnancy is said to be safe when expectant mother complies with all recommended advice by the nurses. Once she follows, the pregnancy will be safe. Some expectant mothers are put on specific diet to boost their immune system and or regulate their haemoglobin level, and these must be obeyed for the safety of her pregnancy and life. Some women are usually placed on fruits and vegetables diet but we have seen pregnant women rather drinking alcohol instead of eating nutritious food. The clinic further gives essential medicines to all expectant mothers, which some do not take. Women who do not take those medicines do not have safe pregnancies. Healthy conversation also promotes healthy and safe pregnancies. Once expectant mother is happy with the husband and maintains healthy communication at all time, it will give her peace of mind and ensures safety her conception.

**Support given pregnant women at home?**

The support given to expectant mothers is to ensure she eats recommended diets to boost her health. If she is put on special diet, the family supports by providing what they can afford. We also encourage and support them when they due to do strenuous jobs, such as cooking with large pots, burning of charcoal, pito brewing, and carrying of heavy load. When the tummy gets bigger, they are unable to bend down to even wash their clothes. Hence, some families wash their clothes and also try to stop them from farm labour.

**Work expectant mothers do?**

If she was into pito brewing for livelihood, they still do same activities when pregnant.

***So pregnant women still brew pito in your community?***

Yes, that is what they do for a living. Others burn and trade in charcoal. They also engage in farm work until such a time when her tummy does not allow her to bend down.

***On the average, in the ninth month of expectant mothers are they excluded from farm work?***

It’s not that they don’t go to farm when they due to deliver, but they usually do not do much work. Some families stop expectant mothers from farm labour until birth. However, expectant mothers with small tummy still partake in all farm work until they give birth. All activities non-pregnant women do, they are able to do them too.

**Community Initiative for emergency referral**

There is no community initiative for emergency referral to next level of care.

***Assuming expectant mother experiences birth pangs and or complications at odd hours, how are they transferred to referral facility?***

There are urvan buses plying the road to Wa. Therefore, the husband or the family liaises with the driver to help transfer expectant mother to Nadowli hospital. better still, some families use tricycle motorbikes. We understand expectant mothers experience excruciating pains when in complications or birth pains and should be given maximum comfort during referral, however, the available and affordabale means of transport is the tricycle though it shakes … wacu, wacu, wacu… up to Nadowli. *One pregnant women recently gave birth in the tricycle whilst on referral to Nadowli hospital. when she complained of birth pains, we contacted a public transport driver, but he was not forthcoming, so I used my tricycle to transfer her from Charikpong health centre to Nadowli hospital, but we could not reach the hospital before she gave birth in the motorbike. However, the midwife followed us on her motorbike, so she supervised the delivery in the tricycle.*

***Do you pick expectant mothers referred to Nadowli Hospital on motorbike?***

*Yes, we do. Others use tricycle or even a bicycle. Sometimes, we risk transferring them by motorbike but it’s a result of the high vehicular fares.*

***When do you pay for vehicular charges during emergency referrals of expectant mothers?***

*They usually tell us they need the money at the instant to top-up fuel to be able to start the engine. Meanwhile, we are aware most medicines at the hospital are not covered by the NHIS, so if we pay for the transport instantly, we will not be able to buy medicines prescribed for the treatment of the client referred. Therefore, we find alternative low-cost means of transport so we can buy essential medicines prescribed on arrival at referral facility. many treatments for expectant mothers apart from the ANC medicines are given on cash-and-carry system or prescription form is given for you to buy from chemical shops.*

***But I’m aware NHIS covers all treatments given?***

*No, that was then. Now, it only covers patient folder, the hospital bed when on admission and paracetamol tablets. Recently, I received prescription to buy medicines for my pregnant wife and infusions were inclusive.*

***Traditional medicines on maternal and newborn healthcare?***

We do belief in herbal medical practices as well as modern medicines. When a newborn and expectant mother fall sick, we do belief it is not an ailment modern medicine can cure. For instance, skin rashes, pimples and boils are believed to be caused by the “god of blacksmiths”, so the elders will have to pacify the gods and make a metal bangle for the patient’s wrists and feet for them to recover good health. White scalp on the skin is likened to our traditional cowries’ usage. Therefore, incantations and made on a perforated cowry and hanged on the neck of the sick person to be healed. Some families combine both modern medicines and traditional medicines. Others do not seek care from the hospital in such condition. It is also believed men must not see a neonate who is still less than 3 weeks old (for boys) and 4 four weeks old (for girls). Families who have joined such belief system prohibit it. However, men who have not been initiated into the belief system are free to see the neonate. Therefore when the husband wants to converse with the with, she has to leave the baby in her room and go out to meet him. Alternatively, she can cover the whole body of the baby so the man does not see the naked body. A break of the belief subjects the baby to perpetual and chronic sickness if not solved early; the newborn would die within few days. The man who sees the neonate will also become blind until the gods are pacified for him to recover sight. Therefore, men are not involved in postnatal due to this belief.

The women also have “bowl” and “calabash” beliefs. Usually, they are initiated into these for safety of the pregnancy and protection over the neonate. Hence, women who are into the belief system are not also allowed to see a neonate who is less than a month or even support expectant mother carry headload. The effects are that, the expectant mother who do not form part of the belief system could have stillbirth or the newborn could fall sick and if not detected early, the baby might die.

***Where did you get these beliefs?***

The belief and practices originated from our ancestors. They had gods to protect their families which these conditions. Therefore, all who inherited these idols must also go by the conditions thereof.

*In my view, these beliefs and practices are both good and bad. Sometimes we loss the baby but because it occurs at home we are unable to predict the cause – whether natural or the herbs. For example, convulsion has potent medicines at the clinic, but the old women will apply herbs until baby condition worsens before it is taken to the clinic. I do not like the beliefs on MNH care. If one is transferring the wife to clinic or hospital to give birth and the baby is due to come, he leaves the birthing mother in search for people who do not belong to the belief system to attend to the childbirth. Secondly, if successful delivered, the neonate will be clothed completely including the nostrils so the man does not see it. The last but dangerous and expensive option is to see the baby’s skin and immediately make the necessary pacifications to prevent the effects thereof. We also herbs – “naanyaa” women apply to babies to walk in less than nine months so they can conceive again or return to their income generating activities. They find “backing” the baby and when doing farm labour such as sowing or harvesting to be burdensome.*

***Where do you go for these traditional medicines?***

Many TBAs have these, but the TBA in at Dapuoh has a very powerful one.

***Does that mean, expectant mothers attend ANC and still receive care from TBAs?***

Yes, sometimes, the midwife is unable to reposition the well. However, when we go to TBA, she is able to identify the problem and risks and provide treatment for it. For instance, when we attend clinic with breech presentation, the midwife is unable to correct it. *Other women go to TBAs for herbs to ensure fast childbirth during labour. The herb is “mansugo” i.e. “cold” or “hot” local oxytocin*. Hence, if expectant mother mistakenly take the “hot” local oxytocin, it will lead to preterm or stillbirth or even terminate the pregnancy.

**During emergency referral of expectant mothers, do the midwife accompany you?**

*She does accompany the case to the hospital, but not always. When initial assessment finds the pregnant woman’s condition to be complicated, they follow up. In addition, if the transfer is by motor king, they usually do not accompany the client.*

**Suggestions**

Medicines should be readily available at the HC to relieve us of prescriptions even after we have active NHIS subscriptions. Travelling to Nadowli hospital on the poor road for laboratory tests is huge burden on expectant mothers and families. If laboratory tests and scans could be conducted in our HC, it will be very helpful. The TBAs should have also been trained to provide maternal healthcare together with the midwives. As community members*, TBAs could also carried out family cantered education on risks factors on pregnancy. It will help reduce the complex complications of pregnancies. Some nurses do not understand our language and dialect, so they are unable to provide required health education and lessons to pregnant women. Introduction of bed rest will also be efficacious for pregnancy outcomes in the community.* Pregnant women who are high risk could always go on bed rests pending childbirth. However, because expectant mothers only receive “surface” palpations, it is not enough to safe delivery. We believe is one cause of many referrals to the district hospital – Nadowli. We are aware bed rests ensure intensive care leading to safe childbirth. Providing the community with ambulance services will relieve families and pregnant women of difficulties during referrals. *We need education on attitudinal change towards pregnant women. Some men are so inhuman towards expectant mothers. Expectant mothers are left engage in high-risk to pregnancy activities such as pito brewing in order to save towards childbirth whilst some men do not support in anyway even when she is on referral to the next level. Lazy men even depend on their expectant mothers for livelihood, and could even complain if “more spices are not in the soup served”.*

***What education would you suggest be given to men in Charikpong community?***

We want the men to provide for expectant mothers throughout the gestation and support them to seek relevant maternal healthcare. Men should also be involved in ANC and other maternal and newborn healthcare. *Men still hide and give expectant mothers slaps on their cheeks. We want such men to know we are in modern Ghana now. A mind-blowing observation I made is that, some expectant mothers are factors to men not supporting them to seek care. Pregnant women are able to ride bicycles to funerals, even when the husbands complains of her condition. Therefore, when they want to attend ANC, we expect them to ride the bicycle again.*

DON NADOWLI/KALEO DISTRICT

**What are the arrangements the district office have put in place for pregnancy and childbirth referrals?**

We are limited in terms of ambulance services. Therefore, to connect communities to referral facilities during emergencies, we have instituted community emergency transport systems. We urge all community opinion leaders to contribute monies so in case of any emergency, they can borrow some it to hire transport. Some communities have begun saving whilst some difficult communities are yet to start. We hope and believe with continuous education, all communities will save towards referrals.

**Does the national ambulance provide services to your district?**

The NAS is a challenge because; the car in Nadowli has broken down. So usually, when the health facilities have a case, they call on Jirapa Ambulance Service to come and send the case to Wa hospital. Nadowli Hospital ambulance is not also effective; it breaks down most often.

**During my survey of the health facilities, I realised some expectant mothers still do not receive ANC or fail to complete it. What can you say about that?**

With pregnant women not starting ANC early, when we interviewed some of them, they explain the cultural beliefs and rites on pregnancy was the cause. In some communities, when a woman is pregnant, they have to perform certain rites before they can start receiving maternal healthcare or before other community members get to know she is pregnant. The rites are done before it is known to everyone, else, they belief she might lose the pregnancy. It is just due to ignorance, they do not understand the importance of early ANC uptake. Others too, it is due to the distance from the health facilities that prevent them from early ANC registration and attendance.

**Concerning the distance, I understand the CHPS compounds do provide MNH care**

Yes, they do provide MNH care. Occasionally, expectant mothers come to the midwife if their CHPS zone do not have a midwife to provide skilled assessments. Some mothers too still prefer going to the midwife to receiving care from CHNs/CHOs. Usually, when they get to know the CHO is not a trained midwife, they doubt her services provided. It is just ignorance, because, at present all the CHOS have been taken through basic ANC services, which they can provide.

**Research have shown expectant mothers abused or their integrity is compromised in providing duty of care which discouraging pregnant women from receiving maternal healthcare. What are your views to that?**

Well, we are human beings and our characters are not the same. All the same, we have trained all our nurses and midwives on customer care. Therefore, I cannot say all of them are behaving well, because in every home we have bad nuts. I think now, with the education going on. Many of our staff have changed their attitude – like, shouting and insulting clients. Except that, probably, those who go to deliver, you know at a stage in labour if the women doesn’t push hard for the baby to come out, there is possibility of the baby dying. So at that point, if expectant mother is not trying to push hard, some of the midwives shout on them to push.

***Are those shouts normal?***

Yes, they are actually normal. It is just to save her life as well as the baby.

**I understand from the survey, midwives have challenges from logistics to finances. But I know as the head of the district, you will understand these challenges better, which affect healthcare delivery at ANC, labour and postnatal**

We have so many maternal and newborn programmes that require motorbikes, but we do not have enough for all the facilities. Except the bikes, some of the roads are hard-to-reach. I can say, some staff do not also take good care of the logistics given them. All these are further worsened when the country’s medical stores were burned some time ago. As at now, even at the regional medical stores, some logistics are not there, compelling some staff to buy from private stores to use at the health facilities. It is a serious challenge with logistics to us, though we are doing our best to support the activities.

**Aside, logistics what other challenges do you face in providing MNH care?**

Some challenges include; the women themselves do not compromise with healthcare provision. For instance, ANC registration especially in the first trimester because of the cultural beliefs. We also discovered many men do not support their wives. Therefore when a woman comes for care and after assessing her, the midwife realise she has to be referred for further investigation and management, some of the men do not support pregnant women. Whenever we organise durbars at the community level, its only the women who attend. You will not see the men. It is a major challenge in the district.

**Do you have enough midwives readily available to health facilities for MNH care?**

It is better now than in the past; some CHPS zones even have midwives. We are still not enough as desired, because some midwives at a point attend funerals or other pressing issues leaving the facility. When clients go for care during midwife’s absence, the CHNs and ENs have to refer.

**What are your suggestions for improved MNH care in the rural areas?**

I think we need the support and involvement of the men in maternal healthcare. If men were supporting their wives, I do not think we will be having poor outcomes of pregnancies. Poverty among our people is also a major issue. Sometimes, expectant mother is referred to Wa hospital and because of feeding, the medicines, and others, they find it a big challenge so they don’t go. Some do not honour referrals because of inability to afford these basic things. I still do not know what we can do to help our mothers, maybe if the district assembly can support women to take up some small-scale businesses, it can help improve care seeking.

To add, we are doing our possible best to educate all communities on the importance of reproductive and child health issues. We are engaging all stakeholders such as the District Assembly, NGOS and other CSOs interested in improved MNH care. Some communities are being supported in way. We have Women Integrated Development Organisation (WIDO) supporting with VSLA to provide micro loans to communities towards economic empowerment.

OTHER NURSES 4

**Concerning emergency referrals of expectant mothers, what are the arragments thehospiital has put in place to ensure effective and efficient referrals?**

When it is an emergency case which have to be transferred to different hospital, we have an ambulance that pick the client to referral facility.

***Supposing, the OPD nurse call the ambulance and fail to reach them, what other alternative do you use?***

We have other facilities like Jirapa Hospital and Nandom hospital contact numbers. Therefore, if the ambulance at Nadowli town and the hospital do not function, we reach out to these other hospitals for help.

Do you have specific staff at the hospital who manage arrangements for ambulance or other alternative ways of reaching the next referral level?

The various units in the hospital manages the arrangements for ambulance. But there is mobile phone at the OPD which every unit uses for the arraignments. However, if Doctor is available at that moment, he writes the referral letter and then the nurse in-charge or on duty will call the facility for their help with the ambulance.

**I am aware you don’t work at the ANC unit, but you would have had some ideas on why some pregnant women refuse to attend ANC or fail to complete the course?**

Mostly, expectant mothers from the villages where public transport do not ply have difficulty attending ANC. They would have to walk to the nearby village with public transport before they can receive relevant care. Others do not have lorry fare to board trotro.

***Are men involved in maternal healthcare seeking?***

Some men are involved but many do not support their wives to receive care. Some men are very care that when their pregnant women are in a particular health situation, they do everything to help them. many men do not have time to attend ANC or seek care with pregnant women. Mostly, men who have more than one wife, do not have time to follow up. However, men who follow – up are those who have single wives.

**How are pregnant women treated during maternal healthcare provision?**

Some midwives are always welcoming to their clients and also take time out to explain anything health problem such as the laboratory reports, scans, and other preventive healthcare, and still even give expectant mothers the date for their next ANC visit. Nadowli hospital ANC unit midwives have been good, only that we don’t know how all treat expectant mothers. We have had cases where some pregnant women travel from the rural areas and Wa municipal to Nadowli or Jirapa hospitals for ANC, simply because of the proper attention given.

**If pregnant women prefer Nadowli and Jirapa, can I say they are poorly treated at their health facilities?**

It could be true, only that we can’t tell. I agree that “to every lie, there is some iota of truth”. There could be misbehaviours towards pregnant women in other places which management is not aware of. It’s not because I am a nurse, but I have been to ANC with other pregnant women in my two pregnancies and have witnessed the good attitudes the midwives put up at Nadowli ANC unit.

**Do you think the integrity of expectant mothers are compromised providing maternal healthcare?**

There is respect and dignity in care prosivion/ in terms of privacy, expectant mothers sit at one place and then care is provided in turns in the ANC room. The midwives assesses and counsels them individually in that room. The midwife would provide care in the room quietly and then pregnant will come out without anyone knowing what happened inside there. It is embarasement I cannot say much, but I was told, in one of the facilities, an expectant mother was told labour had not commenced and she went to the facility. so the expectant mother was send away and on her way home, she gave birth. However, when she went back to the facility for assessment and baby immunisation, the nurses insulted her for given birth outside the facility.

***Has it happened to you or someone in your presence or a case you have clear knowledge of?***

For me, no. I have never been to ANC to witness any form of maltreatment or embarrassment. Human beings are not all the same. Probably, some midwifes might be rude towards clients, though it is a delicate issue they deal with – two lives involved, the mother and baby, and moreso, nurses are public figure, therefore we try to be calm to all clients.

**I am aware of the key things at ANC is BPCR, what is your perception about this?**

In the first three months of gestation, fi happen to attend ANC, expectant mothers are welcomed and primes are especially invited to be part of the family of ANC seekers. This is done to establish good rapport so they would come for next visit. The midwife will then explain to expectant mothers basic habits and lifestyles during the period – what to keep with you whenever you moving out of home. As pregnant woman, at any point in time, ypu can get miscarriage or something can happen. Therefore, all pregnant women are expected by the midiwifes to move few cloths and clean rags. They further encourage all expectant mothers to carry their ANC card and NHIS card with the, for the sake of emergency. As pregnancy progresses to second trimester,say six months, the midwives expect pregnant women to carry along about four or more cloths and some more clean linen. You would be aske to buy blade, new thread, polythene bag, and other polythenes they use in spreading during labour.

**Why are expectant mothers’ aske to provide all those rubber spread, instead obtaining them from the health facilities**?

It is the individual expectant mother who provide all those items.

What if one is unable to provide?

They are not usually costly, however, during emregencies, the health facility will provide some of the tiems. We carry thesebecause you might not get to the facility before giving brith. So for expectant mother to be on the bear floor is not proper, one reason we carry those items along.

Do the midwives provide one-on-one conselling to expectnant mothers outside the laboratory reports?

Yes, earlier I said when prengnt women receive lab reports and have been explained to what they mean, the midwife normally take them through so many things that have to be done to ensure safety of the pregnancy and herself throughout thte period of gestation. If the expectant mother has an issue to discuss, it is at that point she is free to open up to the midwife for expert counsel. However, instances where the midwife is unable to address issues from an expectant mother, she refers them to doctor. If there are complications, she refers to the doctor. But minor issues are solved at the ANC level.

**Are there some challenges facing MNH provision especially ANC, labour and postnatal in the hospital?**

It is at ANC HIV/AIDS, IPT for malaria and other tests are conducted. Sometimes, the unit runs out of the tests kits. Instances, where there is shortages of tests, expectant mothers are asked to go and come another day. It discourages those from very far communities from receiving care again. Other times, the ANC unit do have problem with their weighing scale and other apparatuses. The weight is checked every time, to ensure pregnancy is advancing in accordance with the weight. When the scale fails to function, midwives go round other units in the hospital to find one, thereby delaying the entire process.

**Suggestions for improved MNH care**

I will suggestthat they should still encourage young women to go into midfiwery training so they can come out with new skills and ideas to help improve care provision. The public belief old midives have more experience in the job, however, it also have effect in case of inadequate midwives like our hospital. so they are unable to work under stress. Some are also compelled to be on contract after they have retired, simply because there are no enough midwives to take up the job. I believe midwifery profession should not be done by retirees because, some do not have good sight again and can make avoidable mistakes during childbirth.

OTHER NURSES 5

**At your worksite, we aware that some complications or normal childbirths are referred to other healthcare facilities for skilled care. What arrangements are put in place for emergency referrals of such expectant mothers?**

At the antenatal care, when expectant mothers go for maternal care, the midwife normally study the laboratory investigations to find out whether the client has a problem with any of the core indicators which may affect safe pregnancy and delivery. One of the key areas is, midwives try to find check on the laboratory report for sickling status of the mother. In case it is positive, we begin to look at the possible interventions to ensure safety of the pregnancy and her life. Then we also check on their G6PD (G6PD deficiency is an inherited condition in which the body doesn't have enough of the enzyme glucose-6-phosphate dehydrogenase, or G6PD, which helps red blood cells (RBCs) function normally. This deficiency can cause haemolytic anaemia, usually after exposure to certain medications, foods, or even infections*)* in order for them to keep intake of sulphur drugs. Any drug that contain sulphur we prevent such client from taking it. We further carry out haemoglobin test to know whether the client is anaemic and if possible the measures to take for them to build on their haemoglobin level. A lot of laboratory investigations are conducted for them. They are given health education particularly on herbal medicines intake with emphasis on local oxytocin they accustomed to taking. We stress it because, pregnant women belief when labour sets in and they quickly take it, it facilitate the progress of labour fro them to deliver fast and rest. Hence, midwives let pregnant women understand local oxytocin bring about vigorous or frequent contraction with poor cervical dilation which may result to CS delivery. They are educated more on these negative practices and beliefs on conception. They are further educated on nutrition. On BPCR, we ask expectant mothers to arrange for blood donors so they could donate when needed during delivery. Expectant mothers are further encouraged to save money for any emergency during childbirth.

**In terms of referral, has the facility got measures for emergency referrals of expectant mothers during complications or childbirth?**

With referral, the hospital had ambulance for referring patinets including expectant mothers. The ambulance covered the cost of expectant mothers who have subscribed to NHIS. But oflate, the ambulance is not on road. We the National Ambulance Service, but it’s very costly for the poor to afford, and it doesn’t consider NHIS subscritption. Even with that, the Naodwli/Kaleo District Ambulance (the NAS) is also not mobile. So NAS gave other neighbouring districts NAS contact numbers for the hospital to laisise with during emergency referral of expectant mothers. Some clients have self-arranged means of transport whilst others hire vehicles when referred to the next level of care. However, a nurse accompanies the referred client in the same vehicle to the referral facility.

**Now that that hospital does not have an ambulance and the NAS is not working currently, assuming a labour case requires referral, how is the hospital able to carry out swift referral?**

In such cases, the hospital sacrifice and use the official vehicle when the health condition is critical. The NAS is for the whole of Upper West Region, even though, in every district except Daffiama/Bussie/Issa and some newly created ones, some maybe called to support other districts in time of need. The NAS generally is not reliable for expectant mothers. Because, during emergency referrals, they might have been to another district to assist in accident cases or health center/hospital -to – hospital referral.

*When office vehicle is used, who pay for the cost of referral?*

It is the client that foot the bill.

**What prevents expectant mothers from receiving maternal healthcare such as ANC or fail to complete the full course?**

In this part of the region, many expectant mothers and families are not enlightened, so they don’t know the importance of ANC. Some even attend ANC ones and never receive care until they givebirth at home. Others register and take the card but refuse to attend regularly for the medicines. midwives are compelled to follow-up. I do not know the exact reason why some refuse or fail to complete the course, but I have heard pregnant women complain about the distance and means of transport. Due to shortage of midwives, expectant mothers can spend the full day seeking care, which discourage the very distant communities from seeking care. However, I believe, it is due to the limited knowledge of ANC as the reason behind the poor attendance to maternal healthcare services.

**Why are men not involved in maternal healthcare seeking with their wives?**

*During my student clinical in one of the communities, a man walked in with the pregnant wife and the midwife at the time asked him why he has been regular to ANC with the wife and the response was, anytime he attended ANC, the community leaders called him and questioned him on his regular follow-up to ANC. The man in question was told it is not practiced in the community. “you want to spoil our wome for us because ones other women see you going each time with your wife, they would also bend on us to do same”- the man added. Therefore, eventually, the man realised it was a serious threat to his life in the community. So he stopped.* however, at the hospital level where I have worked, I have never seen the hospital management calling on community leaders to conscientize them male involvenebt in maternal healthcare, particularly on birth preparedness and complications readiness. It is only the women who attend ANC, so we educate, counsel, and guide them as to how they can keep themselves to have safe pregnancy and childbirth.

**With BPCR, what are the issues midwifes cover during ANC?**

When pregnant women register for ANC, they are encouraged to bring family or community members to donate blood so it can be stored at the blood bank pending any emergency. expectant mothers are also aske dot save money towards emergency referrals, complications and normal childbirth. They are further asked to purchase cloths, baby clothes, and clean rags and keep with them at all times for the sake of emergency. alternatively, she could keep the birth kit within the reach of family members so in case of emergency, they can pick it up for her. Expectant mothers are encouraged to take their NHIS card with at all times so no cost will be involved during delivery. The fifth aspect of the BPCR education is to walk along with their ANC card, so can receive care at any healthcare at any nearby facility. the card will inform the nurses of the kind of conception and the health condition of the pregnant women so appropriate care can be provided. During, the ANC information in the card about the pregnant women is relevant to ensuring safety of client and baby. It provides information about the necessary steps required during labour, and whether it is a high risk pregnancy.

**Do you think clients social and physical integrity are compromised in providing duty of care to expectant mothers?**

So far that I have worked and in my working experience, let me say they are a lot of challenges. Challenges from; *the client and we the healthcare providers.*

***What are the challenges from the clients’ side?***

A lot, number one, some pregnant women come without the required rags or clothes for themselves and the unborn baby. Some of them, you deliver them and where to even get something to just cover her is a problem. Others come when asked to buy an important and urgent item, they tell you they don’t have money. So will what will the midwife on duty do? Are you going to pay for them or what? Assuming a client arrives and already in the second stage, you immediately send her to the labour ward and realise the bay is coming and you turn around to find there is nothing to cover the baby when delivered. Sometimes, there are no cloths, linen, rags, just nothing to use to receive the baby. In such case, what will the midwife do? Even after delivery, we ask them to buy certain food items so they can regain energy fast, but they will tell you they don’t have money. And they will end up collapsing on you because of body weakness.

***What are the challenges on the side of the nurses?***

Our communication to expectant mothers are sometimes not good and are either intentional or unintentional. It is due to pressure form the work. For some midwives, it is the pressure. There is inadequacy of midwives, thereby putting so much pressure on the few. When we are tired, any least thing the expectant mother does, it irritates the midwife. Other times too we are forcedto say something which is not pleasant to clients. However, before we notice it, it is we have divulged it already.

***Do you shout or yell at expectant mothers?***

Sometimes we do. We shout at them sometimes under pressure. But their lack of resources for childbirth do usually contribute to some of the poor attitudes towards them. assuming a client si delivered and need oxygen and there is no oxygen at the labour room at that instant, it is one cylinder in the entire hospital, so the lone midwife is compelled to leave the client and go to different ward. So by the time the midwife returns, something would have been wrong with the client. Management of the facility do not also motivate staff to deliver optimally. The up and down movement to safe lives makes midwives very tired that, any little thing from expectant may call for insults, unintentionally.

***I understand your code of ethics is against you shouting at clients***

I said early on it is not supposed to be that way but because we are stressed up for inadequate staff. Imagine a midwife goes for morning duty for eight to nine hours, and probably do not get a reliever, you are forced to remain on duty even if it is another eight hours. Sometimes, the reliever may come to takeover at the time there are many labour cases, so you have to remain to conduct all those deliveries before going home. we are human beings, therefore even weariness psychologically affects your performance when conducting deliveries. When the mind is stressed up, it is difficult to handle a labour case. We also lack resources to achieve set targets of providing relevant care to expectant mothers. Sometimes, when you are conducting a labour case which require blood transfusion, you could call the laboratory and know body will mind you. So you have to leave the client and rush to the laboratory for infusion. If midwives are not many on duty supporting each other, it becomes a serious challenge providing care to all clients at the same as expected.

***I know usually, midwives and non-midwives are put on duty at the maternity wards. What is the case at your facility?***

At Nadowli hospital, they are only midwives. The non-midwives are just one or two and they are not ready to support during complications. The management of the facility do not allow non-midwives to handle obstetric cases. Therefore, non-midwives have made up their minds not to attend to any case of obstetric related. So even if you come on duty with them, they are only going to take vital signs and others that are not on obstetrics related.

**What are the challenges midwives face in carrying out their duty?**

Defaulters. Many expectant mothers default ANC attendance. So the midwives have to trace to their house. That is why when expectant mothers register for ANC, we take their house address. It is very difficult tracing them. some stay in very far communities, others even stay at Wa implying the midwife have to fare yourself to Wa to trace defaulted client. It is our duty to trace them. It is a major challenge faced in providing quality ANC to pregnant women. Women who come for ANC and are found to be sickling positive, HB positive, Hepatitis B positive, all those people need to go through further laboratory investigations.

***Why further laboratory investigations?***

They are the high risks expectant mothers. When we ask them to go through further investigations, they refuse because they have no money. All these issues can even drive the client away because she knows if she visits ANC next time, the midwives will ask for them why they failed to do the lab tests. all those embarrassments may be there. So they will not even attend ANC until they are in labour. It is a big challenge for midwives because if anything poor happens to the pregnant woman, management will demand your intervention which you will have nothing to proof or defend the case.

Men are not also involved in providing maternal healthcare. Because, we all know that pregnancy involves two parties. We have stressed men involvement but they donot come. We have aske dthe women to attend ANC with their husbands, especially pregnant women who are tested STIs positive so we can treat both and counsel them, but they would not come. Pregnant women with STIs will come back to tell us the men said they don’t have any sickness.

***What happens to the infested expectant mothers? Do you treat them?***

Yes, we treat them, but you cant stop the husbands from infesting them again. Sometimes, we keep treating STIs on particular women until they give birth.

***How about women in labour, what are the challenges midwives face in providing care?***

So many challenges. Some pregnant women come to the hospital alone. No family member accompanies them. they will come from morning until evening with nothing for them to eat. During labour, we may need a relative to support in providing care such as picking the woman’s items from the birth kit and there is not relative to do it. Other times, we may need relatives to sign consent form, donate blood, and there is no relative to do any of these.

***On the aspect of the equipment, what are the challenges there too? Are they up as the highest referral facility in the two districts?***

Currently, for the labour ward we have only three beds, out of which only two are used. The other one is used only when there is no other option. It is not meant for childbirth. It is for examination. In critical situations, we forced go conduct delivery in the (manual vacuum aspiration) MVA room to conduct delivery. I have used the MVA room to conduct childbirth ones, which is wrong practice. The room is meant for expectant mothers who attend the hospital with incomplete abortions, so they are send there for EOU (to evacuate the uterus). MVA room is meant to evacuate the uterus, examination of the uterus and related cases, just removal of uterine content. So as for the resources, it is very poor. We don’t have delivery beds, resuscitation table, a lot. We lack a lot of things there.

***However, do you conduct resuscitation currently?***

We do resuscitation, that is why I’m saying there is supposed to be resuscitation table. The table require a lot of items so that in case a baby is born and is asphyxiated and need immediate intervention, use conduct it with ease. The table we have now is not well-equipped. We are only managing to save lives.

***During postnatal care, what are the challenges?***

Some of the mothers’ refuse to come for follow-up postnatal care. For the first postnatal, once they come to the facility to give birth, we administer the first PNC to them. Provided they give birth at the facility, many of them receive PNC before they discharged. The first one is within 24 hours of birth. Then the second is seven days, third PNC is 6 weeks’ time. But PNC, many receive care because they know the baby will get vaccinated. As the mother go they will be weighing and giving them vaccine. It is much of a challenge with PNC as in ANC.

***Are the vaccines readily available?***

Yes, as for the vaccine, they are always there. Hardly have I heard that there is shortage of vaccine.

***What recommendations will make for improved maternal and newborn healthcare outcomes in rural areas?***

Providing vehicle for referrals is number one priority. We lack vehicles for referrals of emergencies. Sometimes, we have a case and before it leaves the facility, we suffer a lot before the client get to the next level of care.

Inadequate skilled staff. We don’t have enough skilled staff. So we don’t meet the expectation of the clients. As I said early on, we don’t explain certain issues clearly for clients to understand because we have limited time to carry out all education and detail explanations to expectant mothers.

Lack of resources. Delivery materials -beds, oxygen, a lot are not adequate. Those items are needed to meet the needs of pregnant women and the unborn baby.

***Do you have all necessary personnel – laboratory staff, doctors and anaesthetics as required?***

No, they are not enough. Sometimes, it is only two anathestics that are in the hosptiatl. Sometimes, one will be on leave leaving only one. We could call the contact of the anathetics and it is switched off. Other times, he will tell us he is very far away and if the client is an emergency case, what are we going to do. The doctors too are sometimes few, maybe the doctor is gone on official duty and very far away from the hospital. we are forced to refer the client out of the hospital or may be throughout the day and night, the doctor might have worked so hard that if h tries to attend to the next case, the outcome may be bad. So it is referred out of the facility.

WOGGU NON-PREGNANT WOMEN

**Do you still experience maternal deaths in Woggu community?**

Yes, but it is not of recent. We still have women who experience severe complications and prolonged labours but it doesn’t always to death as in the past two and three years. Still births are rather very common now.

**What are the causes of the still births?**

Sometimes, after the child is born they fall sick and when taken to the clinic, we are told of the cause of the death. We are not usually told of the name of the sickness, thereby making it difficult for us to take precautionary measures.

***Now, that you are told of the causes of the still births, how will prevent future occurrences of stillbirths in the community?***

Normally, such sicknesses do not keep the baby long. Usually, when it happens and its rushed to the clinic, but they are often unable to treat it. Sometime, we assume its convulsion. As for the maternal deaths, its been long experienced it. Even homebirths has been discouraged among the young ladies. Recentoy we had some deaths of baies and the nurses sadi it was caused by CSM. We know CSM infects people when we are in the warm season, but it was still in always very cold yet the deaths were attributed to CSM.

***Was it so rampant?***

Yes, it was very common. They occurred in the early part 2016.

**What work do women do when they pregnant?**

Expectant mothers do the same jobs as when they were not pregnant. They go to farm and partake in all activities as before conception.

***Can you mention the activities?***

As I sit, I brew pito, so when I conceive I still brew pito although the heat form the fire could affect my health. If I don’t brew pito, I won’t get money to grind flour, buy salt, and other food ingredients. Although I feel the pains and suffer the risks of the fire, I still brew, simply because I have no other option to make income. During the rainy season, pregnant women do sowing of seeds. Unlucky ones even till and weed with hoe whilst pregnant. So as we continue to engage in these menial jobs, usually it gets to a point where we experience severe pains and complications. However, we endure because it is the same work every woman whether pregnant or not. Expectant mothers only just endure the pains when working.

***What other activities, aside tilling, weeding, sowing of seeds and pito brewing?***

We also burn charcoal. I burnt charcoal throughout my recent pregnancy and suffered unusual pains during labour but was lucky to have a successful childbirth.

Some expectant mothers do climb trees during fruit harvesting -dawadawa fruits. They also climb during rainy season to cut dry wood/sticks for cooking. However, its not all pregnant women who do climb trees. Those with week umbilical cords do not attempt whenever they are pregnant. When a pregnant woman knows her cord is the weak type, she does not climb trees.

***How will one know her cord is weak or strong?***

Normally when a woman give birth to the first baby, the cord is checked to know whether is thick or lean. So adult women normally advise her not to ever climb tree in pregnancy. Young ladies do take risk climbing trees because they do not yet know their cords.

***How will adult women see the cord if the childbirth is at the clinic/hospital?***

These can only be known when it is a home birth. They take time to check it. If it is at the clinic, access is limited.

***Don’t you have anyone to climb and harvest the fruits for you?***

The men often tell us pregnancy is not a sickness and a fashionable thing for you to keep off doing things everyone does. Others tag such expectant mother a lazy person. Therefore, we feel better doing all activities by ourselves.

***Are you always aware, it could lead to the end of your life?***

Yes, but we always pray death do not happen.

I conceived for twins and engaged in all these activities. When I went for ANC, I was told the foetus was not moving, and I was always very uncomfortable and I also had short-breaths sometimes. But I endured unto birth. One reason we these jobs is because we are alone, with no support from family and husband. Failure to do those jobs means you may not even get food to eat. When you unfortunate to have a rival in the marriage, she feels you are shedding responsibilities and work on her to do. She might tag you as lazy and even cause you more pain by picking fight on small issues. We just have no alternative than to endure everything in order to avoid these. In our community, even if your husband has two wives, we are worse off in terms of supporting each other during pregnancies through vain rivalry.

**What are the things you do to ensure safety of pregnancy?**

When we are pregnant, the first thing we do is to register for ANC. Ones, you register, anytime you are in pain or complications the nurses give you relevant medicines. however, when you fail to take medications given, as some complain they might have overweighed babies at birth, giving complications and difficult labouring, but when we comply with the prescriptions, we receive are restored to good health fast. Right now, the nurses have admonished all pregnant women to keep off local oxytocin intake. We now rely solely on the clinic for our maternal healthcare. Many expectant mothers are also regular to ANC. Women who take full course of ANC medications and conduct laboratory investigations actually bring forth healthy babies. They look healthier and more active than women who refuse to take the medications given at ANC. Also, we are often told to sleep under insecticides treated bed nets (ITNs) during rainy season to prevent malaria in pregnancy. We ensure we receive ANC and further eat nutritious food. All these help us to have safe pregnancy and childbirths to healthy babies. Many women ensure hygiene of the newborns are maintained.

**Does this mean some women do not take the free medicines given at ANC?**

Yes, some take it home and throw them away. Others complain their unborn baby will increase in weight at labour, which might lead to difficulty in having normal childbirth. For fear of CS delivery, many refuse to take the medicines. we have pregnant women who complain of vomiting anytime they take it. Others feel dizzy after taking it. *I don’t take the medicines given. Anytime, I took it I vomited everything instantly.*

*I do not complete the dosage of the medications as required, due to negative reactions I experienced.*

**What traditional beliefs and practices do we have on MNH care?**

There is one herbal practice – “giving newborns a bath with Siram”. It’s done as soon as the baby is taken home from the clinic – thus if skilled birth. It is done to ensure good health of the baby. Women who been initiated into the herbal practice/belief must not see newborns of who do not belong the belief.

***Is it real, that seeing the baby could lead death?***

Yes, when I had my recent birth, one woman who is into this belief saw the baby and said the front fontanel had moved to the back of the baby, which was the effect of the belief, so I had to immediately get the baby bathed with the herbs and the front fontanel was restored.

***When you are attending PNC, how do you prevent such women from seeing your uninitiated baby?***

We cover the entire body of the baby before leaving home. “*This belief and practice has killed two of my neonates”.*

***How do you know that was the cause?***

*The child’s faecal matter looks greenish in colour.*

***Did you send your late newborns to the clinic or hospital?***

When we lived was very far from a health facility. it happened before we packed out of the community and returned home.

We have herbs we boil into syrup and administer orally unto the baby. We are able to decipher sickness that can be treated medically and those that are traditional.

Even when we give birth at the health facility, we are eager to get home quick to administer the herbs before the baby dies. We belief that, women who are into it might have come to visit at the hospital or clinic after childbirth. Once they see the newborn, it’s infested already with the sickness and until the nursing mother and the family act promptly, the baby might begin to show signs of poor health, which could lead to baby’s death. The hospitals and clinics are unable to treat such.

***Why don’t the nurses’ belief and practice these, yet their babies are alive and healthy?***

we belief they also hide and practice it without letting us know. They all do it, I belief.

**Why do some pregnant women not give birth at the health facility?**

Childbirth is such that, it can happen at home or anywhere. Sometimes, pregnant women give birth on their way to the clinic. Particularly women with precipitate labour. It is women who experience epidural labour that are reassured of reaching to the facility before childbirth. My last homebirth, I felt the pains but I didn’t have water so I took a bucket and dashed to the borehole to draw water before going to the clinic. When I arrived home with the water, I send for my husband who was at a relative’s house closed by. I send for my neighbour (an adult woman) also, to come accompany me to the clinic. Before they both reached my house, the baby was already out. There is a prize for women who give birth at the clinic. Therefore, when I went to the facility after the childbirth for further examiniation and immunisations, the midwife queried me for not making it to the facility after in had made seven antenatal visits.

***I guessed it was the headload you carried?***

No, the water bucket was very small and was not even full.

***Anytime, you are pregnant and in the birth month, do you still carry headload including water?***

Yes, until we deliver. My twins, I carried headload of firewood on the same day before I went to the clinic to give birth to them. The men are poor, so we hustle to augment their efforts.

**I was told this morning, you do have weekly group meetings at your various sections, do they talk to you about MNH care?**

No, nobody has come to talk to us about MNH care. It is not also part of our usual agenda.

**Are there community initiative for emergency referrals of pregnant women?**

No, if your husband is unable to arrange for means of transport, then you might suffer.

***Do you have savings pregnant women can borrow to seek care during emergency?***

No, it is just you and your husband who have to manage the entire emergency. also, if relatives are available during your husband’s absence, they will look for means to send you to Nadowli hospital. otherwise, the pregnant women will have to work out means of transport such as the motorking or a motorbike if she can sit in-between two people to reach referral centre. As a community, there is no initiative for pregnancy referrals. The midwife ones created awareness of the community through the opinion leaders on the need to save for emergencies referrals of pregnant women and newborns, though it has materialised yet.

**All of you have had childbirth experience, if somebody talked to you about BPCR, what do you think they might be talking about?**

BPCR is of two folds: preparedness for food stuff and money for referrals. To say I am preparing means I am saving money for any emergency occurrence as pregnant woman. When you have saved money, during complications, community members are willing to support you reach referral centre. However, if you have not made any efforts to save money towards emergency situations as expectant mother, they aware you will be a burden on them should they attempt to support to hospital. Even if they use their money to hire means of transport and also pay hospital bills, they are not sure of getting refund for their money, therefore, they are usually reluctant to support such expectant mothers. BPCR means purchasing all the items needed at childbirth – soap, blade, cloths, clean rags, thread, body powder and pomade, hair combs, washing powder and parazone or Dettol for nurses to wash their hands after conducting delivery.

You also need to prepare some essential food items before giving birth, so you don’t have to suffer to cook to meals or when the childbirth is at a referral facility. you may have no relative available to cook for you. Therefore, you must prepare all these in advance pending labour. Every pregnant woman must also ensure you carry an active NHIS subscription card. It is another important aspect of preparedness.

**What suggestions will you make for improved MNH care in your community?**

Government should come to our aid by getting appropriate medicines for the sicknesses we treat with herbs. It is affecting the health and lives of innocent women and babies. We have a serious challenge with portable water supply. Government should provide a borehole for the clinic and the community too. It is ANC and PNC women who draw water for the nurses. we compelled to carry water for them even as expectant mother during ANC visits. When we go without the water, the nurses will send you back home without providing care. We also need an ambulance for emergency referrals of pregnant women in particular. Many avoidable stillbirths are caused by the search for means of transport.

***How do you dispose placenta in your community?***

If child birth takes place at the clinic, it is given to the client. When I gave birth at the clinic they gave it to me.

***Do you bury it yourself?***

No, it is given to the men to dispose off. I threw mine into the public toilet facility.

***How are you treated during MNH care seeking?***

We are treated very fine. The only time we have issue with them is when you fail to comply with their counsel or fail to fetch water along for them.

JANG - NON-PREGNANT WOMEN

Do you record neonatal deaths in the community?

Yes, it still happens. In 2015, it was better but we have had a number of newborn deaths in 2016. They were very sudden.

**What caused those deaths?**

Anytime they are sick, we rushed them to the clinic and were referred to Wa hospital, they die. Some of them, do not reach the facility before dying. We are therefore unable to tell the cause. Supposing they received medical attention, they might have diagnosed it but none of those newborns received medical care before they death. However, it was rumoured to be an airborne disease though the name is unknown. Others said it was CSM.

**What are the causes of stillbirths in your community?**

Some of the stillbirths caused by the expectant mothers. When they attend ANC, they recommend certain food items and ingredients to them to take, but some refuse to comply to the advice given. Others say their mother didn’t eat those food supplements but had successful births, thereby refuse to take counsel. Many fail to understand that, the present times are different from the olden days.

In addition, when expectant mothers are given medications, on reaching home, they throw away the medicines. women who refuse to take ANC medicines mostly have stillbirths or deformed or malnourished baby. It’s true, others keep it in their bags but refuse to take it. ]poverty is another issue. When expectant mothers are asked to eat certain food items such as egg, regular meat, pineapple fruits, oranges, and other essential items, the men complain, it’s a trick to get what she wants. The men do not even if she needs them to boost her immune system or regain haemoglobin level.

**What work do expectant mothers do?**

In jang community, when women conceive, they do more work than the non-pregnant ones. They try to finish doing most of their work before they give birth, which time they wouldn’t be able to resume to active work early. Expectant mothers’ belief, when they give birth, they have rest from work for some months. They over-indulge in firewood cutting for use when they give birth and extensive pito brewing to save for birth kits. If it is shea nut gathering season (April-August), they stressed to gather much so they could process more butter and keep towards their birth. It is women who fend for themselves in this community. Some expectant mothers burn charcoal and convey it to market to sell. Therefore, pregnant women more work to cover for their early postpartum period. Pregnant women climb trees to pluck dawadawa fruits and firewood, fetching of water for household use, farm labour. *Some expectant mothers are even stronger in pregnancy than the postpartum.* Particularly, the intake of the medicines given at ANC give a lot of energy to work. Pregnant women who fail to take the medications are not as stronger as their counterparts who complete the dosage.

**BPCR means?**

It includes preparing the needed items such as clean rags, linen, cloths of yourself, baby clothes, baby dypers, soap, washing powder and some jewelleries to look attractive. Our preparation includes attending ANC regularly. If we fail to attend ANC regularly, childbirth could develop complications. Nurses are able to detect any risk early and treat it so pregnancy can be safe and safe birth outcomes to healthy baby.

**Do you have any community in titivate for emergency referrals of pregnant women and newborns?**

There is no initiative for emergency referrals in our community. When there is a case, it is the husband who will go about looking for money to hire transport to transfer the wife whilst make refund later as agreed with the lender.

**Do you have traditional beliefs and herbal practices?**

Yes, we do have. When a baby is sick and does not have active NHIS card, we resort to leaves and herbs for treatment. The NHIS card registration and renewal has premium payments involved, therefore, when we register, but the health facilities rather allow us to buy many of the essential and costly medicines from chemical shops. Therefore, we just buy the medicine direct from the shops or boil herbs and leaves for cure. Government said premiums for school children will be covered by the state. However, we still make those payments from wherever, before marginal refund is made to parents.

***Do expectant mothers administer local oxytocin?***

Yes, we still take it, particularly when ones NHIS card is not active. The medicines provided under the NHIS is unable to cure many sicknesses. It is pain reliever such as paracetamol tablets were provided when we sought healthcare. Even the babies are given the same medication. Hence, if we don’t combine traditional medicines with the medical treatment, the children will die. Normally, we subscribe to the NHIS because, we are unable to afford out-of-pocket payments for healthcare provision. But we realise that each time we go, they will prescribe a long list of medicines for us to and buy from chemical shops. Once, we don’t have money to buy, we apply the herbs and good health is restored. Recently, I send my baby who suffered measles to Wa hospital. I had not received treatment before a man rushed in with his sick child also. The said man did not have NHIS but expressed readiness to make out-of-payments for healthcare service. The nurses left my case and attended to him fully. When I complaint about the neglect, a long list of medicines was prescribed including infusions for me to go and buy. It was during a heavy down pour of rain. I went into the rain and purchased these medicines and luckily, the baby became well.

**Why do some expectant mothers refuse to go for ANC services?**

Some of them deliberately refuse to seek care. Many of such women end up on referral to Wa hospital during childbirth. Other women do not go because of the insults from the nurses. they receive all forms of insults. *I focused on TBAs ANC because, the clinic does not provide proper maternal healthcare to pregnant women. I have observed that, many of the women who patronised care from only the clinic end up in complications during childbirth and are referred to Wa hospital or Nadowli hospital.*

***How are expectant mothers’ treated ANC?***

They receive chucky words from the nurses, so some do not receive maternal healthcare at clinic because they have received enough of the insults.

***Why do expectant mothers conceal this attitude of the nurses towards them?***

They are afraid to divulge the truth for fear of receiving worse treatments in subsequent attendance. Worse of it, we could take a sick baby at odd hour to the health centre and the nurses would pretend not to know a case has been brought.

**Do we have homebirths in Jang?**

Yes, but it happens unexpectedly. Others give birth on their way to health facility. usually, when we feel the baby movement and begin experiencing labour pains and get to the clinic, we are told to go back and return when labour advances. She will complain we are not due to give birth. Therefore, with such treatment, we try to give birth at home to avoid further insults. The home births are caused by the behaviour of the nurses towards labouring women.

Do expectant mothers take ANC medications? Some do, others refuse. But I believe women who refuse such medications as ignorance. Before, ANC medicines were introduced, women gave birth to deformed babies, others had babies who took longer period to start walking. When we take it, we have healthy children and we get healthier fast after childbirth when full dosage is actually taken.

**What reproductive sexual practice do you have in this community?**

We practiced genital cutting, but we have been warned not to indulge in the female genital cutting anymore. Therefore, we have stopped although, it was helpful.

**Suggestions for MNH care?**

We want government to talk to the nurses on our behalf to treat every client with priority. When we seek care as a sick expectant mother or sick baby in the evening or night, they pinch us with words for seeking maternal/newborn care in the evening or night. Some would even tell us they don’t run shifts and so not work after 2pm. We as adults may be able to endure the pain until the next day, but the babies. So they should try to attend to the babies who are send at odd hours. if we are not lucky, we are send away to return the next day for care. I have not been to other clinics, but the nurses in Jang clinic do not us attention at all whenever we seek care at night or evening. They might not even utter a word to you, before going back into their residences. When we give them a knock on their doors to attend to emergency at night, they refuse to provide care, not even a pain reliever. When the client family insist, they just write referral letter for you. Given the odd hour, how are we going to manage the case to Wa or Nadowli hospital?

We further need support from to revive our savings group and institute CETS. The women groups have felt the need to relive expectant mothers and neonates during emergency referrals by forming a group to save money that anyone could borrow from and refund later.

Hygiene education and draining of stagnant waters to prevent malaria. We are aware of the dreaded effects of malaria infestation, the reason ITNs are given to expectant mothers. We need this education because, at Jang clinic, expectant mothers are not given ITNs until they are due to give birth or when they give birth before the ITNs are given to them.

We are told per the GHS/MoH protocol, to conduct malaria tests – Rapid Diagnostics Test (RDT) before given medication. Nevertheless, in our clinic anytime we seek care with the babies and ourselves, we don’t see them any sign of tests and yet medicines are prescribed. Usually they provide the cheap medicines at the health facility and write the expensive ones for us to buy- from Wa or Nadowli.  *I have realised, anytime I administer such medication, the condition of the child worsens compelling me to send the child to a hospital. so usually, I do not seek care at the clinic, I instead go to Nadowli or Wa hospitals without referral.*

RDT are carried out here ones in a while. Meanwhile, it is the dominant cause of many ailments in the community. I have also observed that, each time we seek care, whether ten or more sick persons, the same medications is given without conducting the RDT. Pregnant women should also be encouraged to do some exercise and reduce their involvement in menial jobs – particularly charcoal burning, tree climbing and pito brewing.

TBAs did not go to school, but were able to handle all cases, because we didn’t have midwives. Women were treated with respect too. But nurses receive training at school and yet unable to provide quality care to. The midwives and the CHNs/ENs need skills upgrading. It will help reduce the number of referrals of pregnant women and newborns in particular.

CHARIKPONG NON-PREGNANT WOMEN

**What are the causes of stillbirths?**

Stillbirths are caused by the refusal of expectant mothers to seek maternal healthcare at the clinic or hospital. once a pregnant women fail to receive ANC, it affects the health of the foetus. The nurses know the cause, but don’t tell us. I once send my daughter-in-law to our clinic from morning up till evening, but her labour was not advancing, hence, we were referred to Nadowli hospital. On arriving, I was told the baby was dead in the womb and could not be extracted at Nadowli hospital. they referred us again to Wa hospital. CS operation was conducted and the dead baby removed, but they never told us of the cause of the death.

**Do record maternal deaths in the community?**

Yes, a woman died not too long with the baby in the womb.

**What caused it?**

She went into labour for long at home. when she was taken to the clinic, she was referred to Nadowli and further to Wa hospital. it was Wa hospital, the death occurred. One time, we had a funeral in one section of the community. When I got there I inquired of the cause and was told she delivered successfully at home but died in few days later. Other adult women said there were still retained matter in the uterus, and we believe it is the retained matter that caused it. It was also a homebirth, so the corpse was not taken for post-mortem investigations. Usually, with home births, we ensure repeated warm water press to get all retained matter removed.

Few months ago, a neighbour complained of abnormality with her and was taken to Nadowli hospital. the midwife in our clinic was not available on this given day. When they arrived at Nadowli, she told the baby was breeched. So she was referred to Wa. In less than three days on admission, the doctor conducted CS surgery and found the baby dead inside the womb. This very operation took the doctor more seven hours in the operation theatre, which brought fear on me as I waited outside. Within a week, a second surgery was carried out on her again to complete the removal of retained matter in her uterus. This created perpetual drops of fluid from the operation area of the body anytime she walked. A third surgical operation was conducted to correct the error of the second and first operations. The essence of the third was to replace raptured intestines caused by the dead baby. When we were discharged, she died a month later.

There was also a case of expectant mother who suffered long-drawn-out sicknesses through the gestation. She became so lean and pale that many didn’t recognise she was still pregnant. She died shortly after she had a live birth. The child is still alive.

We also had instance of our daughter who went galamsey site and met a partner. She conceived whilst at site and was taken to the partner’s hometown. She fell sick in her ninth month for one week and was taken to Nadowli hospital. she died with the baby at the hospital. the midwives said she due to give birth the next day before the death.

**What work do women do in pregnancy?**

The work pregnant woman does depend on her condition of health each day. Hence, if she is healthy, begins by cleaning the compound, go to the borehole for water, cut and carry firewood home, then she will then prepare lunch for herself and the family. Some expectant mothers are able to do all jobs non-pregnant women does. But jobs such as pito brewing, charcoal burning and sale are the dominant income generating activities for all women in the community. Therefore, when one is pregnant, they must still do them in order to survive economically.

**What work do pregnant women do?**

We do all as when not pregnant. They include; cutting firewood, sowing of seeds at farm, cooking, drawing of water, pito brewing, charcoal burning. Some expectant mothers even do weeding with hoe, so as to cultivate food stuff for herself and the children.

**I thought it is men who do the tilling?**

Some men are very lazy .so if you the woman don’t work on the farm, there will be no food stuff to feed on. Other pregnant women engage in their private farm plots to produce grains and groundnuts, solely for sale to make income. Here, rice farms are worked entirely by women. Whether, pregnant or not you must work on the rice farm alongside the rice farm. we risk into farming activities during pregnancy to fend for the children and ourselves. Usually, it’s the women who care for the children. We farm because, some men will run into galamsey site until expectant mother give birth.

**Safety of pregnancy?**

When we don’t engage in hard labour, it will ensure safety. Pregnant who have had difficulty during labour understand regular ANC attendance and adherence to the lessons ensures safety. There women who never receive maternal healthcare throughout the conception, such are the ones who face so much difficulty during childbirth.

**Why does pregnant refuse to receive maternal healthcare?**

Some expectant mothers deliberately refuse to seek maternal healthcare. Others do not want public to know she is pregnant, particularly when there are controversies concerning her conception. They go into hiding until it is critical on their health. This is very common among young women. We are usually asked to commence ANC in the first month of gestation, so that the medications can strengthen us to progress safely and also have safe childbirth.

***Why do some women refuse to take ANC medications?***

Women usually complain it has nauseating smell. Others entertain fears of CS delivery due to baby’s weight. It is a misconception among uninformed pregnant women that intake of ANC medications will lead to unprecedented increase in the weight of the unborn baby which might prevent them from having normal vaginal childbirth. The fear of having CS delivery deter expectant mothers from intake of ANC medicines. *I vomited everything I ate each time I took the medicine. I instead inserted them into muzzle of food and swallowed it, thereby preventing the nauseating reaction to it. It is my strategy to stopping medicine wastage, because I needed it for good health and safe pregnancy.*

**What are your Perceptions about BPCR?**

BPCR is getting your birth kit ready when you are due to give birth. The kit usually contains the following items: new blade, thread, soap, cloth, clean rags, rubber bags for labour bedspread, and parazone or Dettol.

**Do you have any community initiative for emergency referrals in pregnancy?**

There are no initiatives in the community for emergency referrals of any patient including pregnant women.

***How are emergency referrals of pregnant women handled in the community?***

It is usually dependent on the pregnant woman and her family find means of transferring her to the hospital*.*

*In a given day, at about 1am, we went about looking for motorbike to send an expectant mother who suffered complications to Nadowli hospital. it took us about two hours to get a vehicle. When she was admitted, she had a miscarriage. This incident happened in February 2016.*

***Do you have any monetary savings as community or group one can access during emergency referrals?***

we don’t have any of such initiative and community members are willing to lend you money to mourn your corpse but reluctant to support to seek neonatal and maternal healthcare.

**Support family give expectant mothers?**

we encourage expectant mothers to eat recommended food ingredients indicated on the ANC card. We aslo ask them to keeo iff menial jobs particularly those involveing fierce sunshine and fire. Some families help nursing mothers give the baby a wash and also support her prepare meals. In terms of work, expectant mothers do all the activities as before. Some daughter- in-laws do not regard the mother-in-laws so even in pregnancy, we (in-laws) watch them do all hard activities- brew pito and burn charcoal and convey them to the market all alone.

**Do you have traditional beliefs and practices on pregnancies and neonates’ healthcare?**

Yes, we receive care from the clinic and a TBA from Dapuoh. When we go to the clinic and are told the baby is in a breeched presentation, whenever we seek care from the TBA she is able to reposition it. One time, my daughter went to Nadowli hospital and was told she had a breeched presentation of the baby and so was told by the midwife to consult doctor the next day. When she returned home, I urged the husband to support her visit the TBA, and it was corrected. She gave birth safely in the clinic the following month. We also make syrup from boiled leaves and administer on neonate’s when the baby is unresponsive to medications given. Sometimes, they get restored through the traditional treatment.

**Are men involved in maternal healthcare seeking such as the ANC?**

No, they show little or no interest in maternal healthcare seeking. When expectant mother is in labour and the husband is available are the only time he would look for means of transport to support you reach referral facility- Nadowli hospital. if the man’s relatives are available, they support during complications or labour.

**Do we have homebirths?**

some go into labour and refuse to inform the husband or the mother-in-law. So they end up giving birth in at home. other expectant mothers deliberately give birth at home.

*Do pregnant women who give birth at home receive ANC or not?*

Some attend ANC throughout the period of gestation. The nurses will tell them their EDD, yet they refuse to report at the clinic to have childbirth supervised by the midwife. A pregnant woman once had two sets of twins as stillbirths through homebirths, before she then had the third livebirth at the clinic. Some only begin to patronise skilled childbirth only after they had suffered complications or near death experience with homebirth.

**What suggestions will you make for improved maternal and neonatal healthcare?**

We need community education for attitudinal change towards maternal healthcare seeking among families. In this community, a lot of pregnant women do not usually complete ANC course before childbirth.

Homebirths. Recently, a pregnant woman gave birth at home and then conveyed herself to the facility for assessment and baby’s immunisation. I have had six childbirths, but all of them were at the health facility. as I sit, I can’t even supervised home delivery, because I have never witnessed any.

Some women belief so much in “calabash and bowl”. It is a practice involving herb application on the mother whilst she is pregnant or the neonate immediately on delivery. The neonate is also smeared with the same herb to prevent any sickness. Instances like that, when you have not been initiated into same belief system, one who joins the belief must not see your child until after 3 weeks (for males) or 4 weeks (for females). Otherwise, the newborn might fall sick and die if prompt treatment is not provided with the same herb. Others also hang a white robe on the baby’s neck. That child must not also come in contact with the “free baby”. Even when a woman is initiated, she is must not give a helping hand such as to carry water up her head, wood, or any load of any form; to expectant mother who is a non-member of the belief system. The unborn baby will be affected by such interaction and could be delivered as still born if the mother does not receive treatment. When such a baby is born and the mother is unaware and administer treatment, the child could fall sick and die. We have elderly women who provide these herbal belief and practices. We need government’s support to change these outmoded and harmful beliefs and practices. It is unhelpful to expectant and nursing mothers. We have had innocent stillbirths and neonatal deaths in this community caused by these beliefs.

WOGGU OPINION LEADERS

**What work do pregnant women do?**

***At work***

Men who engage solely in farming for livelihood, their wives support in farm labour such as weeding, transplanting of seedlings, fertilizer application and all other activities even when they are pregnant. They partake in activities that are within their ability. When they get tired, they stop and continue the next time they go to farm.

***At home***

expectant mothers draw water from the borehole for household use. They are the ones who do cooking of meals if there is no rival or relative to support her and give the children a bath. During communal labour, expectant mothers are allowed to possibly fetch water for the workers. We do not allow them partake in any hard activity that could affect their health. However, they do activities in moderation.

***Assuming a man has one wife, when she conceives, who cut firewood?***

The expectant mother cuts the firewood because men do not do those kinds of activities. However, when they are due to give birth, the family support in cutting and converting of the wood. *My friend’s wife was into pito brewing as her income earning business. During labour, it was very critical for her to give birth. Meanwhile she was earlier advised during ANC to stop engaging in such activities. After I witnessed that life-threatening incidence, I stopped my wife from pito brewing and instead engaged her in cloth-weaving.*

**What are the causes of maternal deaths in Woggu community?**

*We have had a few cases of recent. My wife died shortly after giving birth to my last born. It was a result of retained content, which I blamed the attendants (adult women who supervised her delivery). However, it is not so common now as in the past four years. Recently, we recorded one maternal death, but she had separated with the husband after conceiving and went to stay with her parents until the death was announced. We are unable to tell the cause of it.*

**What are the causes of stillbirths and neonatal deaths in the community?**

We have had stillbirths, but they usually occur at Nadowli hospital or on their way to Nadowli hospital. But many of such happen to expectant mothers who fail to receive maternal healthcare or turn up early at the clinic during labour. One main issue is with the means of transport during emergency referrals to Nadowli hospital. the delay in reaching the facility leads to wayside stillbirths. Others are caused by the “shaking on the rough road” by the tricycle. It affects the baby by the time they get to the hospital. it forces the baby out untimely, caused by preterm labour. Other expectant mothers reach the facility to discover the baby the unborn is dead already. The preterm births on the wayside die because, there is no way one could resuscitate or incubate them. other stillbirths are caused by breach of our customs. Some married women are unfaithful to their husbands, therefore when they conceive with someone else, they attempt terminating it illegally. Those, usually result to complications or stillbirths when they fail to abort.

The complications which usually lead to stillbirths are caused by some expectant mothers. Some of them bring idols from their mother’s side, which ours do not allow to dwell with. So they rival until the woman loses her baby or return the idols.

***How do you handle emergency referrals of expectant mothers?***

We use motorbike or tricycle motorbike to transfer them. therefore, when we “knock” them on the rough road up to the hospital and are unfortunate, we oftentimes lose the baby. We must emphasise that, it is not all referral cases which result to stillbirths. We had a referral case to Nadowli Hospital by tricycle motorbike, on leaving the community, there was a breakdown of the bike. Whilst the rider looked for a different tricycle in the community to continue with the transfer the women gave birth by the road side. The birth, we believe was a result from the shaking.

**Community initiatives for emergency referrals of pregnant two men and neonates?**

We don’t have any initiative to support expectant mothers during emergency situations. Sometimes we rely on motorbikes and friends support. Meanwhile, there have been instances where the motorbikes and so-called friends and relatives have failed expectant mothers to receive relevant maternal and neonatal healthcare. We hoping government will provide ambulance service for the health centre so we can readily access that for emergency referrals, particularly expectant mothers and babies.

***Do you have public transport system and vehicles in the community one could hire for emergency referrals of pregnant women and newborns?***

We don’t have trotro service connecting the community. For us to hire vehicle, one has to travel to Issa or Tabiesi or Fian, which are all far from Woggu community. Other times if one is fortunate, the drivers are reached via mobile phone call to arrange for them to carry out the transfer to the referral facility – usually Nadowli.

**Why do some women not take up ANC or receive it late?**

The midwife is doing so much here, to ensure all pregnant women receive maternal healthcare and also have their childbirths supervised by skilled professional, there are expectant mothers who stay in the farms during farming season with their family. Such expectant mothers do not receive maternal healthcare during farming season. Others intentionally stay at home until pregnancy is advanced in age before they register for ANC. Another cause of refusal to attend ANC is as result of some mothers’ inability to renew their NHIS card. The card renewal is free, but getting means to Nadowli is usually the challenge.

***Do you have traditional beliefs and practices on maternal and neonatal care?***

Yes, when there is an outbreak of sickness, which might have claimed some lives of newborns, the elders gather at the community shrine to pacify the gods and recommit the lives of all neonates to their care. After the sacrifices, mud is prepared from the shrine and marks made on the foreheads of every community member. Anytime, this is done, the neonatal sicknesses and deaths stop. Usually, measles outbreak can be terrible in the community. When the community lose few lives of newborns, we turn to the gods again for restoration of good health. Before, this is done, we first seek care from the clinic. When expectant mother’s breastmilk ceases to flow, there are practices we carry it out for it to flow so the newborn can feed on.

**Support given to expectant mothers?**

When pregnant women give birth we provide them with food stuff. Some men do support their wives in cutting firewood when they conceive.

**Are men involved in maternal healthcare seeking?**

No, here we don’t do those ones. However, when on referral, we take them to the hospital. Normal ANC, we don’t support or accompany them. we are not used to going to clinic with them. generally, farmers don’t accompany expectant mothers for routine ANC. They are compelled to visit clinic with expectant mothers when she is sick at night. Even, some women do not attend ANC and PNC, how more men?

Recently, the midwife at Woggu clinic referred a nursing mother who never received maternal healthcare until child to Issa health centre. The baby was four months old and fell sick, before she then visited the clinic for her first time since she conceived.

**What groupings or settings are maternal and neonatal health issues discussed in the community?**

It was once the midwife went round the weekly group sittings o inform community based surveillance volunteers to look for some expectant mothers who were referred and but failed to honour the referral. We don’t have health issues as part of our weekly agenda.

**BPCR means?**

When an expectant mother talks about preparedness for birth and complications, it means we are to buy rubber containers for bathing the newborn, purchase cloths and clothes for the baby, acquire some clean linen, buy mosquito nets and soap. However, we don’t support in buying the baby clothes ahead of birth. It is the women who buy most of the items. Men might not even be informed of the need to support purchase the items. We don’t prepare for complications, because we usually don’t hope for it. When it happens, we rise up to look for money and means of transport to send her to the referral facility. How do you prepare for something we don’t know when it is going to arrive? Some pregnant women are unaware of the period of the conception, because they are unable to calculate the date of their last menstrual cycle so the midwife could estimate the expected date of childbirth (EDC).

**Suggestions for improved maternal and neonatal healthcare**

there is an ongoing education by the CHNs, Midwife and the CBHSVs to ensure all expectant mothers receive maternal care so as to prevent referrals. We are doing this because of our distant to Nadowli hospital and Wa hospital so as to prevent stillbirths and maternal deaths.

If all expectant mothers could have alternative activities for livelihoods other than charcoal burning, pito brewing, and strenuous farm labour, it would have been very supportive. The community requires a larger health facility, to qualify for more medicines in the NHIS drug list.

Illiteracy is another chronic disease in Woggu community. Many of the youth end their education at Senior High School level. These teaming migrate into illegal mining sites – “galamsey” leaving their pregnant wives to suffer unto birth. During complications, there is no one to transfer to relevant referral centre. We also need ambulance service at the clinic for easy transfer of expectant mothers to hospital

WOGGU YOUTH

**What is your perception of BPCR?**

BPCR means, ensuring expectant mother receives maternal healthcare. Once she attends ANC, when her expected date of childbirth (EDC) is approaching, the midwife will inform her so proactive measures such staying off some activities, to ensure safety of pregnancy and safe labour. It also means getting the birth kit ready for childbirth. When a pregnant woman is preparing for childbirth and complications, it means she has to take all recommended medications in the right dosage. It also means involving one’s partner in health seeking and purchase of birth kits. Pregnant women who is preparing for birth and complications must stop doing strenuous jobs – weeding, tree climbing, pito brewing, charcoal burning, firewood cutting, so she can prevent herself from complications and sicknesses. Readiness further means, when expectant mother goes into the trimester of gestation, the husband must exempt her from all forms of hard activities. Pregnant women must also have an active NHIS card with her at all times to mean she is ready for emergency.

*My wife just registered for ANC and we are asked to go Nadowli hospital for laboratory examination. I have scheduled a day to take her for the laboratory tests which forms part of the preparedness.* In our community, many women do not respect husbands, so they do not get their involvement in maternal health seeking. The men deliberately shed their responsibility of supporting women to seek maternal healthcare due to the poor attitude shown them. others do not have money to support pregnant women.

**Safe pregnancy?**

Expectant mother eats nutritious food, she does not do stressful jobs, but takes up regular exercise like walking for 2 kilometres at least twice a week. Some expectant mothers are also very lazy. They are not willing to do any job to keep them active.

Some expectant mothers take very high alcoholic drinks. It is one reason for the severe complications among pregnant women. Other men leave their wives and go to galamsey sites in search of money. When business at the site is not as expected for him to remit expectant mother at home, she is forced to do every available hard labour to earn a living.

**I discovered pregnant women do a lot of menial jobs in this community for livelihood?**

Yes, it is true. Expectant mothers whose husbands are very poor engage in menial jobs to cater for her needs and those of the children. For instance, during shea nut gathering, men do not support, therefore, pregnant women from poor homes will have to stress up gathering the nuts. We have had men who returned from galamsey sites and could not recognise their babies because of the long stay at illegal mining site. Teenage pregnancy is also very common in our community. These girls are usually not married to the men at conception. Having no one to care for them after being drop-out, they engage in menial jobs to provide their needs. Pregnant girls who refuse to do these activities suffer so much hunger and may never receive ANC throughout the gestation of the pregnancy.

***Is the ANC not free in your community?***

It is free, but who will give her a ride to Nadowli to carry out laboratory examinations, for her to continue to enjoy the other visits? Even her insurance may not be renewed for her by the “supposed husband”; if expired at the time of conception.

***What are the causes of the teenage pregnancies in the community?***

It is as a result of poverty. For instance, the young girls may not have soap to launder her clothes or she might want to buy new clothes, brazier, sanitary pads, underwear, but does not have the means to acquiring these valuables. Once her parents are unable to provide for her needs, she has to go out to “brothers” for support financially. The so-called brothers capitalise on this and impregnate her for possibly just giving her little money. Meanwhile, we don’t inter-marry, so it is difficult to mention the rightful criminal. Parents do not give young girls sex education. School girls also shun using condoms for these sexual encounters.

***What is the community doing about it?***

the nurses in collaboration with Community Development Alliance (CDA), a non-governmental organisation have embarked on vigorous sex education campaign to help curb the menace of teenage pregnancy in Woggu community. The community do not punish culprits because both culprit and victim are relatives.

The nurses continually encourage young girls in Junior High Schools and Senior High Schools receive family planning devices at the clinic so as to prevent these unplanned pregnancies. However, school girls feel shy to walk to the clinic for family planning pills as well as condom usage. Some girls often say “you cannot eat hacks with the cover”.

What support do family give to expectant mothers?

Families do not support pregnant women in any way. The young men are very lazy, therefore, instead cultivating farmlands to feed their family, many of them run into galamsey mining sites leaving expectant mothers to care for themselves. Pregnant women receive support only during emergency referrals. With such, even in the absence of the husband, any relative available is usually willing to support her to reach the hospital. A lot of young men are unable to care for their wives. Therefore, out of misery, whenever, they conceive, they appear more miserable such that some men avoid their pregnant wife in the midst of people, because, she looks unattractive. Some young men do not want to be identified as the husband of such unattractive expectant mother. I also observed that, many men do not show their wives love again throughout the gestation period. Yes, it is true. Almost all the men are like that. The husbands of pregnant women run to their concubines until we give birth, before they will begin to attend to our sexual needs.

**What community initiatives are there to help improve expectant mothers and newborn healthcare?**

The nurses at our clinic have sensitised us on Community Emergency Transport System and we have made a lot of monetary contributions towards procuring an ambulance. Nevertheless, tricycle motorbikes are the means of transport for emergency referrals of expectant mothers. The client and her husband bears the sole responsibility of getting means of transport to reach the referral centre. There are village savings and loans associations in the community. The savings is of two forms: micro credit for income generating activity and social welfare of members termed, “Kaawonoma”. The social welfare component is accessible to every member who needs money to seek any form of emergency healthcare. It is given without interest on repayments.

***Is it readily accessible?***

Yes, the keys to the money box are kept with three different members, who must ensure they release them to others when travelling or going to farm.

**What traditional beliefs and practices do you have on pregnancies and neonatal healthcare?**

During an outbreak of sickness, particularly on the children, we “cry” to the shrine for protection. Mud is then made from the shrine and marks made on the foreheads of every community member and health is restored. Such outbreaks are common with newborns.

**Suggestions for improved maternal and neonatal healthcare**

Provision of ambulance or even a furnished tricycle at the clinic for emergency referrals of pregnant women and neonates.

Ghana health service (GHS) gave nutrition supplements to enhance health of pregnant women and nursing mothers. It also prevented pregnant women from bringing forth malnourished infants. If this policy could be reinforced, it will be very helpful for the rural women. Poverty does not allow many families to provide nutritious food items for expectant mothers.

Some couples need more education on care for expectant mothers and nursing mothers. We still have marasmus babies in the community.

If we could get all involved in maternal healthcare seeking, it will enable the nurses render sex education and their responsibility over the pregnancy throughout the period.

We further want GHS to reinforce the so-called 24-hour service, that is put on the sign post of the clinic. We don’t get services for 24 hours. We sometimes refer ourselves to Nadowli hospital, because the nurses refuse to wake up at night to attend to emergency cases of pregnant women and newborns. Some deaths of neonates and stillbirths we believe would have always been avoided if they received attention at the clinic first.

NANVILLI/SIRUU NON-PREGNANT WOMEN

**What are the causes of maternal deaths in your community?**

We have had a case of maternal death, but it occurred at Wa hospital after she was referred from Nadowli hospital. it was due to prolonged labour.

**What are the causes of stillbirths?**

Stillbirths occur mostly among expectant mothers who fail to take good care of themselves or risk into charcoal burning activities. There was another issue of local oxytocin. It was administered prematurely which led to the death of infant a few minutes after delivery on the wayside to the health centre.

**What work do expectant mothers do in your community?**

Expectant mothers support the husbands in farm labour. Usually, they do the sowing of seeds and other less difficult jobs. Expectant mothers also process sheanuts, brew pito to earn income. We also cut firewood for domestic consumption. Others also risk into charcoal burning and sale.

At home

Expectant mothers also draw water for household use, prepare meals for the family and farm labour and basic cleaning of the compound. They further wash clothes of the family.

**Safe pregnancy?**

It is expectant mothers’ responsibility to attend ANC regularly. When she completes the course of ANC and take all the prescribed medications appropriately, then we say her pregnancy is safe and will ensure her safe delivery. Pregnant women who eat vegetables, fruits and general balanced meals will have safe pregnancies. She would have also had enough foodstuff to sustain her in the postpartum period up to about six months, within which time she might be able to do any menial job because of the breastfeeding of the baby.

**BPCR means**

BPCR entails buying all the items needed in a birth kit – soap, linens, cloths, blade, thread, and also get food ingredients in advance before she gives birth. Alternatively, she would live on these items when referred to Nadowli or Wa hospitals during childbirth. You need to cut and store enough firewood for use after childbirth.

***Won’t your husband cut the firewood for you?***

No, men do not support in cutting and conveying firewood home.

**Community initiatives for improved maternal and neonatal healthcare**

We have community volunteers who receive routine training by the nurses to conduct ANC defaulter-tracing of expectant mothers. We as volunteers also support in getting expectant mothers reach health facility on time for care. We have a first aid box and pain killers we sometimes administer before taking them to the clinic. The community has a social welfare fund from which one can borrow to carry out swift transfer of emergency referral of expectant mother, and then refund later, although we have only one tricycle motorbike in the community.

**Traditional beliefs and practices on maternal and neonatal healthcare**

We have a local “ladle” herbal medicine for correcting breech presentation. The herbs are burn into coal and mixed with shea butter and then put into the “calabash ladle”. Anytime a woman complains of having shortness of breath, or feel baby is wrongly positioned, we apply this herbal treatment and she gets well instantly. The midwife has discouraged us from this practice, though we still do it. We particularly administer these, when expectant is unable to foot in that condition to the health centre.

Babies and children who suffer convulsion are also given “concocted syrup” from boiled leaves as treatment. Some do recover, others are rushed to the clinic before they are restored to good health. Some expectant mothers also apply “cold” and “hot” local oxytocin.  *I am into it, but I only sell what my sister gives me. I personally do not know how she obtains the herbs.*

***Do expectant mothers receive care from the clinic and TBAs?***

*Yes, they receive maternal healthcare from the clinic and but on critical situations, they also receive the oxytocin. However, the “hot” local oxytocin is administered when labour commences, to facilitate progress of labour.*

**What are the causes of homebirths?**

We have had cases of homebirths among pregnant women. It is common among women whose labours do not keep long (precipitate labour). Also, when the “hot” oxytocin is administered so early, she will definitely give birth at home or on the roadside. Nevertheless, every expectant mother now makes frantic efforts to reach the clinic for childbirth. *My twins were brought forth at home before I was taken to the clinic for immunisation. I didn’t expect labour to commence that day.*

***How do you dispose off the placenta during homebirths?***

we burry them in our surroundings. Others put it their toilets (man hole). Sometimes, we take the palcenta with the nursing mother and the newborn together to the clinic and drop it into the placental pit at the clinic. Traditionally, when we want to bury it, we first dig the pit ad two adult women and the new nursing mother will all hold together and drop it into the pit with backwards facing it.

***Why don’t you face the pit when dropping?***

It is our tradition. It is also believed we could become blind if we face it.

**Are men involved in maternal healthcare seeking?**

No, the only instance, men get involved is when expectant mother is referred to Nadowli hospital or Wa hospital. Expectant mothers who are lucky receive support from their husbands to Nadowli during laboratory tests.

**Suggestions?**

We need a spacious health facility. the clinic serves six different communities, therefore there is pressure in the facility. we have only one midwife. If more could be posted to the facility it will be very helpful. If we could get income generating activities as women, so we could stop engaging in risky activities – charcoal burning, pito brewing, for livelihoods. Government should further intensify the immunisations given to expectant mothers and neonates.

NANVILLI/SIRUU OPINION LEADERS

**What work do expectant mothers do in your community?**

***At work***

We encourage all families to exempt expectant mothers from hard farming activities, however, they partake in seeds sowing. They do all jobs non-pregnant are able to carry out, but in moderation and within their individual ability.

***At home***

Pregnant women prepare food for family, sweep the compound, draw water from the borehole. Nonetheless, these activities are done based on her health condition and ability to do them. it is not compulsory for pregnant women to force themselves into certain activities that could affect her negatively. They choose the kind of work they are able to do. Remember, women are married to support men in the farm work and household activities. But when they are pregnant, they do jobs that they can. They must do income generating activities – pito brewing, shea butter extraction, others who are strong partake in charcoal burning and sales. However, we have stopped women from climbing trees for dried firewood and fruit harvesting. We discourage expectant mothers from carrying heavy load too.

**Pregnancy safety?**

When pregnant women do not burn charcoal, and also brew pito in moderation, the pregnancy is safe. Cordial relationship at home and in the marriage also ensures safety of pregnancy. For instance, when they heed to the husbands’ advice and do not engage in risky menial and stressful jobs – such as carrying heavy load, intense she abutter processing, it will promote healthy pregnancy.

**What are the causes of maternal deaths in the community?**

We have recorded two cases in 2015 but they were referred to Nadowli hospital and further to Wa hospital where they died. One of them was from prolonged labour. The labour took three days before she reached Wa hospital where CS was poorly conducted leading the death. The other one, we were not informed of the cause. One major cause is pregnant women leaving home for galamsey mining activities. Many have lost their lives in neighbouring communities though not here. Some pregnant women do not also attend antenatal care.

**Community initiative?**

we have a social welfare fund for the community members to access during emergency referrals of all sick cases. The money is refundable within three months without interest payments. The money can be used to hire means of transport – tricycle motorbike and also pay hospital bills.

**Community meetings/groupings where MNH care issues are discussed?**

We have weekly VSLA meetings among women and men. During outbreak of diseases such as CSM, yellow fever and any other public health issue, the CHN visits the groups to create awareness on preventive measures. Aside these, MNH care is not part of our routine meetings.

**Do you have traditional beliefs and practices on MNH care?**

We have the calabash ladle practice for breech presentation of foetus. Adult women administer the herbal medicine to the ladle and make a line across the tummy of the expectant mother. Anytime, this is done, the foetus repositions itself. When pregnancy is overdue, there are herbs – local oxytocin, that is applied on the expectant mother’s tummy and orally, triggering immediate labour. When a neonate falls sick, we have some leaves that are boiled into “syrup” and administered orally and through bath to restore good health. The “syrup” is usually administered when the clinic is unable to treat it.

**Are there expectant mothers in the community who do not receive maternal healthcare?**

For now, no. Pregnant women might take up ANC late or fail to complete it. Usually, we record late uptake when they are not aware of the conception. We have health volunteers who ensure every expectant mother attends ANC.

**Why do some pregnant women have homebirths?**

Women do give birth at home in the community, but this is common among women who do not have long labours before childbirth. Others give birth on their way to the clinic, however, we encourage all to have their childbirths at the health facility.

**Suggestions?**

There is no vehicle in the entire community for us to access during emergency referrals of pregnant women. The only means of transport is the tricycle motorbikes and motorbikes. It becomes a serious challenge when pregnant women are referred to Nadowli hospital. unless one travel to Kaleo, Nadowli or Wa before getting a vehicle to execute the transfer. It is reason, all referrals are carried out using the tricycle or motorbikes.

There is no light in the clinic. the facility is powered from a generator, and fuelled by whoever that sends a case at night. It is not everyone who can fuel the generator for the midwife to conduct childbirth or provide treatment to the patient.

The community further needs awareness creation, to ensure all pregnant women seek maternal healthcare. The men do not also support pregnant women. Therefore, if there be a way of getting them involved, it will help.

The facility has only one midwife. Government should more midwives to the facility.

NANVILLI/SIRUU YOUTH

**BPCR means?**

getting birth kit ready. The items usually include, soap, powder, cloths, Dettol, linen or clean rags into a portable handbag or rubber bag. When it is left with one or two weeks to her EDC, expectant mother has to buy these items. The Dettol is used by the midwife to wash her hands after childbirth. The rubber bag is for spreading on the labour bench. Preparedness also include saving little monies towards referral to Nadowli Hospital. we have a social welfare fund if one has not saved personally. But that fund is not reliable. It involves so much bureaucracy before you finally get the money. The box with the money has three (3) padlocks which are kept with three members in the community. Hence in the absence of one of the key keepers, the box cannot be opened. One would have to wait until they all return. The keeper of the box does not also keep any of the keys. So the 3 keys-keepers could be available whilst the box-keeper is unavailable and the money cannot still be accessed.

**Safety pregnancy.**

A pregnant woman who attends ANC regularly and takes the dosage of the medication appropriately will have safe pregnancy. It a woman does not work at farm, or engage in “naked fire” business such as pito brewing, charcoal burning, shea butter extraction, the pregnancy will be safe. When there is peace between expectant mother and the husband or the family, she will have safe pregnancy. Safe pregnancy is further achieved when pregnant women maintains explicit personal hygiene and conscious of her meals also.

***What cause the quarrels?***

Sometimes, expectant mother may be asked to take egg or meat to maintain her HB; however, women do not rear fowls, attempts to pick egg from the hen coop could lead to quarrels for the husband. Usually, in the first pregnancy (prim gravida), husbands are willing and ready to satisfy all the demands of expectant mothers. But subsequent pregnancies, turns out to be burdensome on them.

**How many times do you have to attend ANC through the gestation period?**

Normal ANC require four visits. However, high risk women are required to make more than four ANC visits.

**Why do expectant mothers delay in receiving ANC?**

When we conceive and attend ANC, the midwives first give us the ANC card to go Nadowli hospital for laboratory investigations. If your husband does not have a motorbike to take us there, it could take several weeks to commence ANC actual due to delay in conducting laboratory tests. the delay in carrying out the laboratory tests further affects the number of vaccinations and medications received. The inability to complete four plus (4 +) ANC attendance are our husbands.

**What support do family give to expectant mothers?**

One support some men give is peace of mind for her to go through the gestation period successfully. *Anytime my wife conceived, I pick her with my motorbike to receive laboratory tests at Nadowli, so she can commence ANC actual on time. I also ensure her NHIS card is active. I have taken her to ANC before in her recent conception*. We also support them by reducing the volume work whenever we go to farm. she is allowed to do minimal activities. Anytime, I conceived, I am unable to do most work, particularly during my third trimester. Therefore, my husband supports me to draw water from the borehole. He often also helps with cooking and clothes washing. But these forms of support are very rare in this community.

**What community initiatives are there for emergency referral of pregnant women and newborns?**

There is no community initiative for emergency referrals. When a pregnant woman is sick or in labour, the husband or the family have to hire a tricycle to transport her to the clinic. if it is referred further to Nadowli, it is the same tricycle one would hire to seek relevant care at the referral facility. referrals are made direct to Nadowli.

**Traditional beliefs and practices?**

Local oxytocin helps facilitates the labour. It is even a major cause of many home and roadside childbirths. It is very potent for safe and fast childbirth.

**Suggestions?**

Purchase of ambulance for the clinic to facilitate referrals to Nadowli or Wa hospitals.

We also need and irrigational dam which to provide income generating activities and reduce the dependence on charcoal burning and pito brewing activities, by expectant mothers and families for livelihoods.

The health centre serves a very wide threshold. If it could be upgraded to a polyclinic to render many essential MNH services, it will reduce the burden of healthcare seeking at Nadowli.
